# Design, synthesis, and preliminary evaluation of a potential synthetic opioid rescue agent

**DOI:** 10.1186/s12929-021-00758-y

**Published:** 2021-09-09

**Authors:** Sidnee L. Hedrick, Dan Luo, Sophia Kaska, Kumar Kulldeep Niloy, Karen Jackson, Rupam Sarma, Jamie Horn, Caroline Baynard, Markos Leggas, Eduardo R. Butelman, Mary Jeanne Kreek, Thomas E. Prisinzano

**Affiliations:** 1 Department of Pharmaceutical Sciences, College of Pharmacy, University of Kentucky, 789 S. Limestone, Lexington, KY 40536 USA; 2 Center for Pharmaceutical Research and Innovation, College of Pharmacy, University of Kentucky, Lexington, KY 40536 USA; 3 Laboratory on the Biology of Addictive Diseases, The Rockefeller University, New York, NY 10065 USA

**Keywords:** Structure–activity relationship, MOR antagonist, Fentanyl, Naltrexone, Antinociceptive activity

## Abstract

**Background:**

One of the most prominent opioid analgesics in the United States is the high potency agonist fentanyl. It is used in the treatment of acute and chronic pain and as an anesthetic adjuvant. When used inappropriately, however, ingestion of just a few milligrams of fentanyl or other synthetic opioid can cause opioid-induced respiratory depression (OIRD), often leading to death. Currently, the treatment of choice for OIRD is the opioid receptor antagonist naloxone. Recent reports, however, suggest that higher doses or repeated dosing of naloxone (due to recurrence of respiratory depression) may be required to reverse fully fentanyl-induced respiratory depression, rendering this treatment inadequate. To combat this synthetic opioid overdose crisis, this research aims at identifying a novel opioid reversal agent with enhanced efficacy towards fentanyl and other synthetic opioids.

**Methods:**

A series of naltrexone analogues were characterized for their ability to antagonize the effects of fentanyl in vitro utilizing a modified forskolin-induced cAMP accumulation assay. Lead analogue **29** was chosen to undergo further PK studies, followed by in vivo pharmacological analysis to determine its ability to antagonize opioid-induced antinociception in the hot plate assay.

**Results:**

A series of potent MOR antagonists were identified, including the highly potent analogue **29** (IC_50_ = 2.06 nM). Follow-up PK studies revealed **29** to possess near 100% bioavailability following IP administration. Brain concentrations of **29** surpassed plasma concentrations, with an apparent terminal half-life of ~ 80 min in mice. In the hot plate assay, **29** dose-dependently (0.01–0.1 mg/kg; IP) and fully antagonized the antinociception induced by oxycodone (5.6 mg/kg; IP). Furthermore, the dose of **29** that is fully effective in preventing oxycodone-induced antinociception (0.1 mg/kg) was ineffective against locomotor deficits caused by the KOR agonist U50,488.

**Conclusions:**

Methods have been developed that have utility to identify enhanced rescue agents for the treatment of OIRD. Analogue **29**, possessing potent MOR antagonist activity in vitro and in vivo, provides a promising lead in our search for an enhanced synthetic opioid rescue agent.

**Supplementary Information:**

The online version contains supplementary material available at 10.1186/s12929-021-00758-y.

## Background

**Fig. 1:**
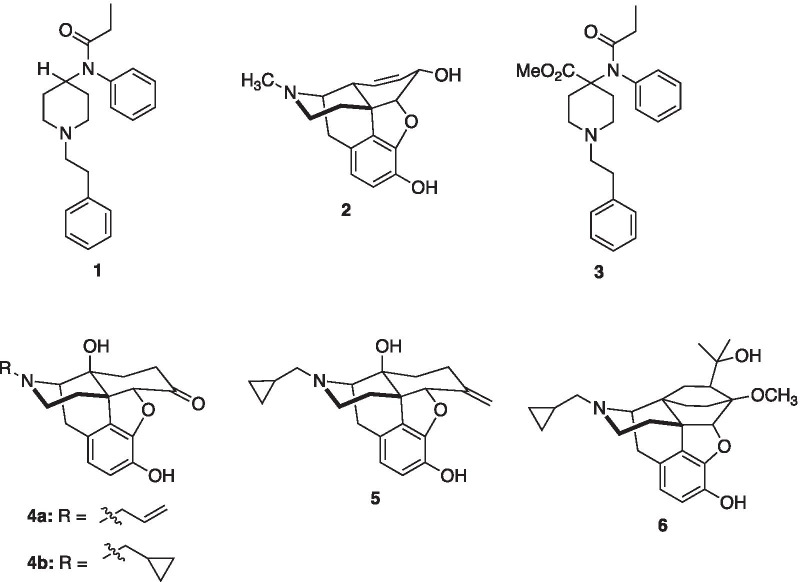
Structures of fentanyl (**1**), morphine (**2**), carfentanil (**3**), naloxone (**4a**), naltrexone (**4b**), nalmefene (**5**), and diprenorphine (**6**)

One of the most prominent opioid analgesics in the United States is the synthetic opioid fentanyl (**1**) (Fig. 1) [1]. It is used in the treatment of acute and chronic pain and as an anesthetic adjuvant [2, 3]. Originally synthesized in 1960, fentanyl (**1**) is approximately 100 times more potent than morphine (**2**). Intravenous fentanyl has an LD_50_ of 2.91 mg/kg in rats [4]. Among clinicians, fentanyl rapidly replaced morphine as an anesthetic for surgeries during the 1970s due to its more rapid onset, higher potency, and limited cardiovascular risks compared to morphine [5–7]. Currently, there are several FDA-approved fentanyl analogues for medical and veterinary purposes, including the ultra-potent carfentanil (**3**) (approximately 10,000 times more potent than morphine) [8]. Misuse of fentanyl (and fentanyl analogues) has been estimated to be responsible for 48,000 (out of a total of 83,335) overdose deaths in the 12 months ending in June 2020, significantly contributing to the national opioid health crisis [9].

The high potency MOR agonist fentanyl (**1**) is also considered an incapacitating agent, a chemical that produces a disabling condition that persists for hours to days after exposure has occurred [10]. Incapacitating agents were studied during the Cold War when it was assumed that incapacitating the enemy would impact them more because these individuals would not only become unavailable for duty but also because they would consume more logistical resources relating to their evacuation [11]. In October 2002, the Russian military used a mysterious “gas” to incapacitate Chechen rebels who had taken 800 hostages at a Moscow theater [12]. Unfortunately, more than 120 of the hostages in the theater died and more than 650 of the survivors required hospitalization. The available evidence strongly suggests that a combination of a potent aerosolized fentanyl derivative, such as carfentanil (**3**), and an inhalant anesthetic, such as halothane, was used by the Russian military. Preparation of medical teams with suitable stores of effective antidotes would likely have lessened the loss of life.

Chemically, synthetic opioids are highly toxic organic solids that may be encountered as injectable powders, liquids, nasal sprays, dermal patches and pills. The particle size of synthetic opioid powders typically ranges from 0.2 to 2.0 μm, and the powders are easily aerosolized, presenting primarily a respiratory hazard. A secondary dermal hazard exists if there is direct skin contact with large bulk amounts of concentrated threat materials [13]. Due to its high potency, ingestion of just a few milligrams of fentanyl or other synthetic opioid can be deadly to an opioid-naïve individual or an unsuspecting “recreational” drug user upon acute exposure. Furthermore, first responders who come in contact with free base fentanyl analogues are at significant risk for life-threatening toxicities [14].

Currently, there are three FDA-approved opioid antagonists that have potential to reverse the effects of fentanyl in humans: naloxone (**4a**), naltrexone (**4b**), and nalmefene (**5**). Naloxone is approved for administration by a variety of routes, including intravenous, intramuscular, subcutaneous and intranasal; sublingual and buccal formulations are under development [15]. However, recent reports suggest that higher doses or repeated dosing of naloxone (due to recurrence of respiratory depression) may be required to reverse fully fentanyl-induced respiratory depression [16–18]. These findings have been recently confirmed in mice where naloxone less readily reverses respiratory depression by fentanyl compared with morphine [19].

Recently, diprenorphine (**6**) was shown to equally reverse both fentanyl and morphine depression of respiration in mice [19]. Previous studies have also shown that diprenorphine could be used to reverse the effects of opioids for which naloxone does not effectively or reliably reverse the narcotic effects [20]. Presently, diprenorphine is approved for use in veterinary medicine to reverse immobilization of wild and exotic animals by etorphine (reported to be from 1000 to 80,000 times more potent than morphine depending on the parameter measured) or carfentanil [21].

The reason for why diprenorphine is more effective at antagonizing fentanyl than naloxone is not known. One potential reason for the greater effectiveness of diprenorphine in antagonizing fentanyl-induced respiratory depression could be the enhanced potency of diprenorphine compared to naloxone [20]. However, it has also been speculated that the higher lipophilicity and/or an alternative mode of binding at μ opioid receptors (MORs) than naloxone may contribute [19].

Collectively, these studies suggest that a synthetic opioid rescue agent superior to naloxone is needed. An optimized profile of such a compound would have the following characteristics: (1) enhanced potency and lipophilicity; (2) in vivo pharmacokinetics and physiochemical/metabolic properties necessary for multiple formulations; and (3) few off-target effects and little toxicity. Here, we report our initial work toward identifying such a rescue agent.

## Materials and methods

### Synthesis of naltrexone analogues

A series of opiates (**7**–**30**) were prepared from commercially available naltrexone hydrochloride (Mallinckrodt, St. Louis, MO) modified in three positions: (1) the C3-phenol; (2) the 14β-hydroxyl group; and (3) the C6-keto group. Compounds **7** [22], **8** [23], **9** [24], **10** [25], **11** [25], **12** [25], **13** [26], **15** [27], **17** [28], **18** [29], **19** [28], **23** [30], **24** [31], and **27** [32] were prepared by previously published procedures. Opiates **14**, **16**, **20**–**22**, **25**, **26**, **28**–**30** were prepared using a general sequence of protection, synthetic elaboration, and deprotection. Experimental details of the synthesis of the series of opiates and their corresponding identification data can be found in Additional file 1.

### Compounds

Morphine sulfate pentahydrate, fentanyl hydrochloride, oxycodone hydrochloride, naltrexone hydrochloride, β-funaltrexamine hydrochloride, clocinnamox mesylate, SNC-80, naltrindole hydrochloride, U50,488H, and Salvinorin A were kindly provided by the National Institute on Drug Abuse Drug Supply Program. Naloxone hydrochloride dihydrate and *nor*-binaltorphimine dihydrochloride were purchased from Sigma-Aldrich Chemical Co. (St. Louis, MO, USA). All other chemicals used were purchased from commercial sources and are of analytical grade.

### In vitro experiments

#### Cell lines and cell culture

The cAMP Hunter™ CHO-K1 stably expressing the human μ opioid receptor (MOR) (OPRM1, catalog # 95-0107C2), human κ opioid receptor (KOR) (OPRK1, catalog # 95-0088C2), and the human δ opioid receptor (DOR) (OPRD1, catalog # 95-0108C2), were purchased from Eurofins DiscoverX (Fremont, CA) and maintained in F-12 media supplemented with 10% fetal bovine serum (Life Technologies, Grand Island, NY), 1% penicillin/streptomycin/ʟ-glutamine (Life Technologies), and 800 µg/mL Geneticin (Mirus Bio, Madison, WI). All cells were grown at 37 °C and 5% CO_2_ in a humidified incubator.

#### Forskolin-induced cAMP accumulation

The agonistic activities of test compound were determined as previously described [33]. Briefly, the aforementioned cAMP Hunter cell lines were detached from cell culture plates using nonenzymatic cell dissociation buffer (Life Technologies) and plated at 10,000 cells/well cell density in 384-well tissue culture plates, and then incubated at 37 °C overnight. 5 mM Stock solutions of all test compounds in DMSO (Alfa Aesar, Ward Hill, MA) were prepared followed by serial dilutions in DMSO resulting in 10 dose points at a 100× concentration. Assay buffer [Hank’s Buffered Salt Solution (HBSS, Life Technologies) and 10 mM HEPES (Life Technologies)] with forskolin (Eurofins DiscoverX) were used to dilute the serial dilutions to a working 5× concentration resulting in a concentration of 100 µM forskolin and 5% DMSO (v/v%). The cells were incubated with the test compounds at 37 °C for 30 min and the HitHunter cAMP assay for small molecules assay kit (Eurofins DiscoverX) was used according to the manufacturer’s directions for cAMP detection.

The antagonist activities of test compound were determined in a similar manner except only assay buffer was used for the dilution of test compounds to 5× working solutions. The cells were pre-treated and incubated with vehicle or test compounds for 15 min followed by the addition of selected agonists at their EC_50_ or EC_90_ dose in the presence of forskolin. The cells were further incubated at 37 °C for another 30 min.

Luminescence was quantified using the BioTek Synergy H1 hybrid reader and Gen5 software (BioTek, Winooski, VT). Data were blank subtracted with vehicle controls, normalized to forskolin controls, and analyzed with nonlinear regression by GraphPad Prism 8 (GraphPad, La Jolla, CA). For the antagonist assay, further normalization to selected reference antagonists (naltrexone (**4b**) for MOR and DOR cells, *nor*-BNI for KOR cells) was used to determine the I_max_ of test compounds.

Potent and efficacious MOR antagonists were tested further by Schild analysis [34], which was done by generating fentanyl dose–response curves in the absence and presence of three doses of test compounds. Data were analyzed by nonlinear regression with the Gaddum/Schild EC_50_ shift function in Prism. Compounds with Schild slope close to 1 were considered competitive and pA_2_ values were calculated (constraining both HillSlope and SchildSlope to 1). The equilibrium dissociation constant (K_e_) values were calculated as well using the formula:

$${\text{K}}_{{\text{e}}} \, = \,\left[ {\text{L}} \right]/\left[ {\left( {\text{A}}^\prime/{\text{A}} \right) - {1}} \right];$$

[L] is the concentration of antagonist and Aʹ and A are the EC_50_ of fentanyl in the presence or absence of a single dose of the antagonist.

### In vivo studies

#### Pharmacokinetic (PK) study

Adult male C57BL/6J mice were purchased from Jackson Laboratories (Bar Harbor, ME). The animals were housed in polyethylene cages and given food and water ad libitum. Animals were administered **29** by intravenous, oral gavage or intraperitoneal (IV, PO or IP injection). Two groups of mice (n = 3/group) were sampled three times each via the saphenous vein. Whole blood samples were collected into heparinized pipet tips, centrifuged at 4300×*g* for 2 min to isolate plasma and transferred onto dry ice. Plasma samples were stored at − 80 °C until processing. Separately, for brain biodistribution studies, five mice per time-point were administered **29** via the IP route and a single blood sample was collected from each animal via intracardiac puncture prior to perfusing with ice-cold saline for 5 min before removing the brain. All animal procedures were conducted in accordance with The Guide for the Care and Use of Laboratory Animals (National Academic Press, 1996) and approved by the IACUC (Institutional Animal Care and Use Committee) at the University of Kentucky and at the Rockefeller University.

#### Sample processing

Experimental plasma samples were thawed at 37 °C for 3 min and vortex mixed. A 5 µL aliquot of each plasma sample was added to 5 µL internal standard (100 ng/mL naltrexone (**4b**) in methanol:water (1:1, v/v)) and 10 µL blank mouse plasma. Samples were vortex mixed, then treated with 4× volume (60 µL) of 0.1% formic acid in methanol to precipitate proteins. The samples were vortex mixed (5 s) then centrifuged at 13,000×*g* for 15 min at 4 °C. The resulting supernatants were collected into amber HPLC vials fitted with 200 µL glass inserts and immediately analyzed for analyte content by LC/MS–MS.

Brains were excised and sectioned at the sagittal plane. Half of the brain from each animal was homogenized (1:1, w/v) with phosphate buffered saline. Each brain homogenate aliquot (20 µL) was added to 5 µL of internal standard spiking solution (100 ng/mL naltrexone (**4b**) in methanol:water (1:1, v/v)) and vortex mixed. Proteins were precipitated by addition of 80 µL of 0.1% formic acid. Samples were vortex mixed (10 s) and stored at − 20 °C for 20 min prior to centrifugation at 13,000×*g* for 15 min at 4 °C. The resulting supernatants were collected into amber HPLC vials fitted with 200 µL glass inserts and immediately analyzed for analyte content by LC/MS–MS.

#### Calibrator, quality control sample preparation

All stock solutions were prepared in methanol at concentration approximately 1 mg/mL for **29**, and naltrexone (**4b**) (Internal Standard). All working solutions were generated by diluting the stock solutions of all compounds in methanol:water (1:1, v/v). Calibration curves and quality control (QC) samples were prepared, and analyses proceeded following assessment of QC concentrations to determine system suitability.

For the analysis of total amount of **29** in the plasma samples, calibration curve was generated with **29** drug spiked to mouse blank plasma. Calibrators (0.25–1000 ng/mL) were prepared by the addition of 5 µL of appropriate spiking solution into 100 µL blank plasma followed by vortex mixing. Quality control samples (0.75, 25, 500, 850 ng/mL) were prepared from an independent second stock in a similar fashion. For the analysis of **29** in brain tissue samples, calibration curve was generated with **29** drug spiked to blank mouse brain tissue homogenate using a calibration curve (0.5–372 ng/mL) and quality control samples (0.75, 13.3, 25, 266 ng/mL). The limit of quantitation for plasma was 0.4 ng/mL and for brain tissue 1 ng/mL.

#### LC–MSMS analysis

All samples were analyzed for the transitions m/z 382.9 → 323.2 (**29**), and m/z 342.3 → 270.2 (naltrexone (**4b**) ISTD) by LC–MSMS. Analyte and internal standard contained in 4 µL sample injections were eluted from a Waters XBridge C18 (3.5µ, 4.6 × 150 mm; oven temp. 40 °C) analytical column with a 0.1% formic acid in water (Mobile Phase-A): 0.1% formic acid in acetonitrile (Mobile Phase-B) gradient. The flow rate was consistent at 0.7 mL/min while the gradient progressed from an initial 0.5 min hold at 35% Mobile Phase-B increased linearly to 90% over 3 min. The 90% Mobile Phase-B was maintained for 2.5 min before returning to the initial 35% over a 0.1 min linear ramp. The column was equilibrated at 35% organic for 1.9 min. The total run time was 7.5 min. Positive-mode ESI Turbo V^®^ source and MS gas, temperature and voltage potential settings were based on optimized parameters determined prior to analysis using infusions of 1000 ng/mL drug standards in 50:50 mobile phase mixture mixed with LC effluent for a total 0.6 mL/min flow rate. Flow-dependent parameters were: CUR = 35/ISV = 5500/TEM = 550/GS1 = 65/GS2 = 65/Horizontal probe position = 7/Vertical probe position = 0.5). The compound dependent parameters for the m/z 382.9 → 323.2 (**29**), transition were DP of 30, EP of 10, CAD of 12, CE of 24 and CXP of 15, whereas optimal m/z 342.3 → 270.2 (naltrexone (**4b**) ISTD) transition intensity for ISTD was achieved at DP of 30, EP of 10, CAD of 12, CE of 37 and CXP of 20. Calibrators, quality control samples and experimental sample sequences consisted of single randomized experimental sample injections flanked by sets of blanks, and calibrators. A calibration curve was constructed by weighted (1/x^2^) polynomial regression analysis of analyte concentration to analyte peak area using GraphPad Prism software (Ver 8.4.3).

#### Pharmacokinetic analyses

All data sets were analyzed using Phoenix WinNonlin (Certara). A 2-compartment mammillary model was simultaneously fitted to all plasma concentrations obtained from intravenous, oral, and intraperitoneal administration of **29**. The oral and intraperitoneal bioavailability was also estimated. Parameters were estimated using population modeling with quasi-random parameter estimation method (QRPEM). Non-compartmental analyses (NCA) for sparse sampling methods were conducted to estimate the area under the time-concentration curves (AUC), and the apparent half-life of **29** in plasma and brain compartments following intraperitoneal administration.

#### Thermal antinociception studies

Adult male C57BL/6J mice (Jackson Laboratory, Stock #00064) were studied for oxycodone-induced thermal antinociception with the hot plate assay. The apparatus was a model 39D Hot Plate Analgesia Meter (IITC Life Science, Woodland Hills, CA) used at a temperature of 54 °C ± 0.5 °C. Individual mice were placed inside a cylindrical transparent Plexiglas enclosure (30.6 cm height × 19.4 cm diameter) which was placed on top of the hot plate. Prior to experimental study, mice underwent a habituation session, in which they were placed on the hot plate apparatus at room temperature for two 1-min periods, separated by ~ 10 min. At least 1 day after room temperature habituation, mice were placed on the hot plate for two baseline latency determinations to the 54 °C ± 0.5 °C hot plate temperature. The mouse was removed from the plate when a withdrawal response was observed. A response was recorded as a jump or hind paw lick, with a maximum allowed latency of 45 s, timed manually by stopwatch. If an animal did not exhibit a response by the 45 s cutoff, it was removed from the hot plate, and this value was assigned for data analysis. The experimenter was “blind” as to the experimental conditions under study (e.g., whether the pretreatment was **29** or vehicle). “Blinding” was carried out by using coded labels for solutions. The codes were changed across experiments.

Separate sessions in the same mice were separated at least 96 h from each other. Each session commenced with two baseline withdrawal latency determinations, separated by ~ 10 min.

After the baseline determination, the mouse was injected with vehicle or **29** (IP) at a specified pre-treatment time, and then with vehicle or oxycodone 5.6 mg/kg (IP). The mouse was tested in a time course procedure with latencies determined at predetermined times (15-, 30-, 60- and 120-min post oxycodone injection). If at any of these times the mouse reached the cutoff latency (45 s) without a nocifensive response, it was removed from the hot plate and the cutoff value was assigned for data analysis. The cylinder and hot plate were wiped with water between mice, as needed. The doses and times of oxycodone administration were based on pilot and published studies [35].

#### Antagonism of 29 against oxycodone-induced antinociception

The antagonist potency of **29** was examined with different doses of **29** (0 [vehicle], 0.01, 0.032 and 0.1 mg/kg) administered 30 min prior to oxycodone (5.6 mg/kg). Based on these data, the time course of antagonist effects of **29** (0.1 mg/kg) was examined by administering this compound at different times (15, 30, 120, 240 min, and 24 h) prior to oxycodone (5.6 mg/kg).

#### Antagonism of 29 in preventing locomotor activity deficits caused by the KOR agonist U50,488

These studies (in C57BL/6J mice from the Jackson Laboratory) focused on the effectiveness of **29** in preventing decreases in locomotor activity caused by the KOR agonist U50,488 (10 mg/kg, IP) over 90 min. This dose and duration of monitoring period was based on recently published studies [36]. Mice were placed individually in rectangular transparent plastic cages (19.7 cm width × 41.3 cm length × 20.3 cm height) with bedding identical to that in the home cage. Each cage was in a photocell frame with an array of perpendicular photocell beams (SmartFrame; Kinder Scientific, Poway, CA). Beam breaks caused by the mouse were quantified through a computer interface. Mice were habituated to this apparatus for a 60-min session. Consecutive experiments in the same mice were separated by at least 72 h. Vehicle or **29** was injected 30 min prior to U50,488 (10 mg/kg). Immediately after the U50,488 injection, each mouse was placed in a locomotor activity cage for a 90-min period.

#### Statistical analyses

The hot plate locomotor activity data were analyzed after conversion to percent of maximum possible effect (%MPE) by the standard equation:

$$[\left( {{\text{Cutoff latency}}\, - \,{\text{Test latency}}} \right)/\left( {{\text{Cutoff latency}}\, - \,{\text{Baseline latency}}} \right)]\, \times \,{1}00\% .$$

The locomotor activity data were analyzed as beam breaks over 15-min bins. Data were analyzed with 2-way repeated measures or mixed effects ANOVAs, followed by appropriate post-hoc tests (GraphPad Prism software).

#### Drugs

**29•**oxalate was dissolved daily in sterile water vehicle for all pharmacodynamic studies. **29•**oxalate was dissolved in saline (1 mg/mL) for pharmacokinetic studies and in methanol (0.84 mg/mL) for analytical stocks. The MOR agonist oxycodone HCl (Sigma-Aldrich) was dissolved in sterile water vehicle. All injections were carried out IP in a volume of 10 mL/kg body weight. The KOR agonist U50,488 (Sigma-Aldrich) was dissolved in sterile water. All injections for antinociception and locomotor studies were made by the IP route at a volume of 10 mL/kg body weight.

## Results

### Chemical synthesis

**Scheme 1:**
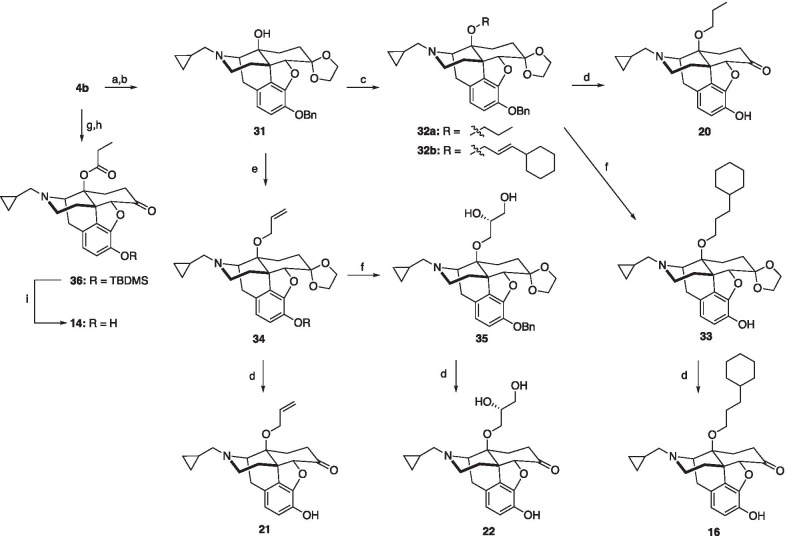
Synthetic route to C14 analogues **14**, **16**, and **20**–**22**. *Reagents and conditions* (a) K_2_CO_3_, BnBr, DMF; (b) (CH_2_OH)_2_, *p*-TSA, toluene; (c) NaH, dipropyl sulfate (**32a**) or (3-bromoprop-1-en-1-yl)cyclohexane (**32b**), DMF; (d) conc. HCl, MeOH; (e) NaH, allyl bromide, DMF; (f) Pd/C, H_2_, THF; (g) TBDMSCl, imidazole, DMF; (h) Propionic anhydride, Et_3_N, toluene; (i) KF, MeOH, DCM

**Scheme 2:**
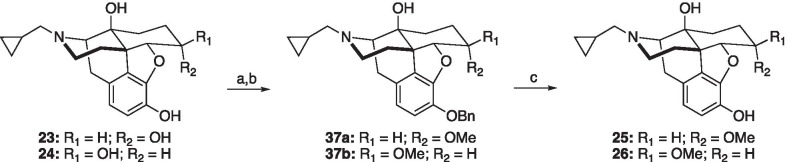
Synthetic route to methyl ether analogues **25** and **26**. *Reagents and conditions* (a) K_2_CO_3_, BnBr, DMF; (b) NaH, MeI, THF; (c) Pd/C, H_2_, THF

**Scheme 3:**
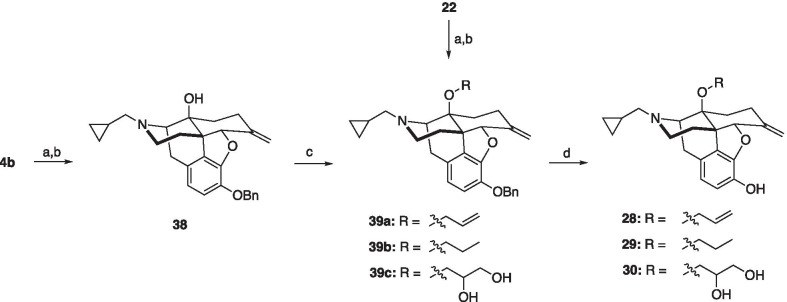
Synthetic route to analogues **28**–**30**. *Reagents and conditions* (a) K_2_CO_3_, BnBr, DMF; (b) *t*-BuOK, MTPPB, THF; (c) NaH, dipropyl sulfate (**39a**) or allyl bromide (**39b**), DMF; (d) conc. HCl, MeOH

Analogues **14**, **16** and **20**–**22** were prepared as shown in Scheme 1. Alkylation of naltrexone (**4b**) with benzyl bromide in the presence of base, followed by protection of the ketone with ethylene glycol, gave the known acetal **31** [29]. 14β-*O*-Alkylation of acetal **31** with dipropyl sulfate or (3-bromoprop-1-en-1-yl)cyclohexane gave ethers **32a** and **32b**, respectively. Global deprotection with HCl of **32a** gave phenol **20** [37]. Reduction of **32b** afforded phenol **33**, which was further deprotected to provide compound **16**. Treatment of acetal **31** with allyl bromide under basic conditions gave allyl ether **34**. Treatment of **34** with HCl gave 14β-*O*-allyl phenol **21** [37]. Dihydroxylation of **34** with AD-mix-α gave diol **35**, which was subsequently deprotected with HCl to afford phenol **22** [38] Analogue **14** [39] was prepared from naltrexone (**4b**) using a sequence of silyl protection, propionic anhydride esterification, and KF deprotection. Methyl ether analogues **25** and **26** were prepared by benzyl protection, methylation, and deprotection of α-naltrexol (**23**) and β-naltrexol (**24**) (Scheme 2). Finally, alkenes **28**–**30** were prepared according to Scheme 3, utilizing standard Wittig alkenylation to insert C6 olefin functionality [30].

### In vitro pharmacology

To validate our fentanyl assay, we examined the effects of naloxone, naltrexone, and the long-lasting opioid antagonist clocinnamox (CCAM) (Table 1). As expected, naloxone, naltrexone, and CCAM antagonized the actions of an EC_90_ dose of fentanyl (2.3 nM). The most potent of these antagonists was naltrexone (IC_50_ = 8.82 ± 1.53 nM). To further assist in the comparison of ligands, we then normalized the level of antagonism of each ligand to naltrexone. Having established our ability to detect μ antagonism, we next chose to evaluate the actions of β-funaltrexamine (β-FNA), an irreversible μ selective antagonist [40]. We found that β-FNA was approximately threefold less potent than naltrexone (IC_50_ = 31.02 nM vs. IC_50_ = 8.82 nM). However, β-FNA produced a decreased level of antagonism compared to naltrexone (I_max_ = 60.0% vs. I_max_ = 102.7%). This was not surprising given the covalent nature of β-FNA and the short pretreatment time (15 min) used. As expected, increasing the pretreatment time to 2 h with β-FNA led to a full level of antagonism (Additional file 1). However, we chose to use a short pretreatment time for our subsequent screening efforts.

**Table 1:** Evaluation of functional activity at MORs using Eurofins DiscoverX HitHunter^®^ cAMP Assay

					
Cmpd	R_1_	R_2_	R_3_	R_4_	LogP^a^	CNS MPO^a^	MOR antagonism IC_50_ (nM)^b,c^ (I_max_)^d^		MOR agonism EC_50_ (nM)^b^ (% efficacy)^e^
Fentanyl					3.82	4.17	NT		0.22 ± 0.04 (103.35 ± 0.28)
Morphine					0.90	4.94	NT		5.92 ± 0.95 (103.00 ± 0.56)
Oxycodone	CH_3_	=O	OH	OH	1.03	5.45	NT		102.12 ± 51.64 (105.78 ± 0.51)
Naloxone (**4a**)	Allyl	=O	OH	OH	1.48	5.31	51.16 ± 13.42 (120.11 ± 4.02)		3.15 ± 0.48 (22.36 ± 5.56)
Naltrexone (**4b**)	CPM	=O	OH	OH	1.27	4.83	8.82 ± 1.53 (102.69 ± 0.94)		2.14 ± 1.20 (29.61 ± 6.40)
β-FNA	CPM	NHCOCH=CHCO_2_Me, H	OH	OH	1.08	3.13	31.02 ± 8.34 (59.98 ± 9.42)		NT
CCAM	CPM	=O	NHCOCH=CH(4-ClC_6_H_4_)	OH	3.99	3.17	29.57 ± 2.98 (137.69 ± 5.98)		> 10,000
**7**	CPM	=O	OH	OCH_3_	1.82	5.09	> 10,000		103.02 ± 50.94 (45.87 ± 8.62)
**8**	CPM	=O	OH	H	1.97	5.03	> 10,000		138.63 ± 55.18 (27.42 ± 5.41)
**9**	CPM	=O	H	OH	2.07	5.03	1.51 ± 0.68 (50.23 ± 4.32)		0.27 ± 0.06 (71.33 ± 4.16)
**10**	CPM	=O	OCOCH=CH(4-ClC_6_H_4_)	OH	4.95	2.54	> 10,000		386.90 ± 115.16 (68.74 ± 8.13)
**11**	CPM	=O	OCOCH=CH(4-CH_3_C_6_H_4_)	OH	4.85	2.74	> 10,000		168.35 ± 31.99 (68.88 ± 11.09)
**12**	CPM	=O	OCOCH=CHC_6_H_5_	OH	4.34	3.35	> 10,000		6.60 ± 0.50 (89.14 ± 3.45)
**13**	CPM	=O	OCOCH_2_CH_2_C_6_H_5_	OH	4.05	3.64	> 10,000		16.68 ± 1.60 (78.98 ± 3.84)
**14**	CPM	=O	OCOCH_2_CH_3_	OH	2.47	5.12	4.22 ± 1.13 (115.18 ± 2.54)		4.98 ± 2.16 (19.63 ± 5.24)
**15**	CPM	=O	OCH_2_CH_2_CH_2_Ph	OH	4.34	3.44	> 10,000		0.38 ± 0.11 (101.01 ± 1.86)
**16**	CPM	=O	OCH_2_CH_2_CH_2_Cy	OH	4.81	2.93	> 10,000		1.89 ± 0.61 (98.55 ± 3.8)
**17**	CPM	=O	OCH_3_	OH	1.89	5.12	13.26 ± 6.29 (71.74 ± 12.10)		2.99 ± 1.56 (25.31 ± 2.76)
**18**	CPM	=O	OCH_2_Ph	OH	3.61	4.31	38.95 ± 15.76 (102.54 ± 26.42)		15.38 ± 3.52 (19.98 ± 1.1)
**19**	CPM	=O	OCH_2_CH_3_	OH	2.23	5.04	3.97 ± 1.65 (89.24 ± 5.87)		0.44 ± 0.22 (49.66 ± 8.51)
**20**	CPM	=O	OCH_2_CH_2_CH_3_	OH	2.76	4.95	2.55 ± 0.94 (94.95 ± 15.00)		6.57 ± 3.03 (38.72 ± 8.31)
**21**	CPM	=O	OCH_2_CH=CH_2_	OH	2.60	4.95	2.58 ± 0.75 (96.84 ± 5.49)		0.72 ± 0.33 (37.41 ± 8.47)
**22**	CPM	=O	(*R*)-OCH_2_CHOHCH_2_OH	OH	0.60	3.79	6.49 ± 1.49 (149.78 ± 15.31)		> 10,000
**5**	CPM	=CH_2_	OH	OH	1.95	4.72	2.13 ± 0.20 (128.85 ± 7.09)		> 10,000
**23**	CPM	α-OH, H	OH	OH	0.84	4.43	20.83 ± 9.92 (69.00 ± 9.72)		2.22 ± 1.04 (49.31 ± 11.57)
**24**	CPM	β-OH, H	OH	OH	0.84	4.43	5.85 ± 2.03 (110.63 ± 6.30)		0.94 ± 0.35 (26.88 ± 4.74)
**25**	CPM	α-OCH_3_, H	OH	OH	1.49	4.77	18.59 ± 5.87 (85.54 ± 5.14)		1.86 ± 0.39 (58.16 ± 4.45)
**26**	CPM	β-OCH_3_, H	OH	OH	1.49	4.77	4.52 ± 1.62 (99.67 ± 7.40)		0.88 ± 0.43 (39.02 ± 5.62)
**27**	CPM	H_2_	OH	OH	1.86	4.71	8.83 ± 3.52 (121.83 ± 5.64)		0.88 ± 0.43 (31.69 ± 3.69)
**28**	CPM	=CH_2_	OCH_2_CH=CH_2_	OH	3.80	4.71	2.03 ± 0.96 (100.42 ± 18.87)		0.34 ± 0.15 (38.30 ± 7.02)
**29**	CPM	=CH_2_	OCH_2_CH_2_CH_3_	OH	3.95	4.55	2.06 ± 0.53 (86.70 ± 5.46)		1.21 ± 0.44 (16.99 ± 3.50)
**30**	CPM	=CH_2_	(*R*)-OCH_2_CHOHCH_2_OH	OH	1.75	4.78	3.58 ± 1.28 (115.90 ± 8.44)		> 10,000

^a^Calculated using CDD Vault

^b^Values are expressed as the mean ± SEM of at least three independent measurements

^c^Antagonist potency (IC_50_) determined versus EC_90_ of fentanyl

^d^Degree of antagonism (I_max_) normalized to **4b**

^e^Agonist efficacy expressed as percent stimulation

With our series of epoxymorphinans in hand, we sought to explore their activity in our fentanyl antagonism assay. Initially, we sought to confirm the importance of the C3-phenolic group in naltrexone. As expected, conversion of the phenol to the methyl ether **7** [22] or its removal (**8**) [23] resulted in a complete loss of antagonist activity (IC_50_ > 10,000 nM). These results suggested that the C3-phenol was a key functional group, and it was necessary to retain it in future analogues.

We next sought to understand if modification at the C14β-hydroxyl position could provide enhanced opioid antagonist activity. Replacement of the C14β-hydroxy group with a hydrogen (**9**) [24] resulted in a sixfold increase in antagonist potency compared to naltrexone (IC_50_ = 1.51 nM vs. IC_50_ = 8.82 nM). However, this modification resulted a weaker level of fentanyl antagonism (I_max_ = 50.2% vs. I_max_ = 102.7%). These results suggested that the potency of naltrexone could be enhanced through additional structural modification, but the 14β-hydroxy position was important to maintaining a full level of antagonism.

Interestingly, replacement of the 14β-amide in CCAM with a 14β-ester (**10**) resulted in a complete loss of antagonist activity (IC_50_ = 29.57 nM vs. IC_50_ > 10,000 nM) [25, 41]. Modification of the aromatic substituent in **10** (**11**, **12**) [25] and replacement of the ester with an ether (**15**) resulted no enhancement of antagonist activity, rather an increase in agonist activity. Despite its high lipophilicity, ether **15** [27] was found to be inactive as an antagonist and to be an extremely potent MOR agonist (EC_50_ = 0.38 nM) [27]. Further increasing lipophilicity by replacement of the phenyl ring in **15** with a cyclohexyl group (**16**) also had no effect on antagonizing fentanyl but did decrease MOR agonist activity (EC_50_ = 1.89 nM).

Removal of the alkene of **12** (**13**) [26] decreased agonist activity (EC_50_ = 16.68 nM) with no observable level of antagonism. Thinking that the phenyl ring might be responsible for the weak level of antagonist activity, we replaced it with a hydrogen atom (**14**). To our delight, this modification resulted in an increase in antagonist activity (IC_50_ = 4.22 nM). We were excited to see that this change also resulted in a high degree of antagonism (I_max_ = 115.2%). In agreement with a previous report, we found that alkylation of the 14β-hydroxy group of naltrexone with a methyl group (**17**) [28] and ethyl group (**19**) [28] were well tolerated (**17**: IC_50_ = 13.26 nM and **19**: IC_50_ = 3.97 nM, respectively). In contrast to previous literature, however, we found that a benzyl group (**18**) [29] decreased MOR antagonist activity approximately fourfold compared to naltrexone (IC_50_ = 38.95 nM vs. IC_50_ = 8.82 nM) [29]. Homologation of the ethyl group to propyl (**20**) resulted in an approximately threefold increase in activity compared to naltrexone (IC_50_ = 2.55 nM vs. IC_50_ = 8.82 nM). Conversion of the propyl group to an allyl group (**21**) was well tolerated (IC_50_ = 2.58 nM). Interestingly, dihydroxylation of the allyl group in **21** (**22**) only slightly decreased activity (IC_50_ = 6.49 nM vs IC_50_ = 2.58 nM) despite significantly decreasing logP (**22**: logP = 0.60 vs. **21**: logP = 2.60). This further suggests that lipophilicity is not an essential characteristic in antagonizing fentanyl.

We next chose to evaluate the role of the C6-keto group in naltrexone. Previous structure–activity relationships suggested that the replacement of the C-6 carbonyl in naltrexone by a methylene group would increase opioid antagonism [30]. As expected, nalmefene (**5**) was found to be approximately fourfold more potent than naltrexone (IC_50_ = 2.13 vs. IC_50_ = 8.82 nM). Next, we explored the reduction of the C6-keto group to 6α-naltrexol (**23**) [30] and 6β-naltrexol (**24**) [31]. As expected, there was a clear stereochemical preference [31, 42]. 6β-Naltrexol (**24**) was found to be approximately fourfold more potent than 6α-naltrexol (**23**) (IC_50_ = 5.85 nM vs. IC_50_ = 20.83 nM). In addition, 6β-naltrexol (**24**) was found to be slightly more potent than naltrexone (IC_50_ = 5.85 nM vs. IC_50_ = 8.82 nM). This later result was not surprising given that **24** is an active metabolite of naltrexone [43]. Interestingly, methylation of the 6α-alcohol of **23** (**25**) or the 6β-alcohol of **24** (**26**) resulted in the retention of antagonist activity (IC_50_ = 18.59 nM vs. IC_50_ = 20.83 nM and IC_50_ = 4.52 nM vs. IC_50_ = 5.85 nM, respectively). Finally, we explored the removal of the C6-keto of naltrexone (**27**) [32]. This modification was also well tolerated (IC_50_ = 8.83 nM vs. IC_50_ = 8.82 nM).

Having explored the importance of the C3-phenol, the 14β-hydroxyl group, and the C6-keto group, we investigated if combinations of structural changes were additive. Initially, we prepared alkene **28** based on the availability of starting material and tolerance of a 14β-*O*-allyl group. The combination was well-tolerated and a potent antagonist was identified (IC_50_ = 2.03 nM). Given this positive result, we also synthesized alkene **29** due to the high activity of **20** (IC_50_ = 2.55 nM). As expected, **29** was found to be a highly potent antagonist (IC_50_ = 2.06 nM). Introduction of the 6-methylene to **22** (**30**) also increased antagonist activity (IC_50_ = 3.58 nM vs. IC_50_ = 6.49 nM), however, with a reduction in the degree of antagonism (I_max_ = 115.9% vs I_max_ = 149.8%).

Additional Schild analyses were conducted on naltrexone, **29**, and **30**, using a full dose–response curve of fentanyl in the absence or presence of three concentrations of test compounds. The Schild slopes of all three compounds were determined to not significantly deviate from 1, indicating competitive antagonism. The pA_2_ values (the concentrations of antagonist required to have a twofold increase in the concentration of agonist to produce a selected effect) of analogues **29** and **30** were found to be 10.01 and 9.77, respectively, in contrast to 9.47 for the parent compound naltrexone, which suggested a greater potency resulting from our design (Table 2). In addition, the equilibrium dissociation constant (K_e_) values were also calculated. Analogues **29** and **30** were found to have K_e_ values of 0.103 nM and 0.159 nM, respectively, in contrast to 0.300 nM for naltrexone. These findings are consistent with the aforementioned IC_50_ data and support the utility of our in vitro assay in identifying potent MOR antagonists.

**Table 2:** Schild analysis and *K*_e_ values of antagonism of test compounds against fentanyl to MORs by cAMP functional assay

Cmpd	Slope ± SEM	pA_2_ ± SEM	*K*_e_ (nM) ± SEM
**4b**	1.12 ± 0.14	9.47 ± 0.13	0.300 ± 0.097
**29**	1.04 ± 0.12	10.01 ± 0.18	0.103 ± 0.030
**30**	1.22 ± 0.14	9.77 ± 0.14	0.159 ± 0.047

Values are expressed as the mean ± SEM of at least three independent measurements

Knowing the promiscuous nature of naltrexone, several analogues possessing low nanomolar MOR antagonist activity (**14**, **20**–**22**, **28**–**30**) were chosen for additional screening at KORs and DORs (Table 3). Similar to naltrexone, most of these compounds exhibit partial KOR agonist activity, the most potent of which being the 14β-*O*-allyl analogues **21** (EC_50_ = 0.12 nM) and **28** (EC_50_ = 0.14 nM). Interestingly, diol analogues **22** and **30** possess no agonist activity at KORs (EC_50_ > 10,000). This is similar to their actions at MORs (EC_50_ > 10,000). In contrast, activity of this series of compounds at DORs appears to be more varied. While naltrexone exhibits weak DOR antagonism, the majority of these compounds display partial DOR agonism, the most potent of which being analogue **29** (EC_50_ = 0.19 nM). Analogue **22** remains an exception, exhibiting no DOR agonist activity.

**Table 3:** Evaluation of functional activity at KORs and DORs using Eurofins DiscoverX HitHunter^®^ cAMP Assay

Cmpd	KOR antagonism IC_50_ (nM)^a,b^ (I_max_)^c^	KOR agonism EC_50_ (nM)^a^ (% efficacy)^d^	DOR antagonism IC_50_ (nM)^a,b^ (I_max_)^c^	DOR agonism EC_50_ (nM)^a^ (% efficacy)^d^
**4b**	5.53 ± 1.02 (41.31 ± 6.83)	0.64 ± 0.32 (56.46 ± 7.15)	177.16 ± 48.90 (99.57 ± 1.93)	> 10,000
**14**	8.15 ± 1.10 (49.27 ± 11.54)	1.56 ± 0.69 (58.71 ± 5.77)	> 10,000	2.09 ± 0.80 (47.42 ± 2.58)
**20**	8.70 ± 1.89 (46.68 ± 2.95)	0.67 ± 0.39 (39.11 ± 4.70)	4.06 ± 2.36 (29.48 ± 5.94)	1.04 ± 0.35 (48.05 ± 1.87)
**21**	10.68 ± 4.48 (62.72 ± 18.41)	0.12 ± 0.03 (41.17 ± 8.81)	> 10,000	2.28 ± 0.72 (45.47 ± 2.38)
**22**	33.74 ± 3.77 (84.97 ± 6.39)	> 10,000	368.31 ± 136.17 (89.22 ± 4.30)	> 10,000
**28**	8.54 ± 3.54 (47.32 ± 12.19)	0.14 ± 0.04 (44.09 ± 8.43)	> 10,000	0.72 ± 0.22 (48.92 ± 3.66)
**29**	4.49 ± 2.02 (46.84 ± 16.34)	0.43 ± 0.18 (59.08 ± 8.54)	> 10,000	0.19 ± 0.09 (54.96 ± 3.44)
**30**	8.75 ± 2.05 (89.12 ± 0.69)	> 10,000	48.58 ± 24.06 (49.76 ± 1.50)	0.86 ± 0.33 (32.98 ± 3.21)
*nor*-BNI	2.10 ± 0.64 (98.54 ± 2.47)	NT	NT	NT
Salvinorin A	NT	0.026 ± 0.005 (98.66 ± 0.85)	NT	NT
U50488H		0.18 ± 0.06 (98.99 ± 0.83)		
Naltrindole	NT	NT	0.51 ± 0.17 (100.14 ± 6.20)	NT
SNC-80	NT	NT	NT	1.27 ± 0.22 (76.33 ± 1.38)

^a^Values are expressed as the mean ± SEM of at least three independent measurements

^b^Antagonist potency (IC_50_) determined versus EC_90_ of U50,488H for KORs and EC_50_ of SNC-80 for DORs

^c^Degree of antagonism (I_max_) normalized to *nor*-BNI for KORs and **4b** for DORs

^d^Agonist efficacy expressed as percent stimulation

### Pharmacokinetics

**Fig. 2:**
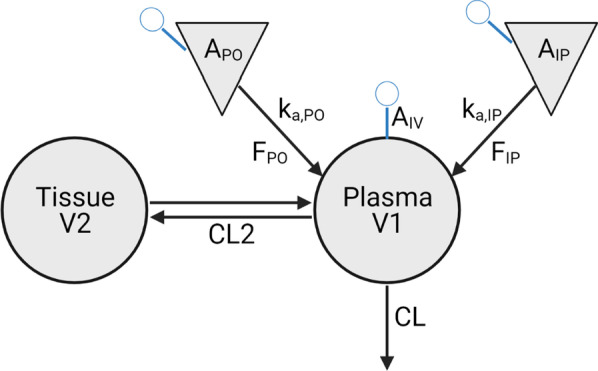
Schematic depiction of the two-compartment pharmacokinetic model fitted to the **29** plasma concentrations. The intravenous dose was administered in the plasma compartment (A_IV_). Oral and intraperitoneal doses (A_PO_ and A_PI_, respectively) were administered in absorption compartments

**Fig. 3:**
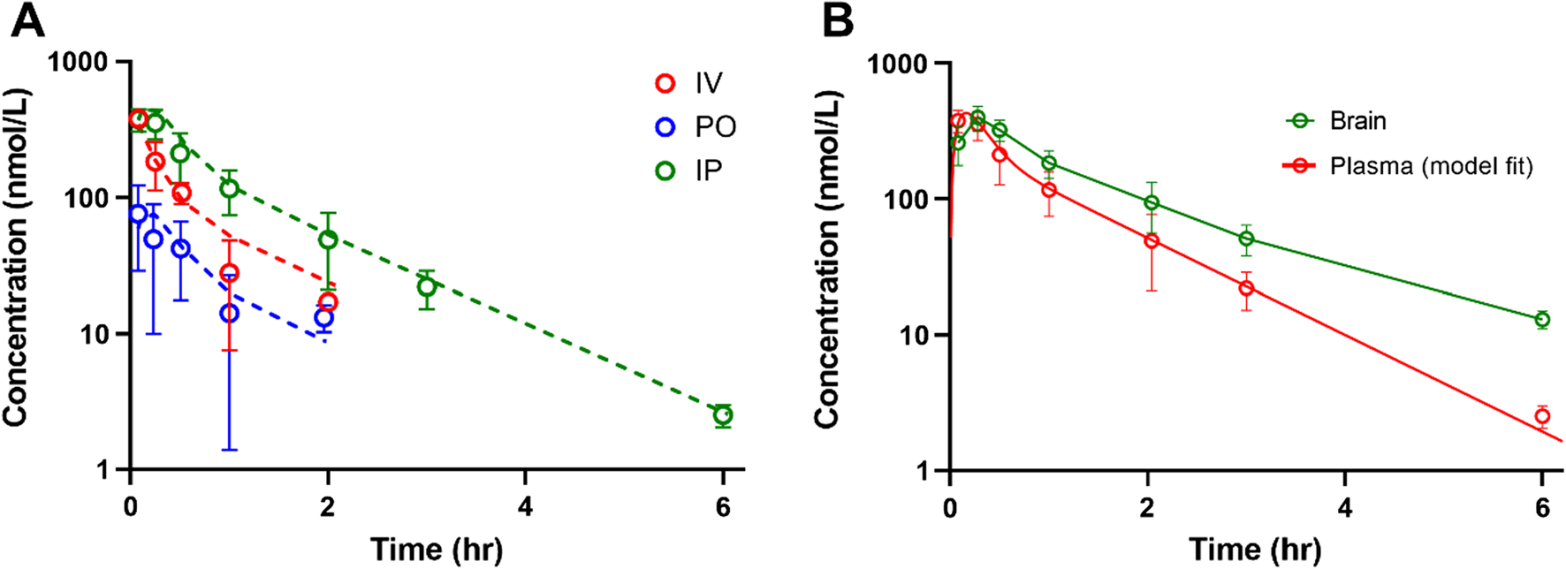
Pharmacokinetic data following **29** administration by multiple routes. Plasma concentrations by IV, PO, and IP routes (**A**) and comparative plasma and brain concentrations following IP dosing (**B**)

The pharmacokinetics of **29** were evaluated using C57BL/6J mice. Animals were administered 1 mg/kg, 5 mg/kg, and 2 mg/kg via IV, PO, and IP dosing routes. Plasma concentrations were above the lower quantitation limit for 2 h following IV and PO dosing, while IP dosing resulted in measurable concentrations for at least 6 h. Plasma concentrations were fitted with a 2-compartment model to capture the biphasic elimination of **29** (Fig. 2). The model estimated PK parameters are listed in Table 4. The oral bioavailability of **29** was approximately 6.6% while the bioavailability following IP administration was estimated to be near 100%.** 29** was rapidly absorbed and the T_max_ occurs within the first 10 min of PO and IP administration (Fig. 3A, Table 5). The plasma half-life of **29** was estimated to ~ 0.6–1 h. Assessment of brain biodistribution demonstrated ample **29** distribution into the brain with a T_max_ of approximately 20 min. Subsequently, brain concentrations surpass plasma concentrations and the apparent terminal half-life in the brain is ~ 80 min as compared to 55 min in plasma (Fig. 3B, Table 5). This high partition into brain suggests that there is ample free concentration of **29** in plasma and that the compound binds more preferentially to brain tissue components than it does to plasma proteins. Attempts to model the brain penetration along with the plasma concentrations were not successful, but based on the difference in apparent half-life, best fitting would likely be achieved using a saturable redistribution model. This may be related to **29** tissue binding and prolonged partition into the brain tissue. The estimated partition coefficient, K_p,brain_, using NCA estimates of AUC (Fig. 3B, Table 5) was 1.6.

**Table 4:** Pharmacokinetic parameter estimates of **29** plasma concentrations obtained using the PK model depicted in Fig. 2

Parameter	Estimate	Units	Standard error	CV%
V	5.36	L/kg	2.91	54.2
CL	12.13	L/h/kg	2.02	16.7
K_a,po_	7.91	1/h	6.18	78.1
V2	6.07	L/kg	1.55	25.5
CL2	10.44	L/h/kg	4.25	40.7
F_po_	6.6	%	1.5	23.3
K_a,ip_	7.53	1/h	4.23	56.2
F_IP_	99.5	%	17.1	17.2

**Table 5:** Descriptive pharmacokinetic parameters of **29** exposure

Dosing route/dose	AUC (nM-h)	T_max_ (h)	Apparent half-life, t_½_ (h)
IV, 2 mg/kg	207	0.08	0.58
PO, 5 mg/kg	64	0.1	0.99
IP, 2 mg/kg Plasma	397	0.12	0.94
IP, 2 mg/kg Brain	634	0.32	1.34

### Antagonism by 29 of oxycodone-induced antinociception

**Fig. 4:**
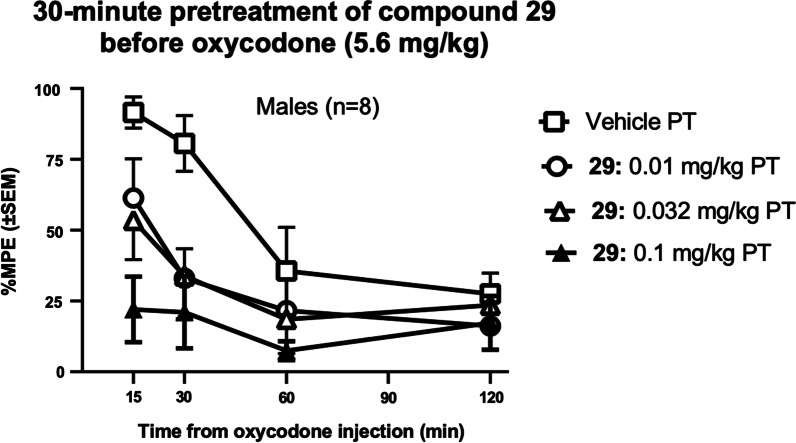
Dose-dependence of antagonist effects of compound **29** (0.01–0.1 mg/kg, IP), administered 30 min before oxycodone (5.6 mg/kg), in the thermal antinociception assay. X-axis: Time in minutes after oxycodone administration. Y-axis: %Maximum possible effect (%MPE)

As expected based on prior studies, oxycodone (5.6 mg/kg), 30 min after vehicle pretreatment, resulted in a near-maximal antinociceptive effect (Fig. 4). The peak effects of oxycodone were observed 15 min after injection. Different doses of compound **29** were administered as a 30-min pretreatment to oxycodone (5.6 mg/kg). Compound **29** (0.01–0.1 mg/kg) caused a dose-dependent prevention of oxycodone-induced antinociception. The larger pretreatment dose of **29** (0.1 mg/kg) caused a complete prevention of oxycodone-induced effects. A 2-Way repeated measures ANOVA (**29** dose × time post-oxycodone) yielded a significant main effect of **29** dose (F (3, 21) = 7.263; p = 0.0016), a main effect of time post-oxycodone (F (3, 21) = 11.72; p = 0.0001), and their interaction F (9, 63) = 2.126; p = 0.04.

**Fig. 5:**
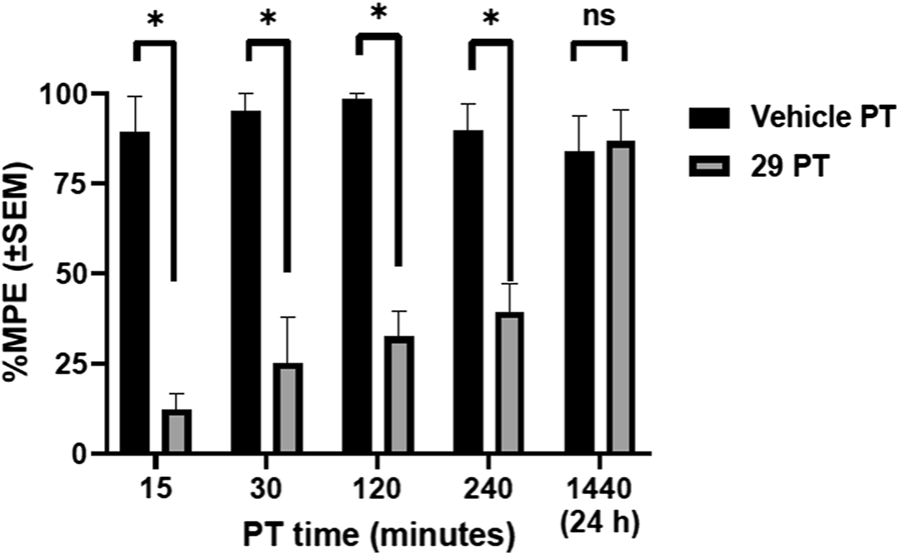
Time course of antagonist effects of compound **29** (0.1 mg/kg, IP), administered at different pretreatment (PT) before oxycodone (5.6 mg/kg), in the thermal antinociception assay (n = 7–8). X-axis: PT time for **29** before oxycodone injection. Y-axis: %Maximum possible effect (%MPE). Data are shown at the 15-min timepoint after oxycodone (i.e., its peak effect). Stars represent significance of post-hoc Sidak tests

We then examined the time course of antagonism by **29** of oxycodone-induced antinociception (Fig. 5). The largest dose of **29** (0.1 mg/kg, or vehicle) was administered at different times (15, 30, 120, 240 min and 24 h) prior to oxycodone (5.6 kg). At each pretreatment time other than 24 h, **29** was able to significantly prevent the peak antinociceptive effects of oxycodone (i.e. measured 15 min after oxycodone injection). A 2-Way mixed effects ANOVA (**29** or vehicle PT, by PT time) was significant for the main effect of **29** or vehicle PT (F (1, 32) = 116.1; p < 0.0001). There was also a main effect of PT time (F (4, 32) = 4.25; p = 0.007) and an interaction between PT injection and time (F(4, 32) = 9.37; p < 0.0001). Sidak’s post hoc tests show that **29** PT differed from vehicle PT at 15, 30, 120 and 240 min, but not 24 h prior to oxycodone.

### Antagonism by 29 of locomotor deficits caused by the KOR agonist U50,488

**Fig. 6:**
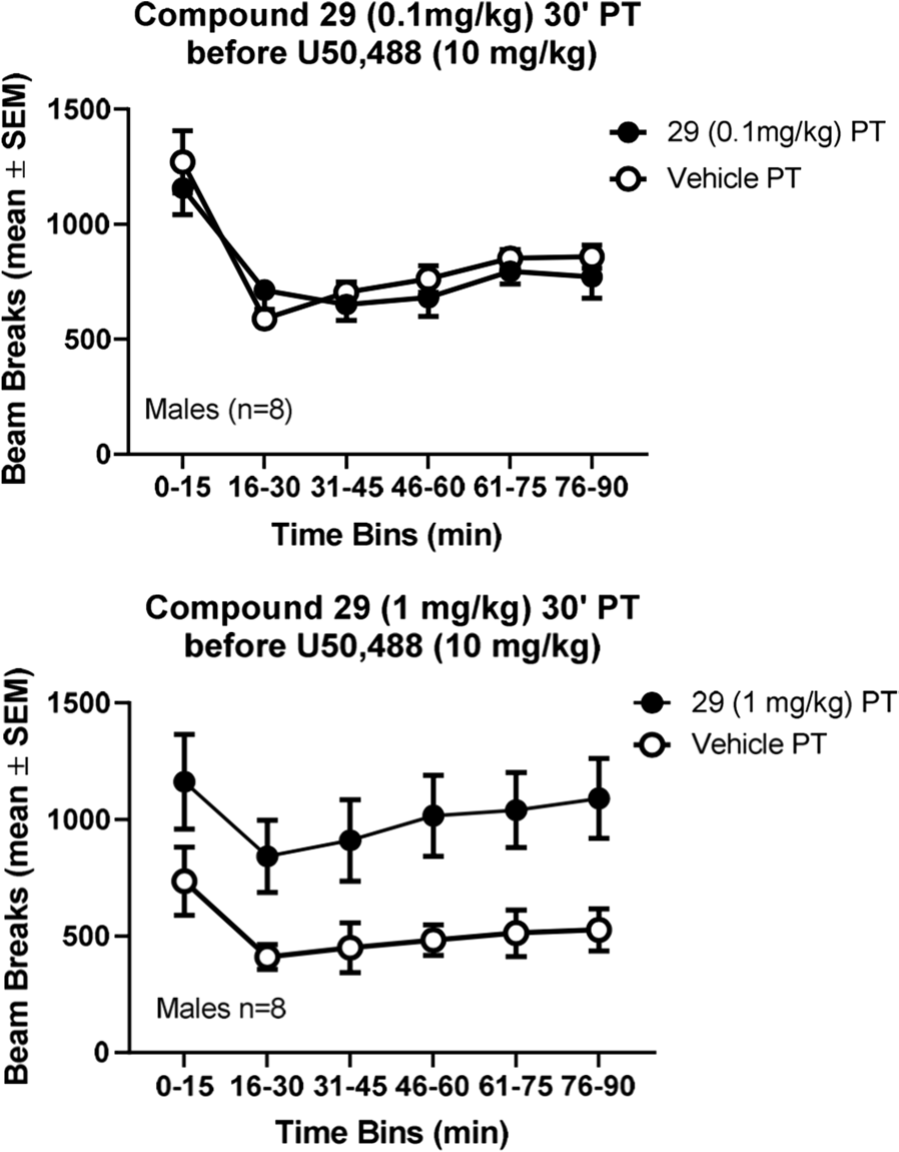
Dose-dependence of antagonist effects of compound **29** (0.1 and 1.0 mg/kg), administered 30 min before U50,488 (10 mg/kg), in the locomotor activity assay. X-axis: Time bins in minutes after U50,488. Y-axis: Photocell beam breaks in the locomotor activity cage

These studies were designed to examine the in vivo selectivity of **29** as a MOR-antagonist, as opposed to a KOR-antagonist. As expected based on recent studies [36], U50,488 (10 mg/kg; 30 min after vehicle PT) caused a robust decrease in locomotor activity over 90 min (Fig. 6). PT with **29** (0.1 mg/kg, compared to vehicle) did not cause any apparent blockade of the locomotor effects of U50,488 (10 mg/kg). Thus, a 2-way repeated measures ANOVA (**29** or vehicle PT by time bin) was not significant for the main effect of **29** or vehicle PT, or its interaction with time bin (not shown). There was a main effect of time bin (F (5, 35) = 26.02; p < 0.0001). However, pretreatment with a tenfold larger dose of **29** (1 mg/kg, compared to vehicle) did block the locomotor depressant effects of U50,488 (10 mg/kg). Thus, a 2-way repeated measures ANOVA (**29** or vehicle PT by time bin) was significant for the main effect of **29** or vehicle PT (F (1, 7) = 6.14; p = 0.042); there was no significant interaction between **29** PT and time bin (not shown). There was a main effect of time bin (F (5, 35) = 11.14; p < 0.0001). In separate control studies, this larger dose of **29** (1 mg/kg) alone did not cause significant effects on locomotor activity, compared to a vehicle injection (not shown).

## Discussion

Clinical studies and case reports have indicated that overdoses from fentanyl (mediated by respiratory depression) frequently require multiple naloxone administrations due to the shorter duration of action of naloxone than that of fentanyl [9]. Additionally, fentanyl can result in chest wall rigidity, which further interferes with breathing and exacerbates the risk of mortality [44]. Taken together, this suggests that there is a need for the development of new fentanyl overdose treatments.

To address this need, we initially sought to develop an in vitro functional assay capable of identifying effective synthetic opioid rescue agents. Given that the rescue effects of naloxone are likely due to its antagonism at MORs, we decided to adapt a commercially available Eurofins DiscoverX HitHunter^®^ cAMP Assay [33, 45, 46]. First, we elected to evaluate the ability of test ligands to antagonize an EC_90_ concentration of fentanyl. Our rationale was based on attempting to find compounds that would provide maximal protection against fentanyl’s effects. Second, we chose to use a relatively short incubation time (15 min) to help identify rapidly acting synthetic opioid rescue agents. To more optimally assist in an overdose or chemical attack situation with fentanyl or other synthetic opioid, a rescue agent is needed that has a rapid onset of action. While this property would ultimately need to be characterized in vivo, using a short incubation time might assist in identifying such agents in a more rapid manner. Third, this assay uses no radioactivity and is more environmentally friendly than previously described radioactive methods using the [^35^S]GTPγS assay [47, 48]. Finally, we envisioned that the assay results would be obtained with high throughput to help direct our synthetic efforts.

With a viable assay in hand, we focused on synthesizing and evaluating analogues of naltrexone. This was based on previous reports which showed that naltrexone had enhanced potency compared to naloxone and is available from commercial sources [30]. Naltrexone has previously served as the starting point for the development of selective opioid receptor probes naltrindole and nor-binaltorphimine (*nor*-BNI) [49–51]. We assume that the rescue effects of naloxone is likely due to antagonizing the action of **1** at MORs [52]. However, since naloxone is a non-selective antagonist at opioid receptors [53], actions at δ opioid receptors (DORs) and κ opioid receptors (KORs) were not disregarded.

Having selected a starting point for chemical synthesis, we prepared a series of analogues modified in three positions: (1) the C3-phenol; (2) the 14β-hydroxyl group; and (3) the C6-keto group. These positions were selected due to synthetic tractability and potential to alter the pharmacokinetic properties. In addition, several analogues of naltrexone modified at these positions have been prepared previously. The structure–activity relationships from these previous investigations were expected to provide valuable insights in the design of an enhanced synthetic opioid rescue agent [53, 54].

According to previous literature, the C3-phenol is an important feature of the morphinan pharmacophore, participating in a critical H-bond interaction at opioid receptors [55]. To verify this hypothesis, analogues **7** and **8** (possessing C3-OMe and C3-H, respectively) were synthesized and evaluated in vitro. As expected, both analogues resulted in a complete loss of antagonist activity (IC_50_ > 10,000 nM), supporting the hypothesis of the key phenol interaction. Further analogues were designed to retain this feature.

Several analogues of naltrexone possessing various modifications to the 14β-hydroxyl group have previously been reported [25, 27, 56]. Of note, CCAM displays increasingly potent antagonist activity with a longer duration of action compared to naltrexone. Other 14β analogues, however, have resulted in a significant loss in antagonist activity or even a complete “switch” from antagonist to agonist activity. Taken together, this data suggests that modification of the 14β-hydroxyl group has the ability to alter the potency and pharmacokinetic properties of naltrexone.

After making an initial series of MOR agonists (**10**–**13, 15, 16**), truncation of the phenyl ring at the 14β position led to a series of analogues possessing potent MOR antagonist activity (**14**, **19** – **22**). Of these, the 14β-*O*-propyl (**20**) and 14β-*O*-allyl (**21**) analogues displayed the lowest IC_50_ values (2.55 nM and 2.58 nM, respectively), providing the identification of our first lead compounds.

Several modifications to the C6-ketone have also been reported to alter the activity of naltrexone. Of particular interest, substitution to the C6-alkene provides nalmefene (**5**), possessing a fourfold increase in potency compared to naltrexone (IC_50_ = 2.13 nM vs IC_50_ = 8.82 nM). This favorable trend led to the introduction of the C6-alkene onto the previously discovered antagonists **20** and **21**, providing the potent analogues **29** and **28**, respectively. These analogues are believed to be among the most potent MOR antagonists known to date (IC_50_ = 2.06 nM and IC_50_ = 2.03 nM).

As expected, analogues **28** and **29** exhibit partial KOR agonist activity, similar to that of naltrexone. Their activity at the DOR, however, differs from naltrexone as they display potent partial agonism and no DOR antagonism. This change is activity is not expected to negatively impact the effectiveness, nor contribute harmful side effects, of a fentanyl overdose rescue agent.

Potent analogue **29** was chosen to undergo further PK analysis to assess its duration of action and brain distribution. We found that **29** was rapidly absorbed with a near 100% bioavailability following IP administration. Furthermore, brain concentrations of **29** surpassed plasma concentrations, suggesting ample and prolonged partition into the brain tissue. However, **29** possesses a relatively short plasma half-life (0.9 h) with an apparent terminal half-life of ~ 80 min in the brain. In comparison, the terminal half-life of fentanyl is ~ 220 min, nearly 3× that of **29**.

To determine the in vivo on-target effectiveness of **29**, antagonism of oxycodone-induced antinociception was studied using the traditional hot plate assay [57]. The hot plate assay is a widely used preclinical test of supra-spinal analgesic efficacy, possessing a high predictive value for drugs targeting MORs [58]. Before tackling the more complex in vivo pharmacology of fentanyl and fentanyl analogues [44], antagonism of oxycodone-induced antinociception was selected as an initial proof of concept to verify the in vivo actions of our new opioid rescue agents. Results from these studies show that **29** is dose-dependently (0.01–0.1 mg/kg) and fully effective in preventing antinociception caused by the frequently abused MOR agonist, oxycodone. In these studies, **29** also showed relatively fast onset of antagonist action after IP injection (within 15 min) and a duration of action of at least 240 min, but less than 24 h. This profile is desirable in principle, as sufficient duration of action is important to prevent the effects of high potency abused MOR agonists which are currently causing considerable morbidity [59]. Therefore, the duration of MOR-antagonist action of **29** appears to be more extended than that indicated by PK analyses above.

Other in vivo studies also show that **29** has relative selectivity as an antagonist of MOR- over KOR-mediated effects. Specifically, the dose of **29** that is fully effective in preventing oxycodone-induced antinociception (0.1 mg/kg) was ineffective against locomotor deficits caused by the KOR agonist U50,488. However, a tenfold greater dose of **29** (1 mg/kg) was able to prevent locomotor deficits caused by this KOR agonist. Overall, this shows that doses of **29** could be titrated in vivo to block only MOR mediated effects, as opposed to both MOR and KOR mediated effects. This profile could be examined in further translational models in the future.

## Conclusions

To combat the ever-increasing rate of death by opioid overdose (due to recreational use and/or chemical warfare situations), this research aims at identifying a synthetic opioid rescue agent superior to naloxone. Our studies began with the development of an in vitro functional assay capable of identifying ligands with the ability to antagonize an EC_90_ concentration of fentanyl. This assay, with its high fentanyl challenge dose and short pretreatment time, better represents an overdose situation, increasing the likelihood of identifying effective rescue agents. Indeed, following the design and synthesis of novel naltrexone analogues, in vitro analysis using our modified functional assay led to the identification of a series of potent MOR antagonists, including the highly potent analogue **29**. Further in vivo studies highlight the quick onset of action and ample brain distribution of this compound**.**

Even though the results from the hot plate assay suggest a relatively long duration of action, the PK analysis of **29** reveals its terminal half-life to only be ~ 80 min, roughly one-third that of fentanyl’s terminal half-life. This difference in half-life (and possibly duration of action) poses a potential problem, as the chance of renarcotization remains possible. However, the duration of MOR antagonist action of **29** observed in vivo herein is at least 240 min, giving rise to the possibility that the pharmacodynamic duration of this compound is longer than that expected based on systemic PK data. In order to avoid renarcotization and the need for repeated administration, the duration of action of an improved rescue agent is desired to be greater than that of fentanyl. Therefore, future studies are currently underway aimed at modifying the structure of **29** to increase its half-life and duration of action, while maintaining its potency profile. Such an agent has the potential to become a clinical candidate for the reversal of synthetic opioid overdose.

## Supplementary Information

**Additional file 1.** File includes synthetic experimental details, 1H NMR spectra, 13C NMR spectra, and HPLC chromatograms of 14, 16, 20–22, and 25, 26 and 28–30.

## Data Availability

Additional data is available. The data that support the findings of this study are available from the corresponding author upon reasonable request.

## Abbreviations

%MPE: %Maximum possible effect
AUC: Area under the concentration–time curve
cAMP: Cyclic adenosine monophosphate
CHO: Chinese hamster ovary
CNS: Central nervous system
DOR: Delta opioid receptor
ED_50_: Median effective dose
IP: Intraperitoneal
IV: Intravenous
KOR: Kappa opioid receptor
MOR: Mu opioid receptor
NCA: Non-compartmental analysis
OIRD: Opioid-induced respiratory depression
PO: Oral
PT: Pre-treatment
