# Supplementary material for: Design, synthesis, and preliminary evaluation of a potential synthetic opioid rescue agent
Source: J Biomed Sci. 2021 Sep 9;28:62. doi: 10.1186/s12929-021-00758-y (PMC8427951; doi:10.1186/s12929-021-00758-y)
Supplement: Supplementary file 1 — Additional file 1. File includes synthetic experimental details, 1H NMR spectra, 13C NMR spectra, and HPLC chromatograms of 14, 16, 20–22, and 25, 26 and 28–30. [file 12929_2021_758_MOESM1_ESM.pdf]

Additional File 1

Design, Synthesis, and Preliminary Evaluation of a Potential Synthetic Opioid Rescue Agent

Sidnee L. Hedrick, Dan Luo, Sophia Kaska, Kumar Kulldeep Niloy, Karen Jackson, Rupam Sarma, Jamie Horn, Caroline Baynard, Markos Leggas, Eduardo R. Butelman, Mary Jeanne Kreek, and Thomas E. Prisinzano

Department of Pharmaceutical Sciences, College of Pharmacy, University of Kentucky, Lexington, KY 40536 (S.L.H., D.L., S.K., K.N., K.J., R. S., J.H., M.L., T.E.P.)
Center for Pharmaceutical Research and Innovation, College of Pharmacy, University of Kentucky, Lexington, KY 40536 (K.J., R.S., J.H., M.L., T.E.P.)
Laboratory on the Biology of Addictive Diseases, The Rockefeller University, New York, NY 10065 (C.B., E.R.B., M.J.K.)

Table of Contents

General synthetic information	S2
General procedure of oxalate salt formation	S2
Preparation of **36**	S2
Preparation of **14**	S2
Preparation of **32b**	S2
Preparation of **16**	S3
Preparation of **32a**	S3
Preparation of **20**	S4
Preparation of **34**	S4
Preparation of **21**	S4
Preparation of **35**	S5
Preparation of **22**	S5
Preparation of **40a**	S5
Preparation of **37a**	S5
Preparation of **25**	S6
Preparation of **40b**	S6
Preparation of **37b**	S6
Preparation of **26**	S7
Preparation of **38**	S7
Preparation of **39a**	S7
Preparation of **28**	S8
Preparation of **39b**	S8
Preparation of **29**	S8
Preparation of **41**	S9
Preparation of **39c**	S9
Preparation of **30**	S9
NMR Spectra	S10 – S29
HPLC Chromatograms	S30 – S39
Timecourse of β-FNA antagonism of fentanyl	S40

General Information. Reactions were performed in oven-dried glassware under normal atmosphere (unless otherwise specified). All chemical reagents were purchased from commercial suppliers and used without further purification. Anhydrous solvents were either purchased from commercial suppliers or obtained from a solvent purification system in which solvent was passed through two columns of activated alumina under nitrogen. Reactions were monitored by thin-layer chromatography (TLC) on 0.25 mm Analtech GHLF silica gel plates and visualized by UV (254 nm). Flash column chromatography was performed on a CombiFlash NextGen 300+ purification system (Teledyne Isco) using prepacked *RediSepRf* silica columns. ^{1}H and ^{13}C NMR were recorded on a 400 MHz Varian spectrometer. Chemical shifts (δ) are reported in ppm and referenced with respect to residual solvent: $CDCl_3$ at 7.26 ppm, MeOD at 3.31 ppm (^{1}H NMR); $CDCl_3$ at 77.16 ppm, MeOD at 49.00 ppm (^{13}C NMR). Coupling constants (*J*) are reported in Hz. High-resolution mass spectra (HRMS) were obtained on an Agilent 6230 time-of-flight mass spectrometer with an electrospray ion source in positive mode. Compounds were identified as ≥ 95% pure by HPLC on an Agilent 1260 Infinity II with diode array detection at 214 nm. A Poroshell 120 EC-C18 column (4.6 × 100 mm, 2.7 μm) with a gradient mobile phase of 5-100% acetonitrile / 0.1% phosphoric acid in water was utilized.

General Procedure for the Formation of Oxalate Salt. Free base compounds were dissolved in MeOH, followed by the addition of oxalic acid (1 equiv). Once in solution, MeOH was removed *in vacuo* and diethyl ether added, upon which the oxalate salt precipitated out of solution. The precipitate was collected via vacuum filtration and dried under vacuum to afford the corresponding oxalate salt.

(4a*S*,7a*R*,12b*S*)-9-((*tert*-butyldimethylsilyl)oxy)-3-(cyclopropylmethyl)-7-oxo-1,2,3,4,5,6,7,7a-octahydro-4a*H*-4,12-methanobenzofuro[3,2-*e*]isoquinolin-4a-yl propionate (36). To a solution of (4a*S*,7a*R*,12b*S*)-9-((*tert*-butyldimethylsilyl)oxy)-3-(cyclopropylmethyl)-4a-hydroxy-2,3,4,4a,5,6-hexahydro-1*H*-4,12-methanobenzofuro [3,2-*e*]isoquinolin-7(7a*H*)-one (365 mg, 0.801 mmol) in toluene (10 mL) was added Et_3N (0.167 mL, 1.202 mmol) and propionic anhydride (0.155 mL, 1.202 mmol). The reaction stirred at reflux for 16 h. The resulting solution was cooled to room temperature and washed with sat. aq. $NaHCO_3$ (2 × 15 mL) and water (1 × 15 mL). The organic layer was dried over Na_2SO_4, filtered, and concentrated. The resulting residue was purified by column chromatography (0-20% EtOAc/Hexanes) to afford **36** (280 mg, 0.547 mmol, 68.3%) as a yellow solid. ^{1}H NMR (400 MHz, $CDCl_3$) δ 6.64 (d, *J* = 8.1 Hz, 1H), 6.55 (d, *J* = 8.1 Hz, 1H), 4.59 (s, 1H), 4.45 (d, *J* = 5.6 Hz, 1H), 3.07 (d, *J* = 18.6 Hz, 1H), 2.83 (ddd, *J* = 14.3, 5.5, 2.8 Hz, 1H), 2.70 (dd, *J* = 11.9, 5.0 Hz, 1H), 2.61 (td, *J* = 15.0, 5.3 Hz, 1H), 2.54 – 2.42 (m, 4H), 2.38 – 2.22 (m, 3H), 2.13 (td, *J* = 12.0, 3.7 Hz, 1H), 1.66 (td, *J* = 14.4, 4.0 Hz, 1H), 1.48 (dd, *J* = 12.4, 3.7 Hz, 1H), 1.22 (td, *J* = 7.6, 1.0 Hz, 3H), 1.00 (s, 9H), 0.76 (m, 1H), 0.48 (m, 2H), 0.26 (s, 3H), 0.19 (s, 3H), 0.08 (m, 2H). ^{13}C NMR (100 MHz, $CDCl_3$) δ 207.24, 173.67, 146.91, 138.13, 128.78, 126.44, 122.64, 119.42, 89.67, 82.29, 59.58, 55.78, 51.09, 44.00, 35.78, 30.45, 28.90, 27.07, 25.85 (3C), 23.37, 18.39, 9.61, 9.55, 3.97, 3.86, –4.39, –4.56.

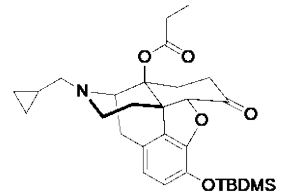

(4R,4aS,7aR,12bS)-3-(cyclopropylmethyl)-9-hydroxy-7-oxo-1,2,3,4,5,6,7,7a-octahydro-4aH-4,12-methanobenzofuro[3,2-e]isoquinolin-4a-yl propionate (14). To a solution of **36** (260 mg, 0.508 mmol) in CH_2Cl_2 (1.0 mL) was added KF (74 mg) and MeOH (10 mL). After stirring at room temperature for 16 h, the reaction was concentrated and purified by column chromatography (30-40% EtOAc/Hexanes) to afford **14** (93 mg, 0.234 mmol, 46.1%). ^{1}H NMR (400 MHz, $CDCl_3$) δ 6.73 (d, *J* = 8.1 Hz, 1H), 6.60 (d, *J* = 8.3 Hz, 1H), 5.73 (bs, 1H), 4.70 (d, *J* = 2.3 Hz, 1H), 4.45 (m, 1H), 3.08 (d, *J* = 18.4 Hz, 1H), 2.85 (m, 1H), 2.74 – 2.41 (m, 6H), 2.30 (m, 3H), 2.16 (m, 1H), 1.65 (t, *J* = 14.3 Hz, 1H), 1.52 (m, 1H), 1.23 (td, *J* = 7.6, 2.4 Hz, 3H), 0.76 (m, 1H), 0.49 (m, 2H), 0.07 (m, 2H). ^{13}C NMR (100 MHz, $CDCl_3$) δ 208.55, 173.61, 143.50, 138.70, 128.34, 125.55, 120.09, 117.92, 90.45, 82.25, 59.64, 55.83, 51.48, 44.00, 35.89, 30.30, 28.90, 27.18, 23.26, 9.67, 9.55, 4.01, 3.82. HRMS calculated for $C_{23}H_{27}NO_5$ $[M+H]^+$: 398.1959 (found); 398.1962 (calcd). HPLC purity: > 99%.

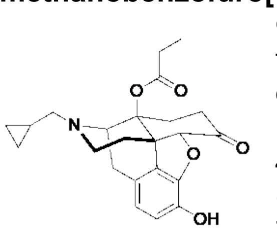

(4a'*S*,7a'*R*,12b'*S*)-9'-(benzyloxy)-4a'-(((*E*)-3-cyclohexylallyl)oxy)-3'-(cyclopropylmethyl)-2',3',4',4a',5',6'-hexahydro-1'*H*,7a'*H*-spiro[[1,3]dioxolane-2,7'-[4,12]methanobenzofuro[3,2-*e*]isoquinoline] (32b). To a solution of (4a'*S*,7a'*R*,12b'*S*)-9'-(benzyloxy)-3'-(cyclopropylmethyl)-1',2',3',4',5',6'-hexahydro-4a'*H*,7a'*H*-spiro[[1,3]dioxolane-2,7'-[4,12]methanobenzofuro[3,2-*e*]isoquinolin]-4a'-ol (400 mg, 0.841 mmol) in DMF (8.0 mL) was added NaH (81 mg, 3.364 mmol) (60% dispersion in mineral oil). The reaction mixture was stirred at room temperature for 10 minutes, followed by the addition of (3-bromoprop-1-en-1-yl)cyclohexane (480 mg, 2.363 mmol). After stirring at room temperature for 16 h, the reaction mixture was diluted with EtOAc (20 mL) and poured into sat. aq. $NaHCO_3$ (30 mL). The organic layer was collected, and the aqueous layer extracted with EtOAc (2 × 20 mL). The combined organic extracts were dried over Na_2SO_4, filtered, and concentrated. The resulting residue was purified by column chromatography (0-15% EtOAc/Hexanes) to afford **32b** (85 mg, 0.142 mmol, 16.9%) as a clear oil. ^1H NMR (400 MHz, $CDCl_3$) δ 7.43 (m, 2H), 7.35 (m, 2H), 7.30 (m, 1H), 6.75 (d, *J* = 8.2 Hz, 1H), 6.54 (d, *J* = 8.1 Hz, 1H), 5.59 (m, 2H), 5.19 (d, *J* = 11.9 Hz, 1H), 5.11 (d, *J* = 11.9 Hz, 1H), 4.63 (s, 1H), 4.17 (m, 1H), 4.06 (m, 1H), 3.89 (m, 1H), 3.77 (m, 2H), 3.45 (d, *J* = 4.9 Hz, 1H), 3.08 (d, *J* = 18.1 Hz, 1H), 2.65 (dd, *J* = 11.5, 5.2 Hz, 1H), 2.57 (td, *J* = 11.9, 5.2 Hz, 1H), 2.35 (m, 3H), 2.19 (td, *J* = 13.5, 2.9 Hz, 1H), 2.07 (m, 1H), 1.96 (m, 1H), 1.74 (m, 6H), 1.65 (m, 2H), 1.40 (dt, *J* = 13.2, 3.3 Hz, 1H), 1.31 (m, 1H), 1.27 (m, 2H), 1.17 (m, 1H), 1.09 (m, 2H), 0.86 (m, 1H), 0.48 (m, 2H), 0.12 (m, 2H). ^{13}C NMR (100 MHz, $CDCl_3$) δ 138.96, 138.25, 128.57 (2C), 127.89, 127.57 (2C), 126.44, 125.30, 118.12, 115.86, 109.30, 94.18, 75.49, 61.01, 59.65, 56.13, 52.35, 45.15, 40.51 (2C), 38.52, 33.69, 33.39, 33.02, 32.96, 28.96, 26.94, 26.33, 26.26 (2C), 26.12 (2C), 23.64, 23.16, 9.61, 4.02, 3.82.

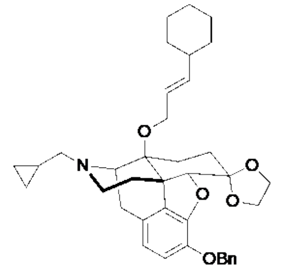

(4R,4aS,7aR,12bS)-4a-(3-cyclohexylpropoxy)-3-(cyclopropylmethyl)-9-hydroxy-2,3,4,4a,5,6-hexahydro-1H-4,12-methanobenzofuro[3,2-e]isoquinolin-7(7aH)-one (16). To a solution of **32b** (50 mg, 0.084 mmol) in THF (4.0 mL) was added Palladium on Carbon (5 wt. % loading) (50 mg). The reaction flask was capped with a rubber septum and flushed with H_2. The reaction stirred at room temperature under an H_2 balloon for 16 h. The reaction mixture was then filtered through Celite, rinsing with EtOAc, and concentrated to afford crude **33**. Crude **33** was dissolved in MeOH (2.0 mL) and conc. HCl (1.5 mL) and stirred at reflux for 4 h. Upon cooling to room temperature, the solution was diluted with water and treated with 5 N NaOH / solid NH_4Cl until pH = 8. The product was then extracted using EtOAc (3 × 10 mL). The combined organic extracts were dried over Na_2SO_4, filtered, and concentrated. The resulting residue was purified by column chromatography (0-30% EtOAc/Hexanes) to afford **16** (20 mg, 0.043 mmol, 51.2% over 2 steps) as a light brown solid. ^1H NMR (400 MHz, $CDCl_3$) δ 6.69 (d, *J* = 8.1 Hz, 1H), 6.56 (d, *J* = 8.1 Hz, 1H), 4.66 (s, 1H), 3.71 (q, *J* = 7.0 Hz, 1H), 3.52 (m, 1H), 3.29 (q, *J* = 6.4 Hz, 1H), 3.11 (d, *J* = 18.3 Hz, 1H), 2.83 (td, *J* = 14.4, 5.2 Hz, 1H), 2.73 – 2.64 (m, 2H), 2.38 – 2.37 (m, 3H), 2.18 (dt, *J* = 14.7, 3.2 Hz, 1H), 2.12 – 2.01 (m, 2H), 1.77 – 1.61 (m, 7H), 1.43 (m, 2H), 1.33 (m, 4H), 1.19 (m, 3H), 0.86 (m, 2H), 0.51 (m, 2H), 0.13 (m, 2H). ^{13}C NMR (100 MHz, $CDCl_3$) δ 210.35, 143.61, 138.76, 129.69, 125.48, 119.88, 117.58, 90.88, 75.28, 60.24, 59.72, 56.10, 51.43, 44.70, 37.66, 35.87, 34.43, 33.65, 33.58, 29.48, 27.77, 26.86 (2C), 26.60 (2C), 25.94, 23.16, 9.50, 3.96. HRMS calculated for $C_{29}H_{39}NO_4$ $[M+H]^+$: 466.2959 (found); 466.2952 (calcd). HPLC purity: > 99%.

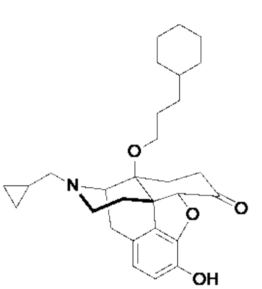

(4a'*S*,7a'*R*,12b'*S*)-9'-(benzyloxy)-3'-(cyclopropylmethyl)-4a'-propoxy-2',3',4',4a',5',6'-hexahydro-1'*H*,7a'*H*-spiro[[1,3]dioxolane-2,7'-[4,12]methanobenzofuro[3,2-e]isoquinoline] (32a). To a solution of (4a'*S*,7a'*R*,12b'*S*)-9'-(benzyloxy)-3'-(cyclopropylmethyl)-1',2',3',4',5',6'-hexahydro-4a'*H*,7a'*H*-spiro[[1,3]dioxolane-2,7'-[4,12]methanobenzofuro[3,2-*e*]isoquinolin]-4a'-ol (250 mg, 0.526 mmol) in DMF (5 mL) was added NaH (60% dispersion in mineral oil) (50.5 mg, 2.103 mmol). The reaction mixture was stirred at room temperature for 10 minutes, followed by the addition of dipropyl sulfate (0.346 mL, 2.103 mmol). After stirring at room temperature for 16 h, the reaction mixture was diluted with EtOAc (5 mL) and poured into sat. aq. $NaHCO_3$ (10 mL). The organic layer was collected, and the aqueous layer extracted with EtOAc (2 × 10 mL). The combined organic extracts were dried over Na_2SO_4, filtered, and concentrated. The resulting residue was purified by column chromatography (5-8% EtOAc/Hexanes) to afford **32a** (0.170 g, 0.328 mmol, 62.5%) as a clear oil. ^1H NMR (400 MHz, $CDCl_3$) δ 7.44 (m, 2H), 7.37 (m, 2H), 7.31 (m, 1H), 6.77 (d, *J* = 8.2 Hz, 1H), 6.56 (d, *J* = 8.1 Hz, 1H), 5.21 (d, *J* = 12.0 Hz, 1H), 5.13 (d, *J* = 12.0 Hz, 1H),

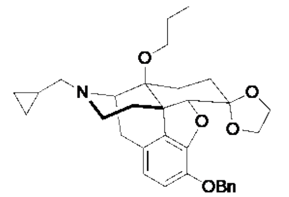

4.63 (s, 1H), 4.19 (q, 1H), 4.08 (q, 1H), 3.90 (q, 1H), 3.79 (q, 1H), 3.57 (q, 1H), 3.43 (d, J = 4.8 Hz, 1H), 3.21 (q, 1H), 3.10 (d, J = 18.1 Hz, 1H), 2.67 (m, 1H), 2.60 (td, J = 11.8, 5.3 Hz, 1H), 2.42 – 2.27 (m, 3H), 2.18 (td, J = 13.5, 2.9 Hz 1H), 2.07 (td, J = 11.6, 4.0 Hz, 1H), 1.78 (dt, J = 13.8, 3.1 Hz, 1H), 1.65 (m, 2H), 1.42 (dt, J = 13.1, 3.1 Hz, 1H), 1.32 (m, 2H), 1.01 (t, J = 7.4 Hz, 3H), 0.85 (m, 1H), 0.50 (m, 2H), 0.13 (m, 2H). ^{13}C NMR (100 MHz, $CDCl_3$) δ 146.7, 141.3, 137.7, 131.8, 128.5 (2C), 127.8, 127.4 (2C), 126.4, 118.0, 115.7, 109.2, 94.1, 74.6, 71.6, 66.5, 64.9, 60.6, 59.6, 56.1, 48.8, 45.0, 29.8, 28.8, 23.6, 23.3, 23.1, 11.2, 9.5, 3.9, 3.7.

(4R,4aS,7aR,12bS)-3-(cyclopropylmethyl)-9-hydroxy-4a-propoxy-2,3,4,4a,5,6-hexahydro-1H-4,12-methanobenzofuro[3,2-e]isoquinolin-7(7aH)-one (20). A solution of **32a** (62 mg, 0.120 mmol) in MeOH (2 mL) and conc. HCl (1.5 mL) was refluxed for 4 hours. The reaction was cooled to room temperature, diluted with water, and treated with 5 N NaOH / solid NH_4Cl until pH = 8. The product was then extracted using EtOAc (3 × 10 mL). The combined organic extracts were dried over Na_2SO_4, filtered, and concentrated. The resulting residue was purified by column chromatography (0-2% MeOH/CH_2Cl_2) to afford **20** (45 mg, 0.117 mmol, 98.0%) as a light-yellow solid. ^{1}H NMR (400 MHz, $CDCl_3$) δ 6.69 (d, J = 8.1 Hz, 1H), 6.56 (d, J = 8.1 Hz, 1H), 4.67 (s, 1H), 3.69 (q, 1H), 3.52 (d, J = 5.3 Hz, 1H), 3.29 (q, 1H), 3.12, (d, J = 18.3 Hz, 1H), 2.83 (m, 1H), 2.76 – 2.63 (m, 2H), 2.43 – 2.26 (m, 3H), 2.18 (dt, J = 14.7, 3.1 Hz, 1H), 2.14 – 2.00 (m, 2H), 1.68 (m, 2H), 1.48 – 1.37 (m, 2H), 1.01 (t, J = 7.4 Hz, 3H), 0.86 (m, 1H), 0.51 (m, 2H), 0.13 (m, 2H). ^{13}C NMR (100 MHz, $CDCl_3$) δ 210.28, 143.62, 138.70, 129.72, 125.52, 119.85, 117.55, 90.88, 75.25, 61.51, 59.74, 56.18, 51.44, 44.65, 35.81, 29.48, 25.94, 23.69, 23.17, 11.28, 9.52, 4.01, 3.88. HRMS calculated for $C_{23}H_{29}NO_4$ $[M+H]^+$: 384.2165 (found); 384.2169 (calcd). HPLC purity: 95.95%.

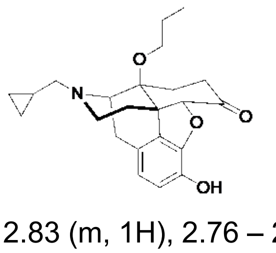

(4a'*S*,7a'*R*,12b'*S*)-4a'-(allyloxy)-9'-(benzyloxy)-3'-(cyclopropylmethyl)-2',3',4',4a',5',6'-hexahydro-1'*H*,7a'*H*-spiro[[1,3]dioxolane-2,7'-[4,12]methanobenzofuro[3,2-e]isoquinoline] (34). To a solution of (4a'*S*,7a'*R*,12b'*S*)-9'-(benzyloxy)-3'-(cyclopropylmethyl)-1',2',3',4',5',6'-hexahydro-4a'*H*,7a'*H*-spiro[[1,3]dioxolane-2,7'-[4,12]methanobenzofuro[3,2-*e*]isoquinolin]-4a'-ol (1.528 g, 3.213 mmol) in DMF (10 mL) was added NaH (60% dispersion in mineral oil) (308 mg, 12.852 mmol). The reaction mixture was stirred at room temperature for 10 minutes, followed by the addition of allyl bromide (1.10 mL, 12.852 mmol). After stirring at room temperature for 16 h, the reaction mixture was diluted with EtOAc (10 mL) and poured into sat. aq. $NaHCO_3$ (50 mL). The organic layer was collected, and the aqueous layer extracted with EtOAc (2 × 20 mL). The combined organic extracts were dried over Na_2SO_4, filtered, and concentrated. The resulting residue was purified by column chromatography (10-20% EtOAc/Hexanes) to afford **34** (1.260 g, 2.444 mmol, 76.1%) as a clear oil. ^{1}H NMR (400 MHz, $CDCl_3$) δ 7.43 (m, 2H), 7.36 (m, 2H), 7.30 (m, 1H), 6.75 (d, J = 8.3 Hz, 1H), 6.54 (d, J = 8.1 Hz, 1H), 6.01 (m, 1H), 5.33 (dq, J = 17.1, 1.8 Hz, 1H), 5.19 (d, J = 11.9 Hz, 1H), 5.12 (m, 2H), 4.63 (s, 1H), 4.23 – 4.15 (m, 2H), 4.06 (q, J = 6.5 Hz, 1H), 3.88 (m, 1H), 3.84 – 3.74 (m, 2H), 3.45 (d, J = 4.9 Hz, 1H), 3.09 (d, J = 18.1 Hz, 1H), 2.68 – 2.53 (m, 2H), 2.39 – 2.31 (m, 3H), 2.18 (td, J = 13.4, 2.9 Hz, 1H), 2.06 (m, 1H), 1.76 (dt, J = 14.1, 3.3 Hz, 1H), 1.44 – 1.28 (m, 3H), 0.83 (m, 1H), 0.48 (m, 2H), 0.12 (m, 2H). ^{13}C NMR (100 MHz, $CDCl_3$) δ 146.82, 141.43, 137.78, 131.69, 128.56 (2C), 127.88, 127.55 (2C), 126.35, 118.14, 115.94, 115.19, 109.21, 94.15, 75.53, 71.72, 66.62, 65.04, 60.82, 59.64, 56.20, 48.81, 45.09, 30.00, 28.94, 23.58, 23.14, 14.33, 9.58, 3.94, 3.85.

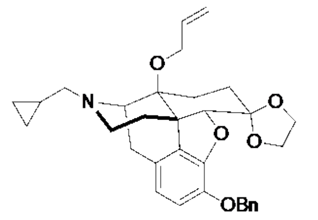

(4R,4aS,7aR,12bS)-4a-(allyloxy)-3-(cyclopropylmethyl)-9-hydroxy-2,3,4,4a,5,6-hexahydro-1H-4,12-methanobenzofuro[3,2-e]isoquinolin-7(7aH)-one (21). A solution of **34** (242 mg, 0.469 mmol) in MeOH (2.5 mL) and conc. HCl (1.5 mL) was refluxed for 4 hours. The reaction was cooled to room temperature, diluted with water, and treated with 5 N NaOH / solid NH_4Cl until pH = 8. The product was then extracted using EtOAc (3 × 10 mL). The combined organic extracts were dried over Na_2SO_4, filtered, and concentrated. The resulting residue was purified by column chromatography (0-2% MeOH/CH_2Cl_2) to afford **21** (146 mg, 0.383 mmol, 81.6%) as a yellow solid. ^{1}H NMR (400 MHz, $CDCl_3$) 6.70 (d, J = 8.2 Hz, 1H), 6.57 (d, J = 8.2 Hz, 1H), 6.05 (m, 1H), 5.36 (m, 1H), 5.18 (m, 1H), 4.70 (s, 1H), 4.35 (dd, J = 11.8, 4.9 Hz, 1H), 3.92 (dd, J = 11.8, 5.4 Hz, 1H), 3.55 (d, J = 5.3 Hz, 1H), 3.14 (d, J = 18.3 Hz, 1H), 2.86 (m, 1H), 2.72 (m, 2H), 2.39 – 2.30 (m, 3H), 2.20 (dt, J = 14.9, 3.1 Hz, 1H), 2.15 – 2.01 (m, 2H), 1.46 (m, 2H), 0.87 (m, 1H), 0.52 (m, 2H), 0.14 (m, 2H). ^{13}C NMR (100 MHz, $CDCl_3$) δ 209.75, 143.57, 138.67, 135.70, 129.61, 125.42, 119.86,

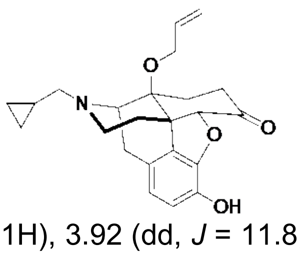

117.50, 115.81, 90.84, 76.04, 61.53, 59.66, 56.17, 51.41, 44.70, 35.88, 29.59, 26.08, 23.18, 9.51, 4.01, 3.89. HRMS calculated for $C_{23}H_{27}NO_4$ $[M+H]^+$: 382.2019 (found); 382.2013 (calcd). HPLC purity: > 99%.

3-(((4a'*S*,7a'*R*,12b'*S*)-9'-(benzyloxy)-3'-(cyclopropylmethyl)-1',2',3',4',5',6'-hexahydro-4a'*H*,7a'*H*-spiro[[1,3]dioxolane-2,7'-[4,12]methanobenzofuro[3,2-e]isoquinolin]-4a'-yl)oxy)propane-1,2-diol (35). To a solution of AD-mix-α (2.335 g; 1.4 g per mmol olefin) in 1:1 $tBuOH/H_2O$ (25 mL) was added

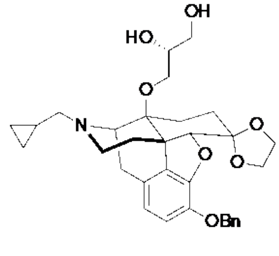

methanesulfonamide (159 mg, 1.668 mmol). The reaction was cooled to 0 °C, followed by the addition of **34** (860 mg, 1.668 mmol). After 5 min, the ice bath was removed and the solution stirred at room temperature for 16 h. To the resulting yellow suspension was added Na_2SO_3 (2.50 g; 1.5 g per mmol olefin) at 0 °C. The reaction stirred at room temperature for 30 min, then basified using 1 N NaOH and extracted with DCM (3 × 20 mL). The combined organic extracts were dried over Na_2SO_4, filtered, and concentrated. The resulting residue was purified by column chromatography (0-5% $MeOH/CH_2Cl_2$) to afford **35** (620 mg, 1.128 mmol, 67.6%) as a white solid. ^{1}HMR (400 MHz, $CDCl_3$) δ 7.42 (m, 2H), 7.36 (m, 2H), 7.30 (m, 1H), 6.77 (d, *J* = 8.2 Hz, 1H), 6.56 (d, *J* = 8.2 Hz, 1H), 5.19 (d, *J* = 11.9 Hz, 1H), 5.12 (d, *J* = 12.0 Hz, 1H), 4.63 (s, 1H), 4.18 (m, 1H), 4.05 (m, 1H), 3.96 – 3.86 (m, 2H), 3.77 (m, 2H), 3.58 – 3.45 (m, 4H), 3.13 (d, *J* = 18.4 Hz, 1H), 2.91 (dd, *J* = 12.2, 5.0 Hz, 1H), 2.58 (m, 2H), 2.44 (dd, *J* = 18.4, 5.2 Hz, 1H), 2.14 (dd, *J* = 12.5, 7.6 Hz, 1H), 2.02 (m, 2H), 1.71 (m, 1H), 1.52 – 1.36 (m, 3H), 0.94 (m, 1H), 0.53 (m, 2H), 0.12 (m, 2H). ^{13}C NMR (100 MHz, $CDCl_3$) δ 146.90, 141.66, 137.61, 130.93, 128.60 (2C), 127.96, 127.54 (2C), 125.49, 118.25, 116.32, 108.75, 93.81, 75.74, 71.74, 70.61, 66.69, 65.12, 63.56, 63.20, 60.04, 56.14, 48.45, 44.59, 29.56, 28.78, 24.47, 23.18, 8.41, 5.15, 3.24.

(4*R*,4a*S*,7a*R*,12b*S*)-3-(cyclopropylmethyl)-4a-((*R*)-2,3-dihydroxypropoxy)-9-hydroxy-2,3,4,4a,5,6-hexahydro-1*H*-4,12-methanobenzofuro[3,2-*e*]isoquinolin-7(7a*H*)-one (22). A solution of **35** (600 mg, 1.092 mmol) in MeOH (5.5 mL) and conc. HCl (3.0 mL) was refluxed for 4 hours. The reaction was cooled to room

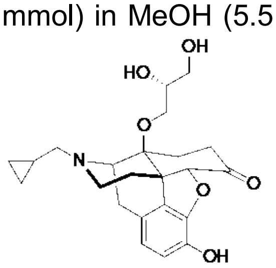

temperature, diluted with water, and treated with 5 N NaOH / solid NH_4Cl until pH = 8. The product was then extracted using EtOAc (3 × 15 mL). The combined organic extracts were dried over Na_2SO_4, filtered, and concentrated. The resulting residue was purified by column chromatography (0-5% $MeOH/CH_2Cl_2$) to afford **22** (420 mg, 1.011 mmol, 92.6%) as a white solid. ^{1}H NMR (400 MHz, MeOD) δ 6.63 (d, *J* = 8.0 Hz, 1H), 6.58 (d, *J* = 8.2 Hz, 1H), 4.77 (s, 1H), 3.94 (m, 1H), 3.78 – 3.59 (m, 5H), 3.20 (d, *J* = 18.7 Hz, 1H), 2.87 (m, 2H), 2.73 (td, *J* = 12.4, 5.2 Hz, 1H), 2.54 – 2.42 (m, 2H), 2.29 (dd, *J* = 12.6, 6.9 Hz, 1H), 2.20 – 2.04 (m, 3H), 1.46 (m, 2H), 0.98 (m, 1H), 0.54 (m, 2H), 0.18 (m, 2H). ^{13}C NMR (100 MHz, MeOD) δ 211.49, 145.06, 140.94, 130.35, 125.50, 120.75, 119.10, 91.38, 77.15, 72.17, 64.02, 63.74, 60.51, 56.31, 52.31, 45.69, 36.18, 30.36, 27.69, 23.68, 8.96, 4.76, 4.18. HRMS calculated for $C_{23}H_{29}NO_6$ $[M+H]^+$: 416.2069 (found); 416.2068 (calcd). HPLC purity: 98.23%.

(4a*S*,7*S*,7a*R*,12b*S*)-9-(benzyloxy)-3-(cyclopropylmethyl)-1,2,3,4,5,6,7,7a-octahydro-4a*H*-4,12-methanobenzofuro[3,2-e]isoquinoline-4a,7-diol (40a). To a solution of α-naltrexol (410 mg, 1.194 mmol) in

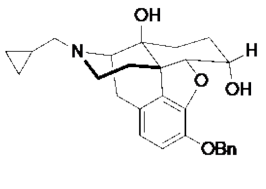

DMF (9.5 mL) was added K_2CO_3 (495 mg, 3.582 mmol) and benzyl bromide (0.14 mL, 1.194 mmol). After stirring at room temperature for 16 h, the reaction mixture was poured into water (200 mL) and extracted with EtOAc (3 × 150 mL). The combined organic extracts were dried over Na_2SO_4, filtered, and concentrated. The resulting residue was purified by column chromatography (0-5% $MeOH/CH_2Cl_2$) to afford **40a** (480 mg, 1.107 mmol, 92.7%) as a white solid. ^{1}H NMR (400 MHz, $CDCl_3$) δ 7.42 (m, 2H), 7.36 (m, 2H), 7.30 (m, 1H), 6.77 (d, *J* = 8.2 Hz, 1H), 6.55 (d, *J* = 8.2 Hz, 1H), 5.23 (d, *J* = 12.2 Hz, 1H), 5.12 (d, *J* = 12.2 Hz, 1H), 4.62 (m, 1H), 4.15 (m, 1H), 3.10 – 2.96 (m, 2H), 2.60 (m, 2H), 2.34 (m, 2H), 2.22 (m, 2H), 1.72 (m, 1H), 1.66 – 1.40 (m, 3H), 1.07 (m, 1H), 0.84 (m, 1H), 0.53 (m, 2H), 0.11 (m, 2H). ^{13}C NMR (100 MHz, $CDCl_3$) δ 147.14, 140.50, 137.65, 132.07, 128.64 (2C), 128.04, 127.53 (2C), 126.92, 119.05, 117.06, 90.87, 72.14, 69.87, 66.84, 62.25, 59.65, 47.01, 43.37, 33.52, 28.45, 23.79, 22.97, 9.51, 4.06, 3.90.

(4a*S*,7*S*,7a*R*,12b*S*)-9-(benzyloxy)-3-(cyclopropylmethyl)-7-methoxy-1,2,3,4,5,6,7,7a-octahydro-4a*H*-4,12-methanobenzofuro[3,2-*e*]isoquinolin-4a-ol (37a). To a solution of **40a** (465 mg, 1.073 mmol) in THF (10 mL) was added NaH (60% dispersion in mineral oil) (46 mg, 1.931 mmol) and iodomethane (0.073 mL, 1.180 mmol).

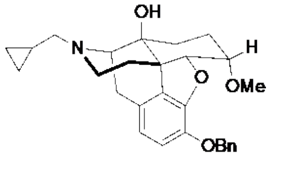

The reaction stirred at room temperature for 16 h, then poured into water (20 mL) and extracted with EtOAc (3 × 20 mL). The combined organic extracts were dried over Na_2SO_4, filtered, and concentrated. The resulting residue was purified by column chromatography (0-2% MeOH/CH_2Cl_2) to afford **37a** (370 mg, 0.827 mmol, 77.1%) as a clear oil. ^{1}H NMR (400 MHz, $CDCl_3$) δ 7.45 (m, 2H), 7.33 (m, 2H), 7.28 (m, 1H), 6.72 (d, *J* = 8.1 Hz, 1H), 6.48 (d, *J* = 8.2 Hz, 1H), 5.21 (dd, *J* = 12.1, 4.9 Hz, 2H), 4.78 (d, *J* = 4.4 Hz, 1H), 3.82 (dt, *J* = 10.7, 4.1 Hz, 1H), 3.46 (s, 3H), 3.07 (d, *J* = 6.5 Hz, 1H), 3.02 (d, *J* = 18.5 Hz, 1H), 2.65 – 2.54 (m, 2H), 2.34 (m, 2H), 2.24 (m, 2H), 1.75 (m, 1H), 1.67 – 1.55 (m, 2H), 1.47 (m, 1H), 1.24 (m, 1H), 0.85 (m, 1H), 0.53 (m, 2H), 0.13 (m, 2H). ^{13}C NMR (100 MHz, $CDCl_3$) δ 148.08, 140.58, 137.84, 131.72, 128.45 (2C), 127.78 (2C), 127.77, 126.68, 118.27, 117.43, 88.71, 75.98, 71.94, 70.09, 62.31, 59.64, 57.32, 47.24, 43.43, 33.60, 28.70, 22.96, 20.64, 9.51, 4.07, 3.90.

(4R,4aS,7S,7aR,12bS)-3-(cyclopropylmethyl)-7-methoxy-1,2,3,4,5,6,7,7a-octahydro-4aH-4,12-methanobenzofuro[3,2-e]isoquinoline-4a,9-diol (25). To a solution of **37a** (280 mg, 0.626 mmol) in THF (11 mL) was added Palladium on Carbon (5 wt. % loading) (100 mg). The reaction flask was capped with a rubber septum and flushed with H_2. The reaction stirred at room temperature under an H_2 balloon for 16 h. The reaction mixture was then filtered through Celite, rinsing with EtOAc, and concentrated. The resulting residue was purified by column chromatography (5% MeOH/CH_2Cl_2) to afford **25** (208 mg, 0.582 mmol, 93.0%) as a white foam. ^{1}H NMR (400 MHz, $CDCl_3$) δ 6.70 (d, *J* = 8.1 Hz, 1H), 6.51 (d, *J* = 8.2 Hz, 1H), 4.76 (d, *J* = 3.3 Hz, 1H), 3.84 (dt, *J* = 12.1, 4.1 Hz, 1H), 3.46 (s, 3H), 3.08 (d, *J* = 6.8 Hz, 1H), 3.03 (d, *J* = 18.5 Hz, 1H), 2.64 (m, 1H), 2.58 (m, 1H), 2.34 (m, 2H), 2.25 (m, 2H), 1.76 – 1.56 (m, 3H), 1.45 (m, 1H), 1.12 (m, 1H), 0.84 (m, 1H), 0.54 (m, 2H), 0.13 (m, 2H). ^{13}C NMR (100 MHz, $CDCl_3$) δ 145.98, 137.62, 130.83, 125.43, 118.81, 117.44, 88.87, 76.02, 70.13, 62.19, 59.67, 57.02, 47.75, 43.28, 33.76, 29.11, 22.93, 19.77, 9.46, 4.07, 3.86. HRMS calculated for $C_{21}H_{27}NO_4$ $[M+H]^+$: 358.2012 (found); 358.2013 (calcd). HPLC purity: 95.38%.

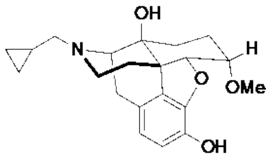

(4a*S*,7*R*,7a*R*,12b*S*)-9-(benzyloxy)-3-(cyclopropylmethyl)-1,2,3,4,5,6,7,7a-octahydro-4a*H*-4,12-methanobenzofuro[3,2-*e*]isoquinoline-4a,7-diol (40b). To a solution of β-naltrexol (510 mg, 1.485 mmol) in DMF (12.0 mL) was added K_2CO_3 (616 mg, 4.455 mmol) and benzyl bromide (0.18 mL, 1.485 mmol). After stirring at room temperature for 16 h, the reaction mixture was poured into water (200 mL) and extracted with EtOAc (3 × 150 mL). The combined organic extracts were dried over Na_2SO_4, filtered, and concentrated. The resulting residue was purified by column chromatography (0-5% MeOH/CH_2Cl_2) to afford **40b** (530 mg, 1.222 mmol, 82.3%) as a clear oil. ^{1}H NMR (400 MHz, $CDCl_3$) δ 7.38 (m, 2H), 7.30 (m, 2H), 7.25 (m, 1H), 6.73 (d, *J* = 8.2 Hz, 1H), 6.53 (d, *J* = 8.2 Hz, 1H), 5.18 (d, *J* = 12.1 Hz, 1H), 5.11 (d, *J* = 12.1 Hz, 1H), 4.43 (d, *J* = 5.8 Hz, 1H), 3.45 (m, 1H), 3.05 (d, *J* = 5.8 Hz, 1H), 2.98 (d, *J* = 18.5 Hz, 1H), 2.56 (m, 2H), 2.32 (d, *J* = 6.5 Hz, 2H), 2.20 (td, *J* = 12.5, 5.0 Hz, 1H), 2.08 (td, *J* = 12.0, 3.5 Hz, 1H), 1.90 (m, 1H), 1.58 (dt, *J* = 13.3, 4.2 Hz, 1H), 1.52 (dt, *J* = 13.1, 4.4 Hz, 1H), 1.45 (m, 1H), 1.29 (m, 1H), 0.81 (m, 1H), 0.50 (m, 2H), 0.09 (m, 2H). ^{13}C NMR (100 MHz, $CDCl_3$) δ 144.67, 142.21, 137.57, 132.35, 128.42 (2C), 127.84, 127.57 (2C), 126.06, 118.66, 116.97, 95.68, 72.10, 71.85, 70.13, 62.20, 59.31, 47.00, 43.77, 31.35, 29.26, 25.34, 22.75, 9.46, 3.95, 3.85.

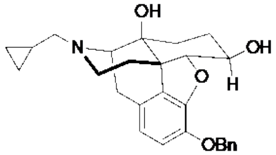

(4a*S*,7*R*,7a*R*,12b*S*)-9-(benzyloxy)-3-(cyclopropylmethyl)-7-methoxy-1,2,3,4,5,6,7,7a-octahydro-4a*H*-4,12-methanobenzofuro[3,2-*e*]isoquinolin-4a-ol (37b). To a solution of **40b** (470 mg, 1.084 mmol) in THF (10 mL) was added NaH (60% dispersion in mineral oil) (47 mg, 1.951 mmol) and iodomethane (0.074 mL, 1.192 mmol). The reaction stirred at room temperature for 16 h, then poured into water (20 mL) and extracted with EtOAc (3 × 20 mL). The combined organic extracts were dried over Na_2SO_4, filtered, and concentrated. The resulting residue was purified by column chromatography (0-2% MeOH/CH_2Cl_2) to afford **37b** (360 mg, 0.804 mmol, 74.2%) as a clear oil. ^{1}H NMR (400 MHz, $CDCl_3$) δ 7.41 (m, 2H), 7.33 (m, 2H), 7.28 (m, 1H), 6.74 (d, *J* = 8.1 Hz, 1H), 6.54 (d, *J* = 8.2 Hz, 1H), 5.20 (s, 2H), 4.51 (d, *J* = 6.3 Hz, 1H), 3.45 (s, 3H), 3.10 (m, 2H), 2.99 (d, *J* = 18.3 Hz, 1H), 2.58 (m, 2H), 2.34 (d, *J* = 6.5 Hz, 2H), 2.22 (td, *J* = 12.5, 5.2 Hz, 1H), 2.06 (td, *J* = 12.2, 3.7 Hz, 1H), 1.87 (m, 1H), 1.78 (m, 1H), 1.58 (m, 1H), 1.45 (m, 1H), 1.33 (td, *J* = 13.5, 3.3 Hz, 1H), 0.81 (m, 1H), 0.51 (m, 2H), 0.10 (m, 2H). ^{13}C NMR (100 MHz, $CDCl_3$) δ 144.61, 142.57, 137.72, 132.70, 128.51

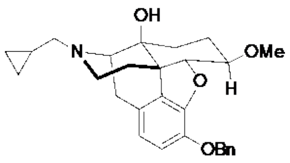

(2C), 127.90, 127.66 (2C), 126.05, 118.58, 117.64, 95.13, 82.10, 72.17, 70.15, 62.40, 59.30, 57.64, 47.67, 43.98, 30.81, 29.69, 23.56, 22.81, 9.57, 4.03, 3.91.

(4R,4aS,7R,7aR,12bS)-3-(cyclopropylmethyl)-7-methoxy-1,2,3,4,5,6,7,7a-octahydro-4aH-4,12-methanobenzofuro[3,2-e]isoquinoline-4a,9-diol (26). To a solution of **37b** (300 mg, 0.670 mmol) in THF (12 mL) was added Palladium on Carbon (5 wt. % loading) (100 mg). The reaction flask was capped with a rubber septum and flushed with H_2. The reaction stirred at room temperature under an H_2 balloon for 16 h. The reaction mixture was then filtered through Celite, rinsing with EtOAc, and concentrated. The resulting residue was purified by column chromatography (5% MeOH/CH_2Cl_2) to afford **26** (212 mg, 0.593 mmol, 88.5%) as a white solid. ^{1}H NMR (400 MHz, $CDCl_3$) δ 6.71 (d, J = 8.1 Hz, 1H), 6.56 (d, J = 8.2 Hz, 1H), 4.52 (d, J = 6.4 Hz, 1H), 3.43 (s, 3H), 3.09 (m, 2H), 3.01 (d, J = 18.3 Hz, 1H), 2.60 (m, 2H), 2.36 (d, J = 6.6 Hz, 2H), 2.24 (td, J = 12.4, 5.0 Hz, 1H), 2.10 (td, J = 12.0, 3.7 Hz, 1H), 1.85 (m, 2H), 1.62 (dt, J = 13.4, 3.4 Hz, 1H), 1.45 (m, 1H), 1.33 (m, 1H), 0.83 (m, 1H), 0.52 (m, 2H), 0.12 (m, 2H). ^{13}C NMR (100 MHz, $CDCl_3$) δ 142.32, 139.47, 131.94, 124.82, 118.99, 116.72, 95.51, 82.18, 70.33, 62.45, 59.34, 57.23, 48.03, 44.03, 30.72, 29.67, 23.04, 22.78, 9.60, 4.07, 3.94. HRMS calculated for $C_{21}H_{27}NO_4$ [M+H]$^+$: 358.2015 (found); 358.2013 (calcd). HPLC purity: 96.95%.

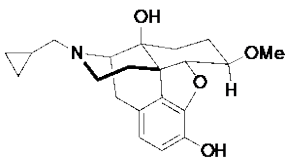

(4a*S*,7a*S*,12b*S*)-9-(benzyloxy)-3-(cyclopropylmethyl)-7-methylene-1,2,3,4,5,6,7,7a-octahydro-4a*H*-4,12-methanobenzofuro[3,2-*e*]isoquinolin-4a-ol (38). To a suspension of methyltriphenylphosphonium bromide (14.869 g, 3.896 mmol) in THF (30 mL) was added tBuOK (5.072 g, 45.196 mmol) in portions over a minute. The resulting yellow suspension stirred at room temperature for 30 minutes, then a solution of (4a*S*,7a*R*,12b*S*)-9-(benzyloxy)-3-(cyclopropylmethyl)-4a-hydroxy-2,3,4,4a,5,6-hexahydro-1*H*-4,12-methanobenzofuro[3,2-*e*]isoquinolin-7(7a*H*)-one (4.610 g, 10.684 mmol) in THF (10 mL) was added dropwise. The reaction stirred at room temperature for 16 h under a nitrogen atmosphere. The reaction mixture was poured into sat. aq. NH_4Cl (40 mL) and extracted with EtOAc (3 × 40 mL). The combined organic extracts were washed with water, dried over Na_2SO_4, filtered, and concentrated. The resulting residue was purified by column chromatography (20% EtOAc/Hexanes) to afford **38** (3.730 g, 8.683 mmol, 81.3 %) as a light-yellow oil. ^{1}H NMR (400 MHz, $CDCl_3$) δ 7.45 (m, 2H), 7.36 (m, 2H), 7.30 (m, 1H), 6.77 (d, J = 8.1 Hz, 1H), 6.56 (d, J = 8.1 Hz, 1H), 5.32 (q, J = 2.0 Hz, 1H), 5.19 (m, 2H), 5.03 (q, J = 1.9 Hz, 1H), 4.89 (q, J = 2.0 Hz, 1H), 3.06 (d, J = 5.8 Hz, 1H), 3.01 (d, J = 18.3 Hz, 1H), 2.70 – 2.52 (m, 3H), 2.37 (d, J = 6.6 Hz, 2H), 2.31 (td, J = 12.5, 5.2 Hz, 1H), 2.15 (m, 2H), 1.63 (dt, J = 12.9, 3.6 Hz, 1H), 1.53 (dd, J = 12.5, 3.8 Hz, 1H), 1.37 (td, J = 13.2, 3.7 Hz, 1H), 0.86 (m, 1H), 0.54 (m, 2H), 0.14 (m, 2H). ^{13}C NMR (100 MHz, $CDCl_3$) δ 145.79, 145.37, 142.20, 137.71, 131.70, 128.51 (2C), 127.87, 127.65 (2C), 127.05, 118.63, 116.95, 111.45, 89.48, 72.00, 70.92, 62.67, 59.31, 48.56, 44.43, 32.38, 30.63, 28.35, 22.84, 9.58, 4.04, 3.90.

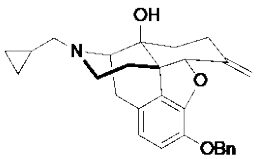

(4a*S*,7a*S*,12b*S*)-4a-(allyloxy)-9-(benzyloxy)-3-(cyclopropylmethyl)-7-methylene-2,3,4,4a,5,6,7,7a-octahydro-1*H*-4,12-methanobenzofuro[3,2-*e*]isoquinoline (39a). To a solution of **38** (1.032 g, 2.402 mmol) in DMF (10 mL) was added NaH (60% dispersion in mineral oil) (231 mg, 9.610 mmol). The reaction mixture was stirred at room temperature for 10 minutes, followed by the addition of allyl bromide (0.83 mL, 9.610 mmol). After stirring at room temperature for 16 h, the reaction mixture was diluted with EtOAc (10 mL) and poured into sat. aq. $NaHCO_3$ (50 mL). The organic layer was collected, and the aqueous layer extracted with EtOAc (2 × 20 mL). The combined organic extracts were dried over Na_2SO_4, filtered, and concentrated. The resulting residue was purified by column chromatography (5-8% EtOAc/Hexanes) to afford **39a** (0.900 g, 1.916 mmol, 79.8%) as a clear oil. ^{1}H NMR (400 MHz, $CDCl_3$) δ 7.45 (m, 2H), 7.36 (m, 2H), 7.31 (m, 1H), 6.72 (d, J = 8.2 Hz, 1H), 6.53 (d, J = 8.1 Hz, 1H), 6.04 (m, 1H), 5.39 – 5.30 (m, 2H), 5.20 (m, 2H), 5.15 (dq, J = 10.4, 1.9 Hz, 1H), 5.06 (m, 1H), 4.85 (m, 1H), 4.25 (dd, J = 12.2, 2.1 Hz, 1H), 3.87 (dd, J = 12.0, 2.1 Hz, 1H), 3.43 (d, J = 5.2 Hz, 1H), 3.09 (d, J = 18.2 Hz, 1H), 2.72 – 2.50 (m, 3H), 2.35 (m, 3H), 2.12 – 1.97 (m, 2H), 1.80 (dq, J = 13.8, 3.1 Hz, 1H), 1.40 (dt, J = 12.3, 2.5 Hz, 1H), 1.18 (m, 1H), 0.86 (m, 1H), 0.50 (m, 2H), 0.13 (m, 2H). ^{13}C NMR (100 MHz, $CDCl_3$) δ 145.97, 145.39, 142.24, 137.82, 136.29, 132.23, 128.54 (2C), 127.87, 127.71 (2C), 127.19, 118.51, 116.80, 115.18, 111.76, 89.60, 76.62, 72.06, 60.97, 59.69, 56.46, 49.05, 45.51, 29.37, 27.91, 26.66, 23.26, 9.58, 3.99, 3.84.

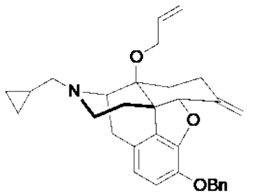

(4R,4aS,7aS,12bS)-4a-(allyloxy)-3-(cyclopropylmethyl)-7-methylene-2,3,4,4a,5,6,7,7a-octahydro-1H-4,12-methanobenzofuro[3,2-e]isoquinolin-9-ol (28). A solution of **39a** (340 mg, 0.724 mmol) in MeOH (3.0 mL) and conc. HCl (2.0 mL) was refluxed for 4 hours. The reaction was cooled to room temperature, diluted with water, and treated with 5 N NaOH / solid NH4Cl until pH = 8. The product was then extracted using EtOAc (3 × 10 mL). The combined organic extracts were dried over Na_2SO_4, filtered, and concentrated. The resulting residue was purified by column chromatography (0-20% EtOAc/Hexanes) to afford **28** (155 mg, 0.408 mmol, 56.4%) as a light yellow solid. ^{1}H NMR (400 MHz, $CDCl_3$) δ 6.66 (d, *J* = 8.2 Hz, 1H), 6.53 (d, *J* = 8.1 Hz, 1H), 6.03 (m, 1H), 5.34 (dq, *J* = 17.2, 1.8 Hz, 1H), 5.24 (q, *J* = 1.9 Hz, 1H), 5.14 (dq, *J* = 10.4, 1.5 Hz, 1H), 5.06 (m, 1H), 4.83 (m, 1H), 4.24 (dd, *J* = 12.1, 4.2 Hz, 1H), 3.85 (m, 1H), 3.43 (d, *J* = 5.3 Hz, 1H), 3.09 (d, *J* = 18.1 Hz, 1H), 2.70 (m, 1H), 2.65 – 2.48 (m, 2H), 2.37 – 2.29 (m, 3H), 2.09 (m, 1H), 2.00 (m, 1H), 1.79 (dt, *J* = 13.8, 3.6 Hz, 1H), 1.38 (m, 1H), 1.15 (td, *J* = 13.5, 3.7 Hz, 1H), 0.85 (m, 1H), 0.49 (m, 2H), 0.12 (m, 2H). ^{13}C NMR (100 MHz, $CDCl_3$) δ 145.80, 143.15, 138.91, 136.27, 131.60, 126.09, 118.97, 116.27, 115.19, 111.60, 90.33, 76.69, 61.00, 59.69, 56.49, 49.37, 45.51, 29.31, 27.86, 26.57, 23.23, 9.57, 4.01, 3.83. HRMS calculated for $C_{24}H_{29}NO_3$ $[M+H]^+$: 380.2220 (found); 380.2220 (calcd). HPLC purity: 97.73%.

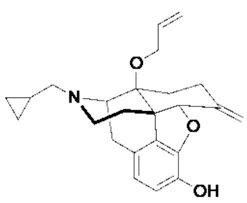

(4a*S*,7a*S*,12b*S*)-9-(benzyloxy)-3-(cyclopropylmethyl)-7-methylene-4a-propoxy-2,3,4,4a,5,6,7,7a-octahydro-1*H*-4,12-methanobenzofuro[3,2-*e*]isoquinoline (39b). To a solution of **38** (340 mg, 0.792 mmol) in DMF (5 mL) was added NaH (60% dispersion in mineral oil) (76.0 mg, 3.166 mmol). The reaction mixture was stirred at room temperature for 10 minutes, followed by the addition of dipropyl sulfate (0.520 mL, 3.166 mmol). After stirring at room temperature for 16 h, the reaction mixture was diluted with EtOAc (5 mL) and poured into sat. aq. $NaHCO_3$ (10 mL). The organic layer was collected, and the aqueous layer extracted with EtOAc (2 × 10 mL). The combined organic extracts were dried over Na_2SO_4, filtered, and concentrated. The resulting residue was purified by column chromatography (5-10% EtOAc/Hexanes) to afford **39b** (0.170 g, 0.360 mmol, 45.5%) as a clear oil. ^{1}H NMR (400 MHz, $CDCl_3$) δ 7.44 (m, 2H), 7.35 (m, 2H), 7.30 (m, 1H), 6.71 (d, *J* = 8.2 Hz, 1H), 6.52 (d, *J* = 7.9 Hz, 1H), 5.31 (q, *J* = 2.0 Hz, 1H), 5.19 (m, 2H), 5.03 (q, *J* = 1.9 Hz, 1H), 4.84 (q, *J* = 2.0 Hz, 1H), 3.58 (m, 1H), 3.40 (d, *J* = 5.1 Hz, 1H), 3.22 (m, 1H), 3.07, (d, *J* = 18.2 Hz, 1H), 2.68 (dd, *J* = 11.4, 5.1 Hz, 1H), 2.63 – 2.48 (m, 2H), 2.38 – 2.26 (m, 3H), 2.09 – 1.95 (m, 2H), 1.80 (m, 1H), 1.63 (m, 2H), 1.37 (m, 1H), 1.13 (t, *J* = 13.5 Hz, 1H), 0.99 (td, *J* = 7.4, 2.0 Hz, 3H), 0.84 (m, 1H), 0.49 (m, 2H), 0.11 (m, 2H). ^{13}C NMR (100 MHz, $CDCl_3$) δ 146.25, 145.41, 142.20, 137.87, 132.39, 128.54 (2C), 127.87, 127.73 (2C), 127.39, 118.48, 116.67, 111.57, 89.71, 75.79, 72.05, 60.94, 59.80, 56.48, 49.09, 45.49, 29.29, 27.87, 26.52, 23.77, 23.27, 11.32, 9.60, 3.97, 3.87.

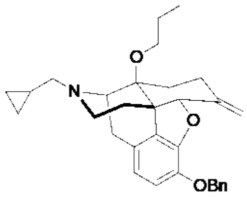

(4R,4aS,7aS,12bS)-3-(cyclopropylmethyl)-7-methylene-4a-propoxy-2,3,4,4a,5,6,7,7a-octahydro-1H-4,12-methanobenzofuro[3,2-e]isoquinolin-9-ol (29). A solution of **39b** (170 mg, 0.360 mmol) in MeOH (2.0 mL) and conc. HCl (1.5 mL) was refluxed for 4 hours. The reaction was cooled to room temperature, diluted with water, and treated with 5 N NaOH / solid NH_4Cl until pH = 8. The product was then extracted using EtOAc (3 × 10 mL). The combined organic extracts were dried over Na_2SO_4, filtered, and concentrated. The resulting residue was purified by column chromatography (0-2% MeOH/ CH_2Cl_2) to afford **29** (102 mg, 0.267 mmol, 74.2%) as a yellow solid. ^{1}H NMR (400 MHz, $CDCl_3$) δ 6.66 (d, *J* = 8.1 Hz, 1H), 6.52 (d, *J* = 8.1 Hz, 1H), 5.24 (m, 1H), 5.03 (m, 1H), 4.82 (m, 1H), 3.58 (m, 1H), 3.40 (d, *J* = 5.2 Hz, 1H), 3.22 (m, 1H), 3.07 (d, *J* = 18.2 Hz, 1H), 2.69 (dd, *J* = 11.5, 5.2 Hz, 1H), 2.64 – 2.45 (m, 2H), 2.39 – 2.27 (m, 3H), 2.09 (m, 1H), 1.98 (dt, *J* = 13.8, 3.5 Hz, 1H), 1.79 (dt, *J* = 13.7, 3.6 Hz, 1H), 1.63 (m, 2H), 1.35 (dd, *J* = 11.8, 3.8 Hz, 1H), 1.10 (m, 1H), 0.98 (t, *J* = 7.4, 1.0 Hz, 3H), 0.86 (m, 1H), 0.48 (m, 2H), 0.11 (m, 2H). ^{13}C NMR (100 MHz, $CDCl_3$) δ 145.99, 143.12, 138.93, 131.71, 126.16, 118.98, 116.25, 111.43, 90.40, 75.84, 60.98, 59.76, 56.49, 49.38, 45.48, 29.17, 27.79, 26.42, 23.74, 23.23, 11.29, 9.54, 3.94, 3.90. HRMS calculated for $C_{24}H_{31}NO_3$ $[M+H]^+$: 382.2381 (found); 382.2377 (calcd). HPLC purity: 98.29%.

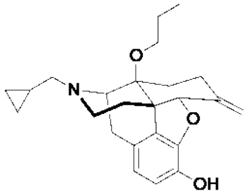

(4a*S*,7a*R*,12b*S*)-9-(benzyloxy)-3-(cyclopropylmethyl)-4a-(2,3-dihydroxypropoxy)-2,3,4,4a,5,6-hexahydro-1*H*-4,12-methanobenzofuro[3,2-*e*]isoquinolin-7(7a*H*)-one (41). To a solution of **22** (410 mg, 0.987 mmol) in DMF (8.0 mL) was added K_2CO_3 (273 mg, 1.974 mmol) and benzyl bromide (0.13 mL, 1.085 mmol). After stirring

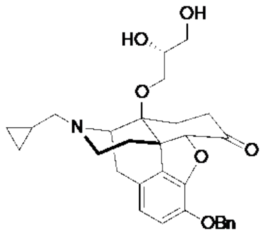

at room temperature for 16 h, the reaction mixture was poured into water (100 mL) and extracted with EtOAc (3 × 100 mL). The combined organic extracts were dried over Na_2SO_4, filtered, and concentrated. The resulting residue was purified by column chromatography (0-3% MeOH/CH_2Cl_2) to afford **41** (426 mg, 0.843 mmol, 85.4%) as a light-yellow oil. ^{1}H NMR (400 MHz, $CDCl_3$) δ 7.44 (m, 2H), 7.33 (m, 2H), 7.27 (m, 1H), 6.71 (d, *J* = 8.2 Hz, 1H), 6.55 (d, *J* = 8.2 Hz, 1H), 5.26 (d, *J* = 12.0 Hz, 1H), 5.20 (d, *J* = 12.0 Hz, 1H), 4.71 (s, 1H), 4.67 (s, 1H), 3.99 (m, 1H), 3.77 (dd, *J* = 11.4, 4.6 Hz, 1H), 3.69 – 3.53 (m, 3H), 3.15 (d, *J* = 18.7 Hz, 1H), 2.94 (m, 1H), 2.73 (m, 2H), 2.53 (dd, *J* = 12.5, 5.4 Hz, 1H), 2.41 (dd, *J* = 18.8, 5.5 Hz, 1H), 2.28 – 2.14 (m, 2H), 2.01 (m, 2H), 1.52 (m, 2H), 0.95 (m, 1H), 0.55 (m, 2H), 0.12 (m, 2H). ^{13}C NMR (100 MHz, $CDCl_3$) δ 207.70, 145.64, 142.04, 137.45, 128.47 (2C), 127.89, 127.84 (2C), 127.02, 125.70, 119.51, 118.24, 90.34, 76.13, 72.18, 70.60, 63.78, 63.44, 59.94, 55.81, 50.85, 44.15, 35.48, 29.09, 26.98, 23.11, 8.29, 5.02, 3.30.

3-(((4a*S*,7a*S*,12b*S*)-9-(benzyloxy)-3-(cyclopropylmethyl)-7-methylene-1,2,3,4,5,6,7,7a-octahydro-4a*H*-4,12-methanobenzofuro[3,2-*e*]isoquinolin-4a-yl)oxy)propane-1,2-diol (39c). To a suspension of

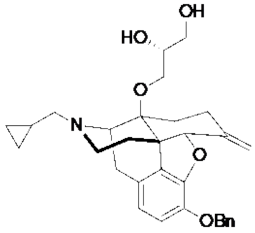

methyltriphenylphosphonium bromide (1.156 g, 3.236 mmol) in THF (4.0 mL) was added tBuOK (395 mg, 3.514 mmol) in portions over a minute. The resulting yellow suspension stirred at room temperature for 30 minutes, then a solution of **41** (420 mg, 0.831 mmol) in THF (2 mL) was added dropwise. The reaction stirred at room temperature for 16 h under a nitrogen atmosphere. The reaction mixture was poured into sat. aq. NH_4Cl (10 mL) and extracted with EtOAc (3 × 10 mL). The combined organic extracts were washed with water (8 mL), dried over Na_2SO_4, filtered, and concentrated. The resulting residue was purified by column chromatography (0-5% MeOH/CH_2Cl_2) to afford **39c** (72 mg, 0.143 mmol, 17.2%) as a yellow foam. ^{1}HMR (400 MHz, $CDCl_3$) δ 7.43 (m, 2H), 7.35 (m, 2H), 7.30 (m, 1H), 6.74 (d, *J* = 8.2 Hz, 1H), 6.54 (d, *J* = 8.2 Hz, 1H), 5.33 (m, 1H), 5.19 (s, 2H), 5.05 (m, 1H), 4.88 (m, 1H), 3.96 (m, 1H), 3.78 (dd, *J* = 11.5, 4.3 Hz, 1H), 3.62 – 3.53 (m, 3H), 3.50 (d, *J* = 5.2 Hz, 1H), 3.12 (d, *J* = 18.5 Hz, 1H), 2.98 (dd, *J* = 12.0, 4.9 Hz, 1H), 2.67 – 2.37 (m, 3H), 2.19 (dd, *J* = 12.5, 7.6 Hz, 1H), 2.12 – 1.96 (m, 2H), 1.77 (m, 1H), 1.49 (dd, *J* = 12.5, 3.9 Hz, 1H), 1.25 (m, 2H), 0.96 (m, 1H), 0.54 (m, 2H), 0.14 (m, 2H). ^{13}C NMR (100 MHz, $CDCl_3$) δ 145.42, 144.92, 142.55, 137.62, 133.18, 131.31, 128.58 (2C), 127.96, 127.68 (2C), 126.00, 118.70, 117.16, 112.27, 89.25, 72.04, 70.79, 63.63, 63.39, 60.03, 56.51, 48.67, 45.04, 28.77, 27.64, 27.55, 23.33, 8.28, 5.16, 3.31.

(R)-3-(((4R,4aS,7aS,12bS)-3-(cyclopropylmethyl)-9-hydroxy-7-methylene-1,2,3,4,5,6,7,7a-octahydro-4aH-4,12-methanobenzofuro[3,2-e]isoquinolin-4a-yl)oxy)propane-1,2-diol (30). A solution of **29c** (47 mg, 0.093

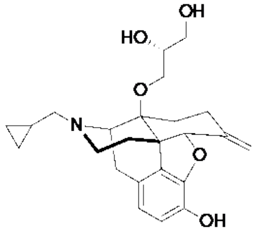

mmol) in MeOH (2.0 mL) and conc. HCl (1.5 mL) was refluxed for 4 hours. The reaction was cooled to room temperature, diluted with water, and treated with 5 N NaOH / solid NH_4Cl until pH = 8. The product was then extracted using EtOAc (3 × 10 mL). The combined organic extracts were dried over Na_2SO_4, filtered, and concentrated. The resulting residue was purified by column chromatography (0-10% MeOH/CH_2Cl_2) to afford **30** (25 mg, 0.060 mmol, 64.8%) as a light brown solid. ^{1}H NMR (400 MHz, MeOD) δ 6.60 (d, *J* = 8.1 Hz, 1H), 6.53 (d, *J* = 8.1 Hz, 1H), 5.31 (q, *J* = 2.0 Hz, 1H), 4.98 (q, *J* = 1.9 Hz, 1H), 4.84 (q, *J* = 2.2 Hz, 1H), 3.92 (m, 1H), 3.71 (d, *J* = 5.4 Hz, 1H), 3.67 – 3.55 (m, 4H), 3.21 (s, 1H), 2.95 (dd, *J* = 12.3, 4.9 Hz, 1H), 2.67 – 2.42 (m, 4H), 2.35 (dd, *J* = 12.7, 7.3 Hz, 1H), 2.13 (m, 2H), 1.86 (dt, *J* = 13.9, 3.4 Hz, 1H), 1.45 (m, 1H), 1.21 (m, 1H), 1.00 (m, 1H), 0.56 (m, 2H), 0.22 (m, 2H). ^{13}C NMR (100 MHz, MeOD) δ 146.81, 144.84, 141.54, 134.96, 131.67, 124.96, 119.83, 118.25, 111.93, 90.02, 77.93, 72.29, 64.05, 63.63, 60.50, 57.32, 49.92, 46.48, 29.74, 28.41, 23.98, 8.57, 5.03, 4.08. HRMS calculated for $C_{24}H_{31}NO_5$ $[M+H]^+$: 414.2274 (found); 414.2275 (calcd). HPLC purity: 97.52%.

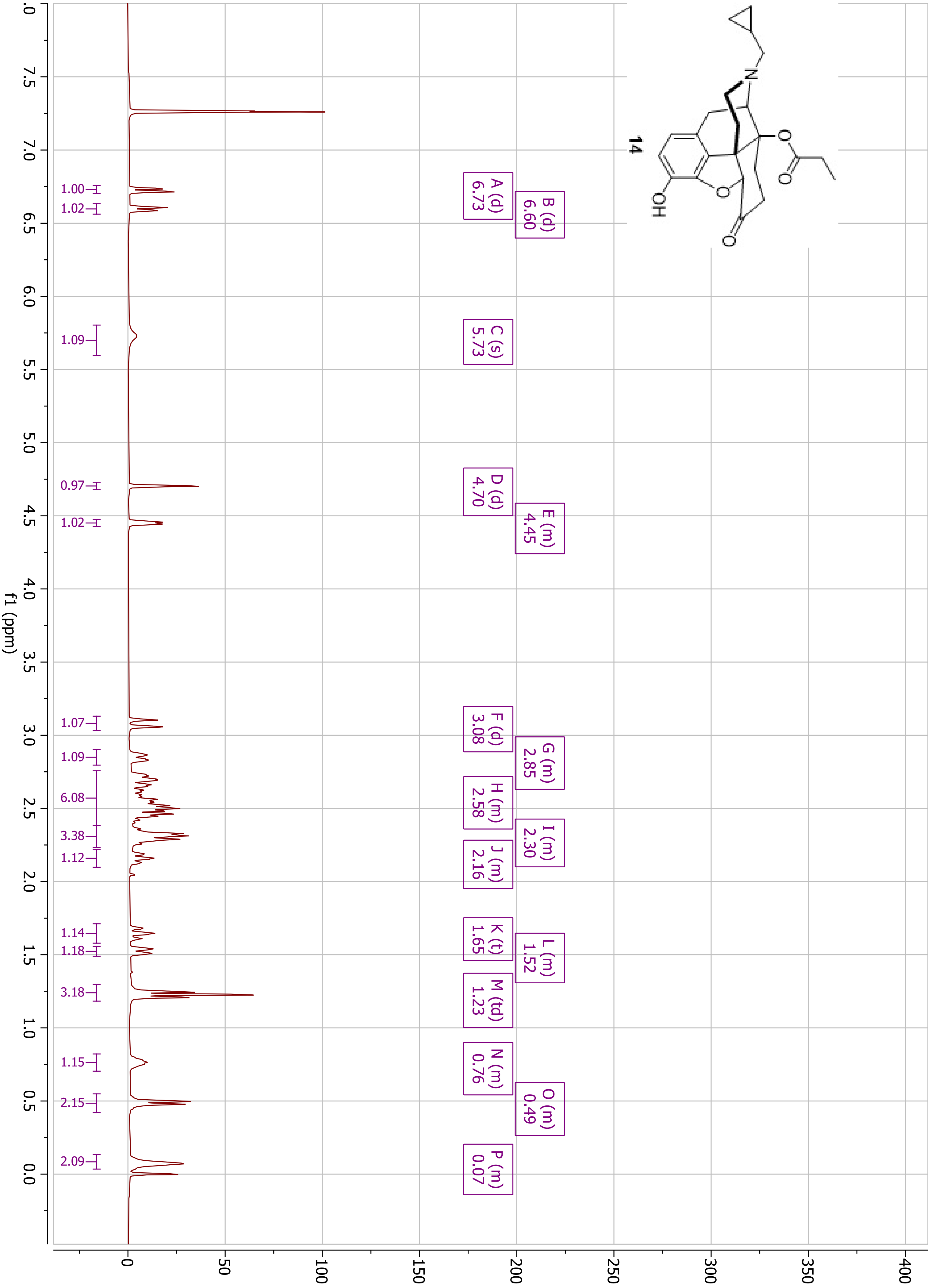

14
OH
O
N
A (d) 6.73
B (d) 6.60
C (s) 5.73
D (d) 4.70
E (m) 4.45
F (d) 3.08
G (m) 2.85
H (m) 2.58
I (m) 2.30
J (m) 2.16
K (t) 1.65
L (m) 1.52
M (td) 1.23
N (m) 0.76
O (m) 0.49
P (m) 0.07
1.00
1.02
1.09
0.97
1.02
1.07
1.09
6.08
3.38
1.12
1.14
1.18
3.18
1.15
2.15
2.09
f1 (ppm)

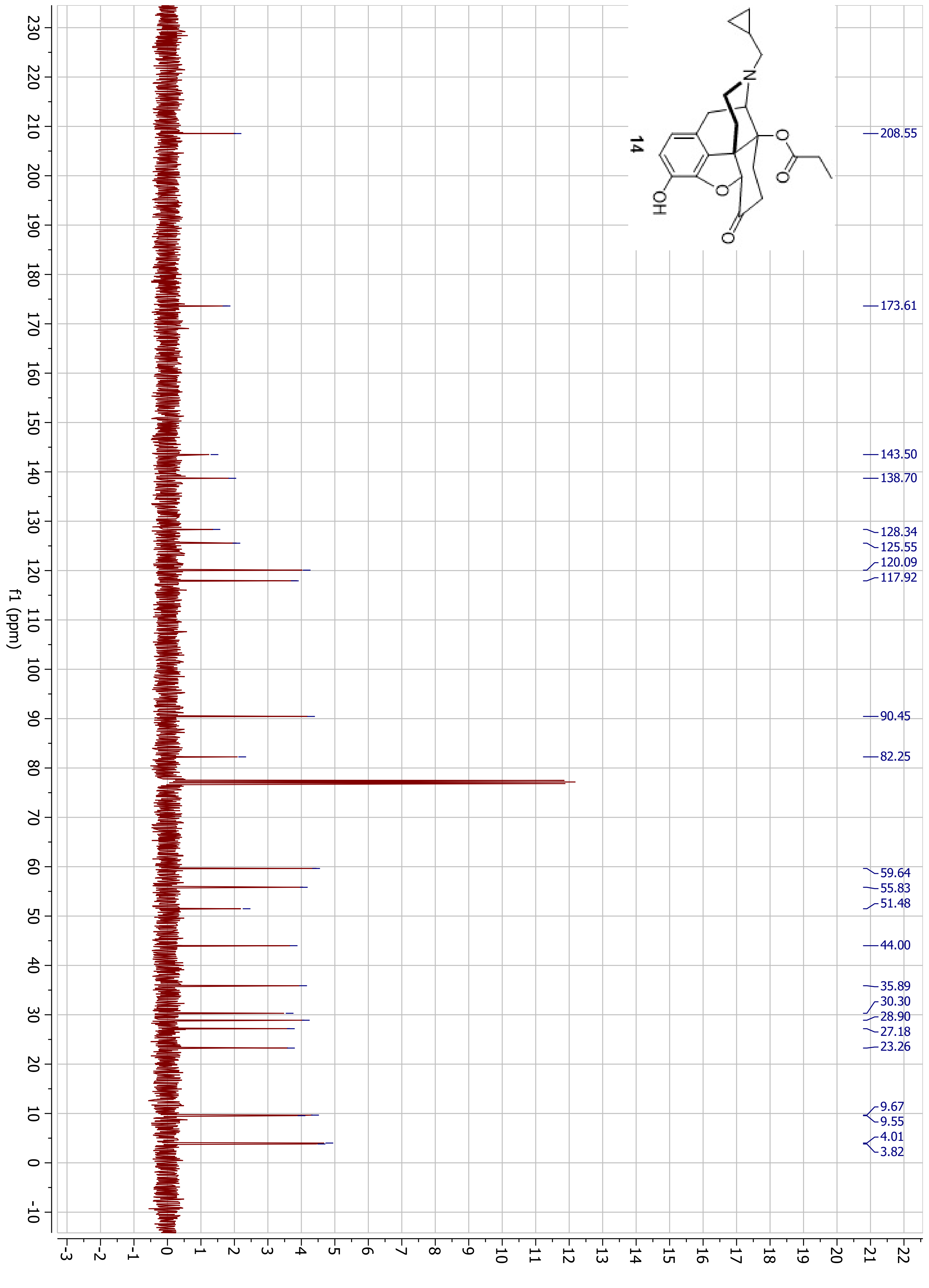
14
OH
O
N
208.55
173.61
143.50
138.70
128.34
125.55
120.09
117.92
90.45
82.25
59.64
55.83
51.48
44.00
35.89
30.30
28.90
27.18
23.26
9.67
9.55
4.01
3.82
f1 (ppm)

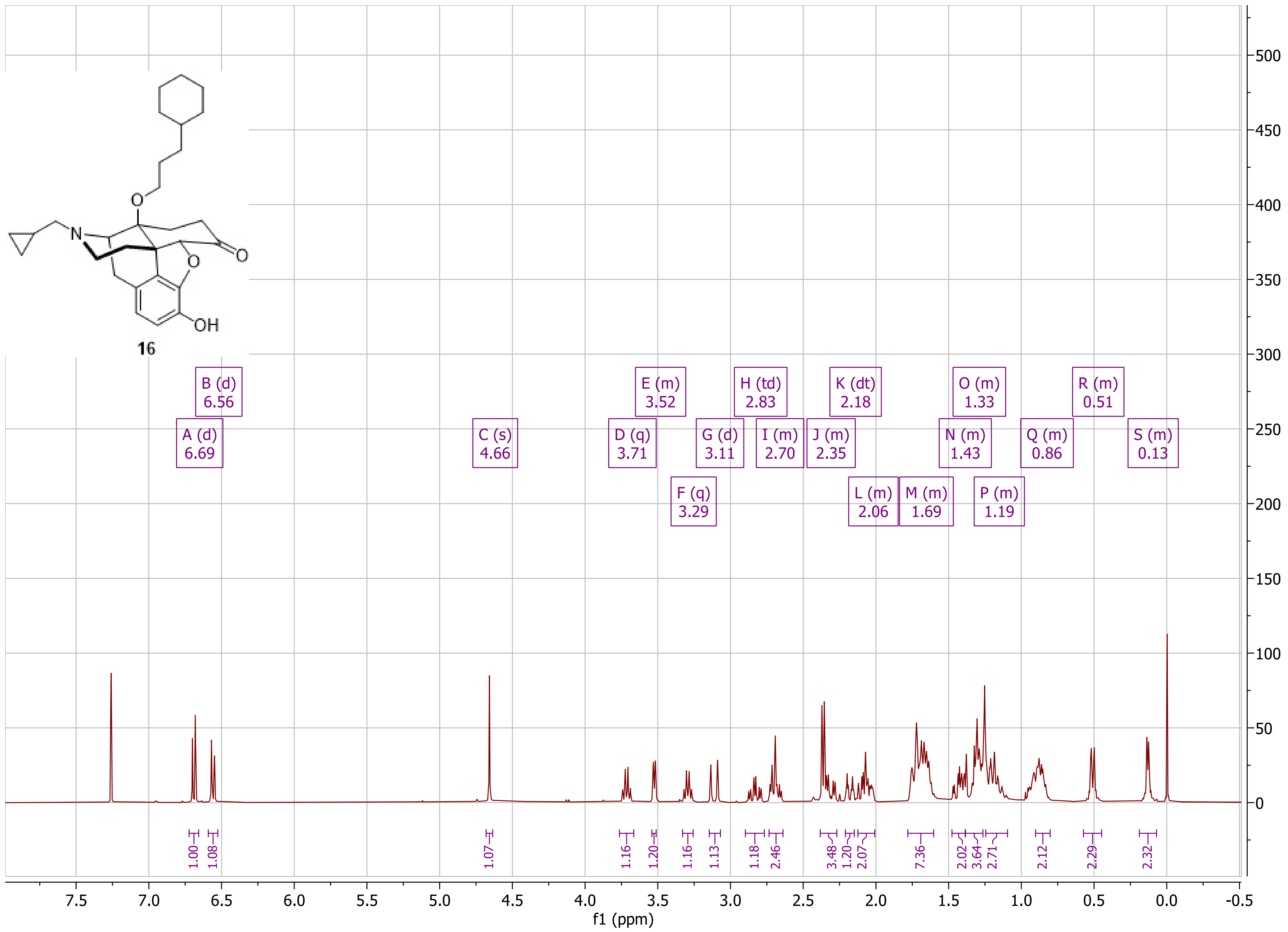
OH
O
O
N
O
16
A (d)
6.69
B (d)
6.56
C (s)
4.66
D (q)
3.71
E (m)
3.52
F (q)
3.29
G (d)
3.11
H (td)
2.83
I (m)
2.70
J (m)
2.35
K (dt)
2.18
L (m)
2.06
M (m)
1.69
N (m)
1.43
O (m)
1.33
P (m)
1.19
Q (m)
0.86
R (m)
0.51
S (m)
0.13
1.00
1.08
1.07
1.16
1.20
1.16
1.13
1.18
2.46
3.48
1.20
2.07
7.36
2.02
3.64
2.71
2.12
2.29
2.32
f1 (ppm)

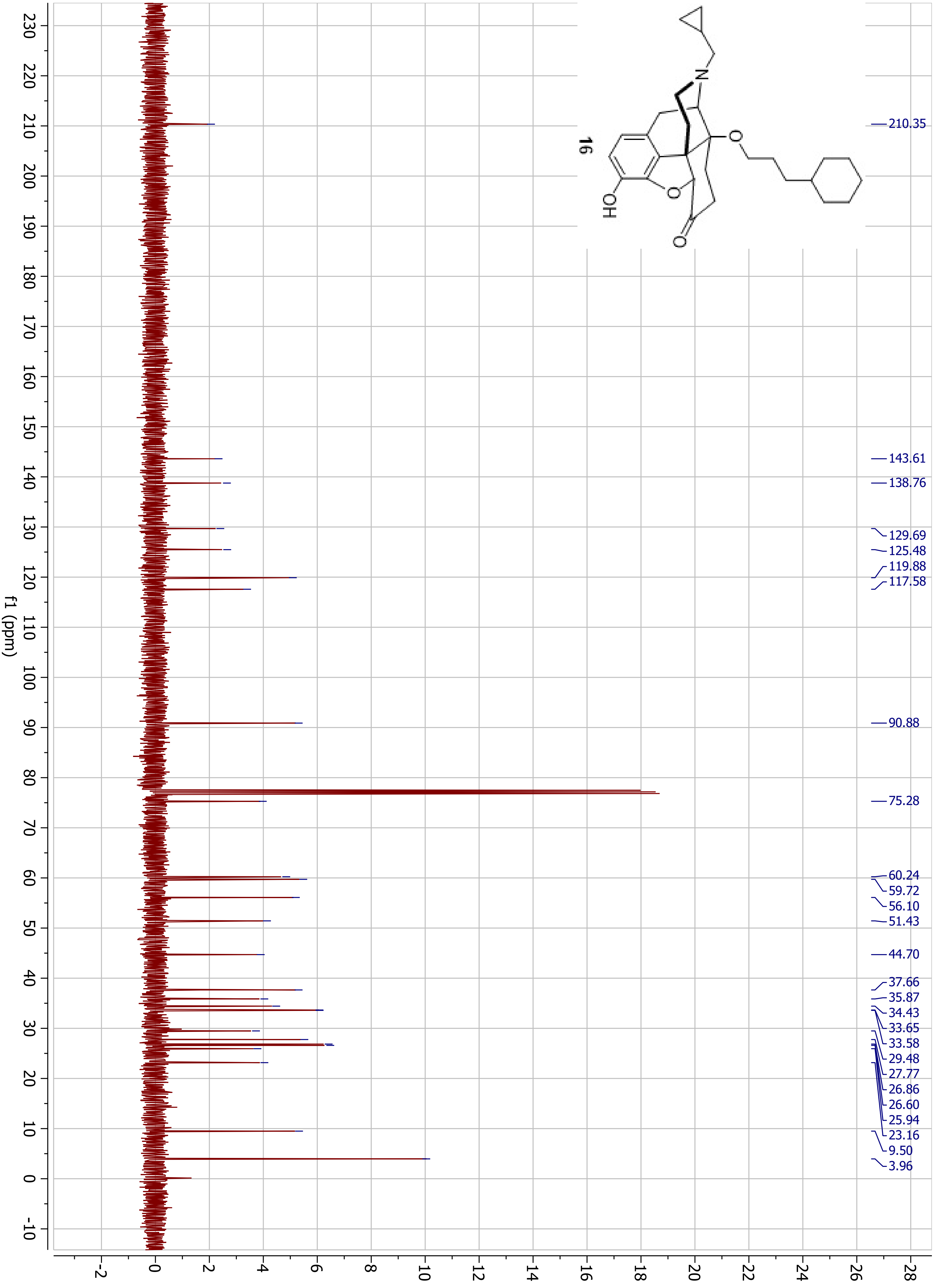
16
OH
210.35
143.61
138.76
129.69
125.48
119.88
117.58
90.88
75.28
60.24
59.72
56.10
51.43
44.70
37.66
35.87
34.43
33.65
33.58
29.48
27.77
26.86
26.60
25.94
23.16
9.50
3.96
f1 (ppm)

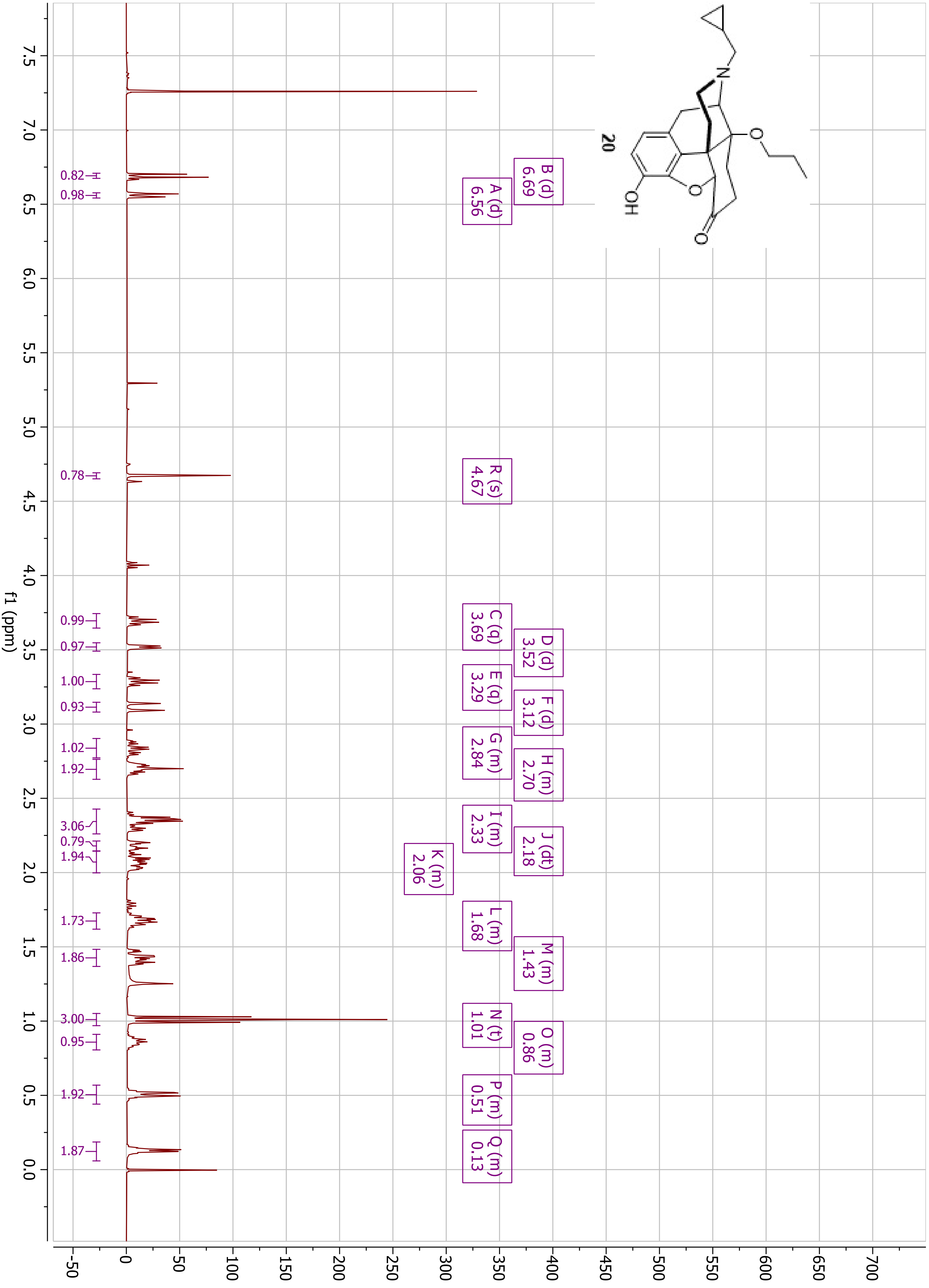
20
OH
B (d) 6.69
A (d) 6.56
R (s) 4.67
C (q) 3.69
D (d) 3.52
E (q) 3.29
F (d) 3.12
G (m) 2.84
H (m) 2.70
I (m) 2.33
J (dt) 2.18
K (m) 2.06
L (m) 1.68
M (m) 1.43
N (t) 1.01
O (m) 0.86
P (m) 0.51
Q (m) 0.13
0.82
0.98
0.78
0.99
0.97
1.00
0.93
1.02
1.92
3.06
0.79
1.94
1.73
1.86
3.00
0.95
1.92
1.87
f1 (ppm)

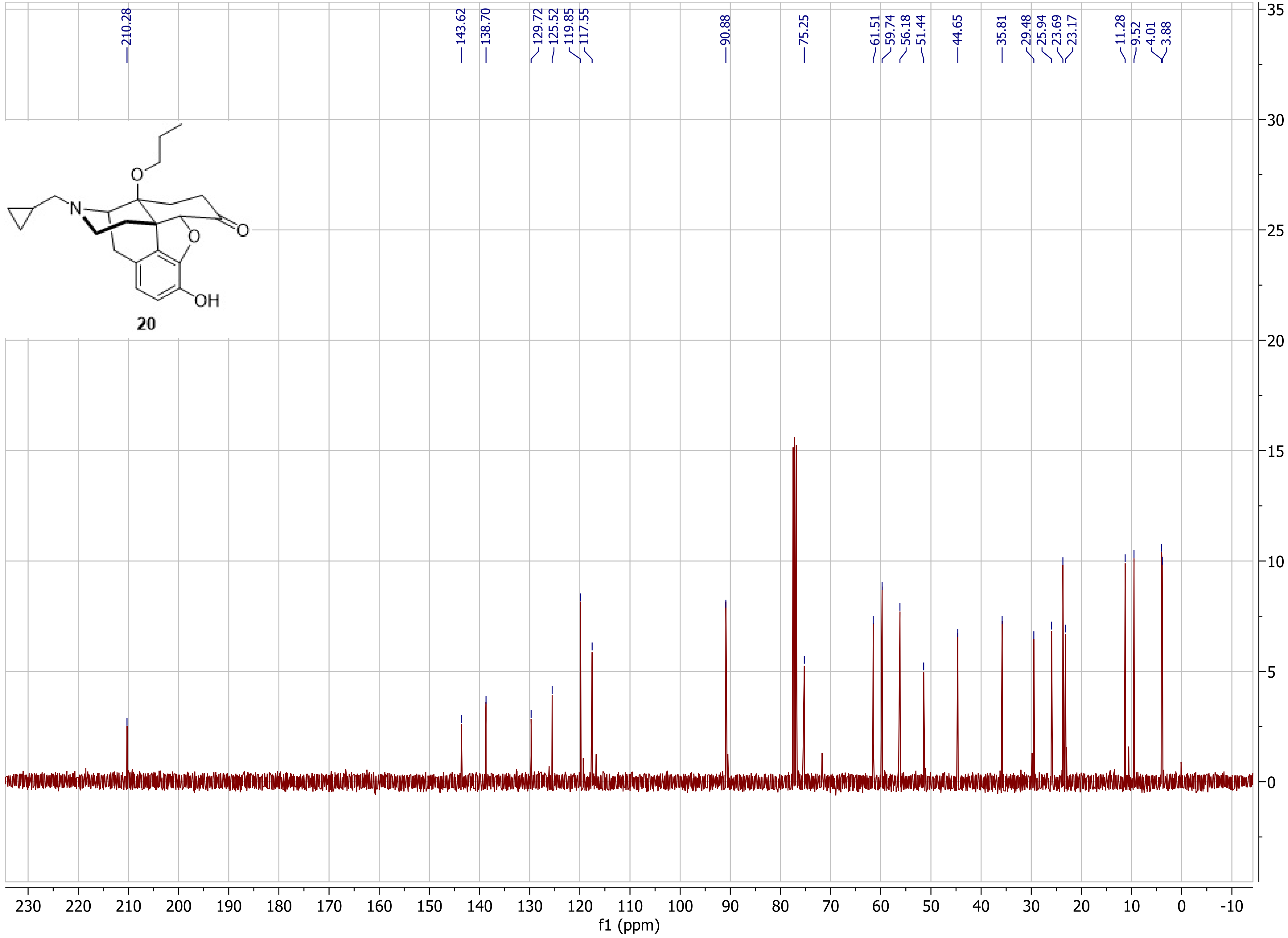
20
OH
O
N
210.28
143.62
138.70
129.72
125.52
119.85
117.55
90.88
75.25
61.51
59.74
56.18
51.44
44.65
35.81
29.48
25.94
23.69
23.17
11.28
9.52
4.01
3.88
f1 (ppm)

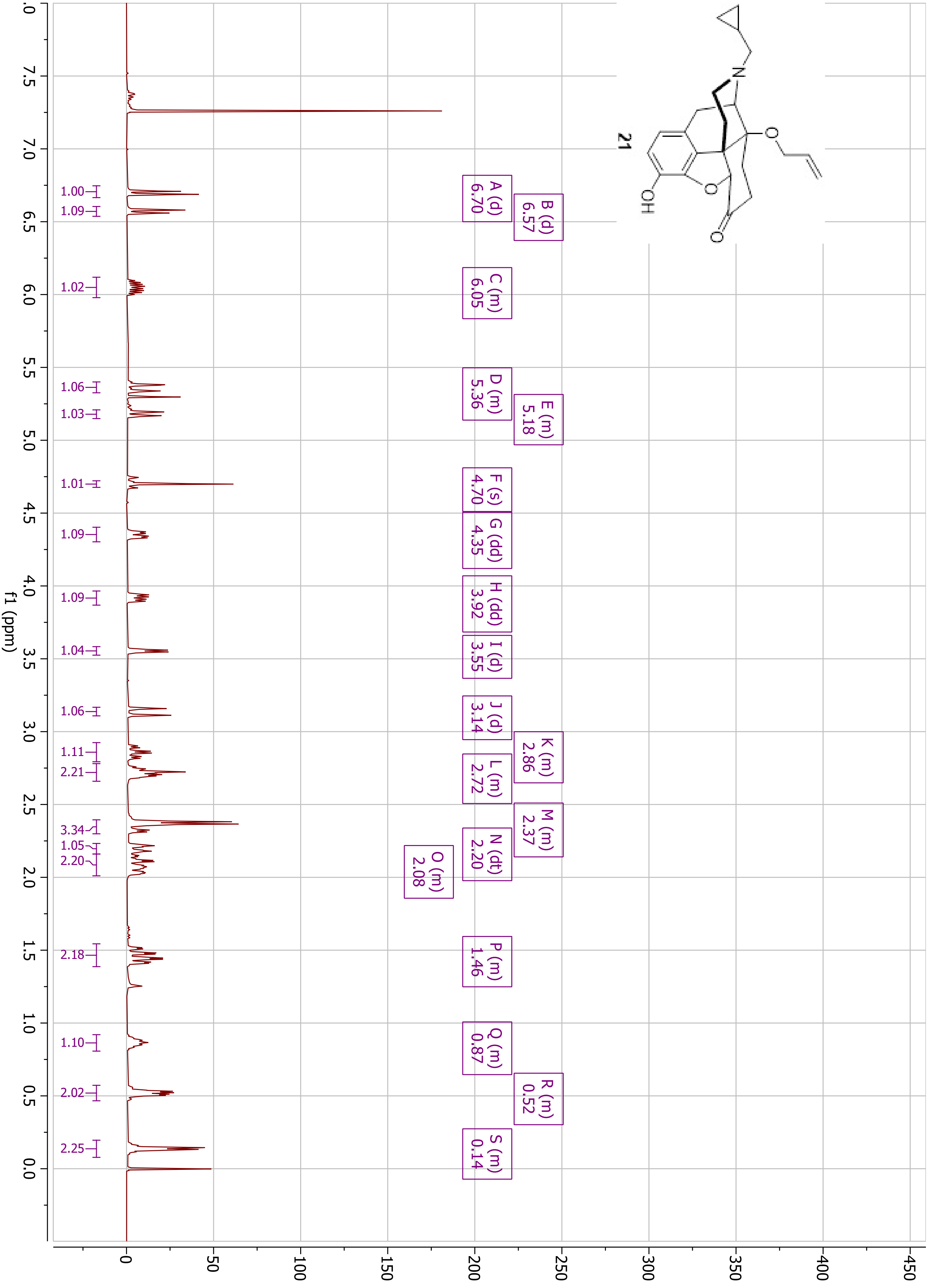

21
OH
O
N
A (d) 6.70
B (d) 6.57
C (m) 6.05
D (m) 5.36
E (m) 5.18
F (s) 4.70
G (dd) 4.35
H (dd) 3.92
I (d) 3.55
J (d) 3.14
K (m) 2.86
L (m) 2.72
M (m) 2.37
N (dt) 2.20
O (m) 2.08
P (m) 1.46
Q (m) 0.87
R (m) 0.52
S (m) 0.14
1.00
1.09
1.02
1.06
1.03
1.01
1.09
1.09
1.04
1.06
1.11
2.21
3.34
1.05
2.20
2.18
1.10
2.02
2.25
f1 (ppm)

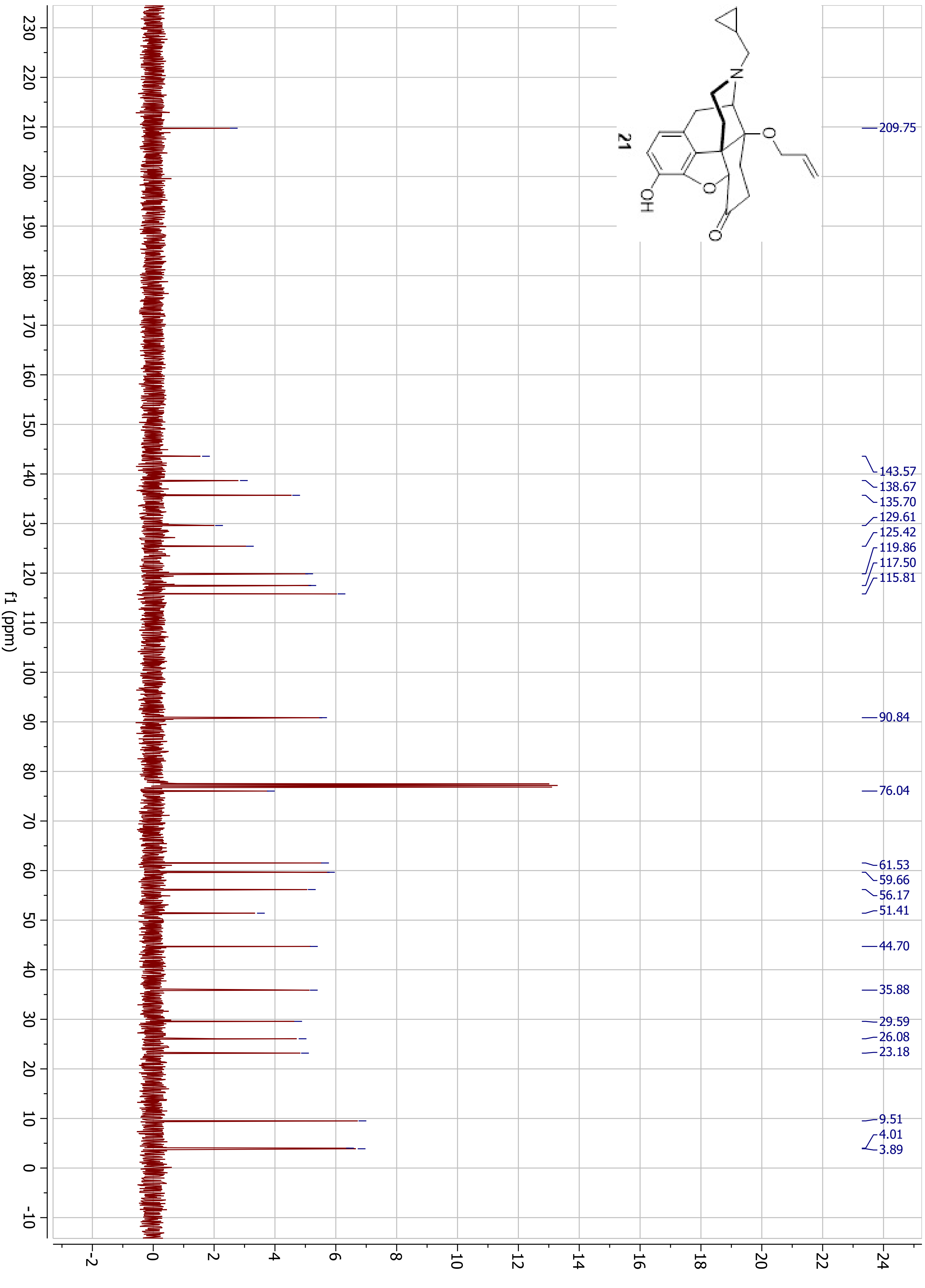
21
OH
N
O
209.75
143.57
138.67
135.70
129.61
125.42
119.86
117.50
115.81
90.84
76.04
61.53
59.66
56.17
51.41
44.70
35.88
29.59
26.08
23.18
9.51
4.01
3.89
f1 (ppm)

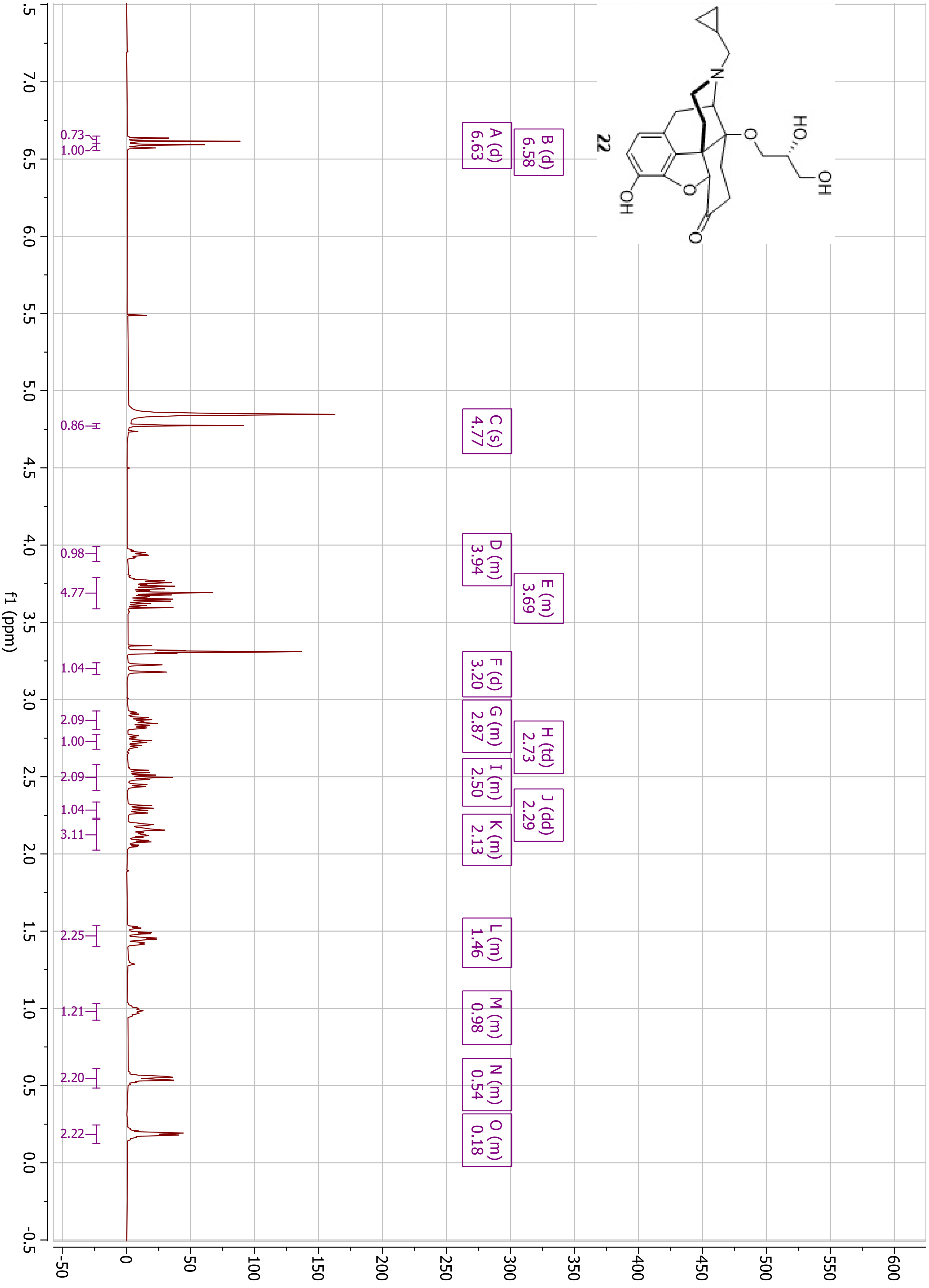
22
OH
HO
OH
O
O
O
N
A (d) 6.63
B (d) 6.58
C (s) 4.77
D (m) 3.94
E (m) 3.69
F (d) 3.20
G (m) 2.87
H (td) 2.73
I (m) 2.50
J (dd) 2.29
K (m) 2.13
L (m) 1.46
M (m) 0.98
N (m) 0.54
O (m) 0.18
0.73
1.00
0.86
0.98
4.77
1.04
2.09
1.00
2.09
1.04
3.11
2.25
1.21
2.20
2.22
f1 (ppm)

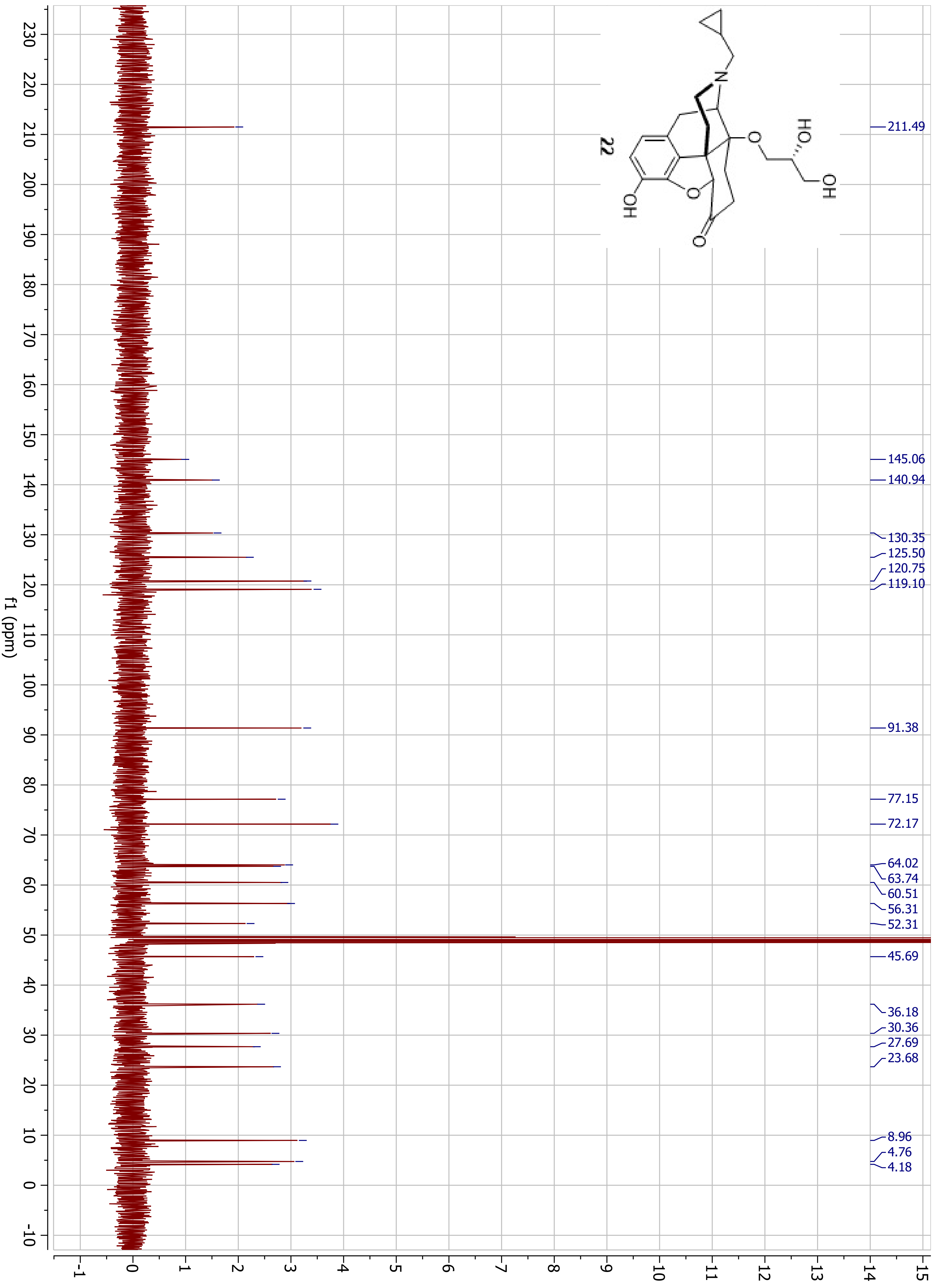
22
211.49
145.06
140.94
130.35
125.50
120.75
119.10
91.38
77.15
72.17
64.02
63.74
60.51
56.31
52.31
45.69
36.18
30.36
27.69
23.68
8.96
4.76
4.18
f1 (ppm)

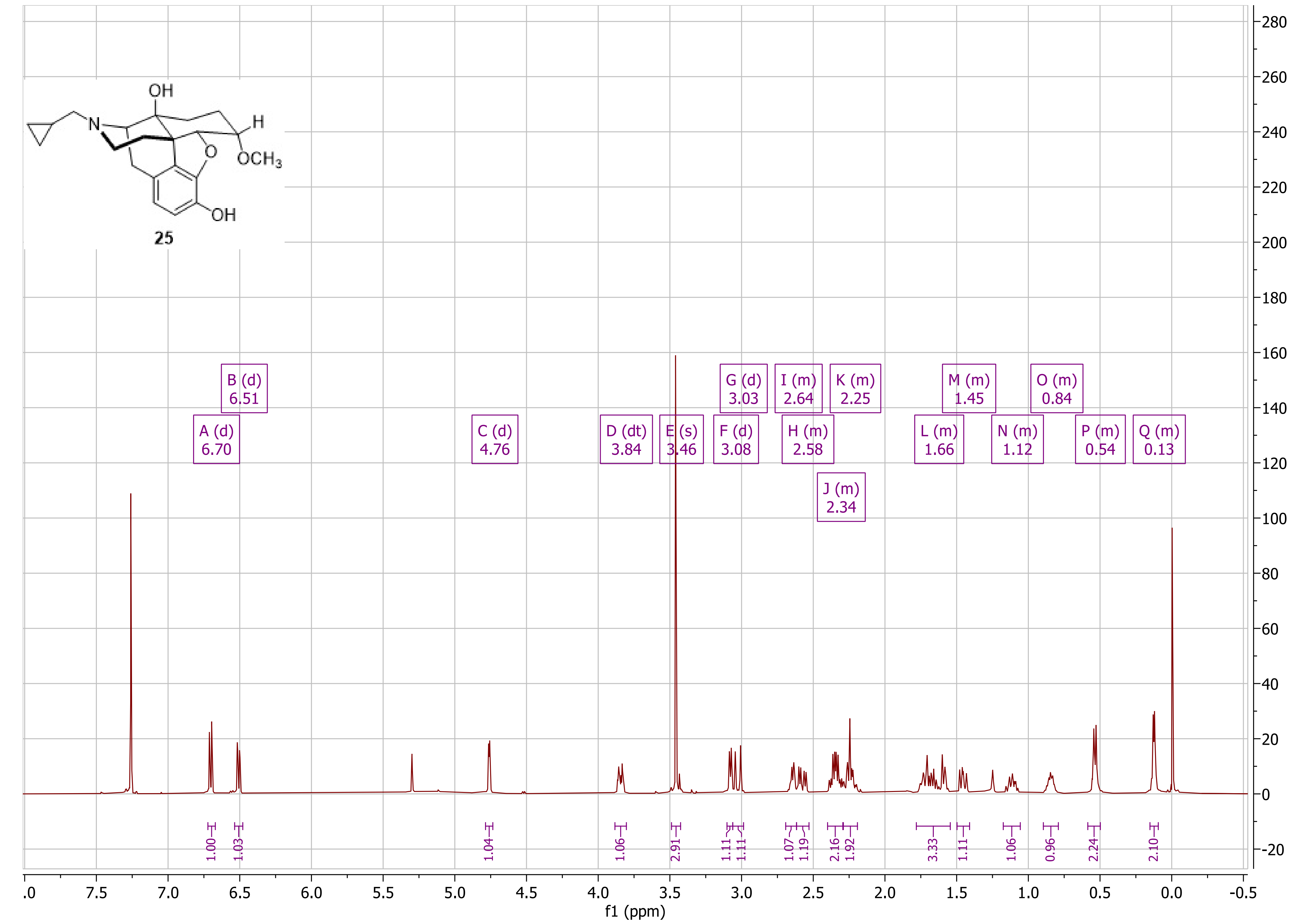

OH
N
H
O
OCH3
OH
25
A (d) 6.70
B (d) 6.51
C (d) 4.76
D (dt) 3.84
E (s) 3.46
F (d) 3.08
G (d) 3.03
H (m) 2.58
I (m) 2.64
J (m) 2.34
K (m) 2.25
L (m) 1.66
M (m) 1.45
N (m) 1.12
O (m) 0.84
P (m) 0.54
Q (m) 0.13
1.00
1.03
1.04
1.06
2.91
1.11
1.11
1.07
1.19
2.16
1.92
3.33
1.11
1.06
0.96
2.24
2.10
f1 (ppm)

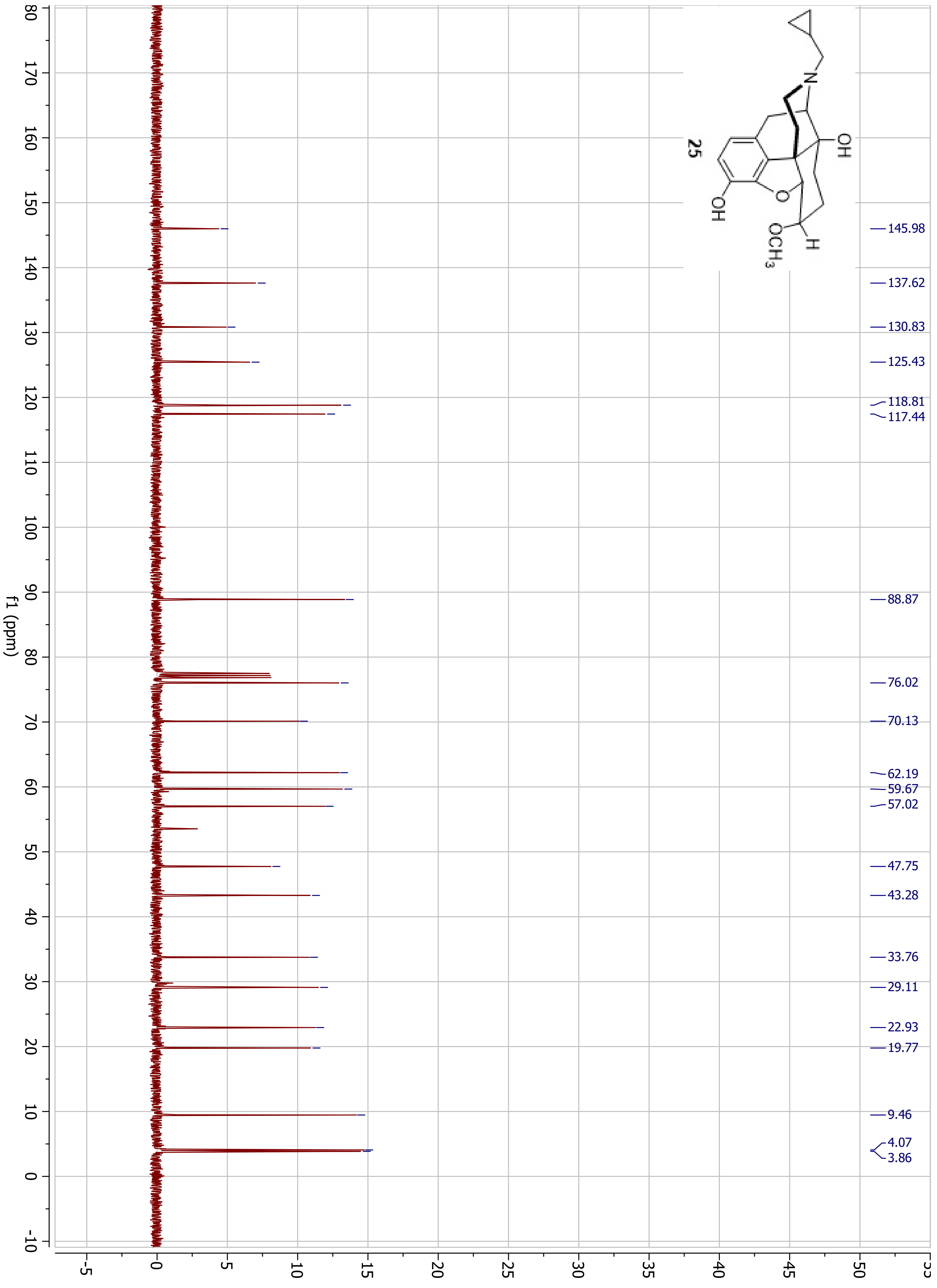

25
OH
OH
O
OCH3
H
N
145.98
137.62
130.83
125.43
118.81
117.44
88.87
76.02
70.13
62.19
59.67
57.02
47.75
43.28
33.76
29.11
22.93
19.77
9.46
4.07
3.86
f1 (ppm)

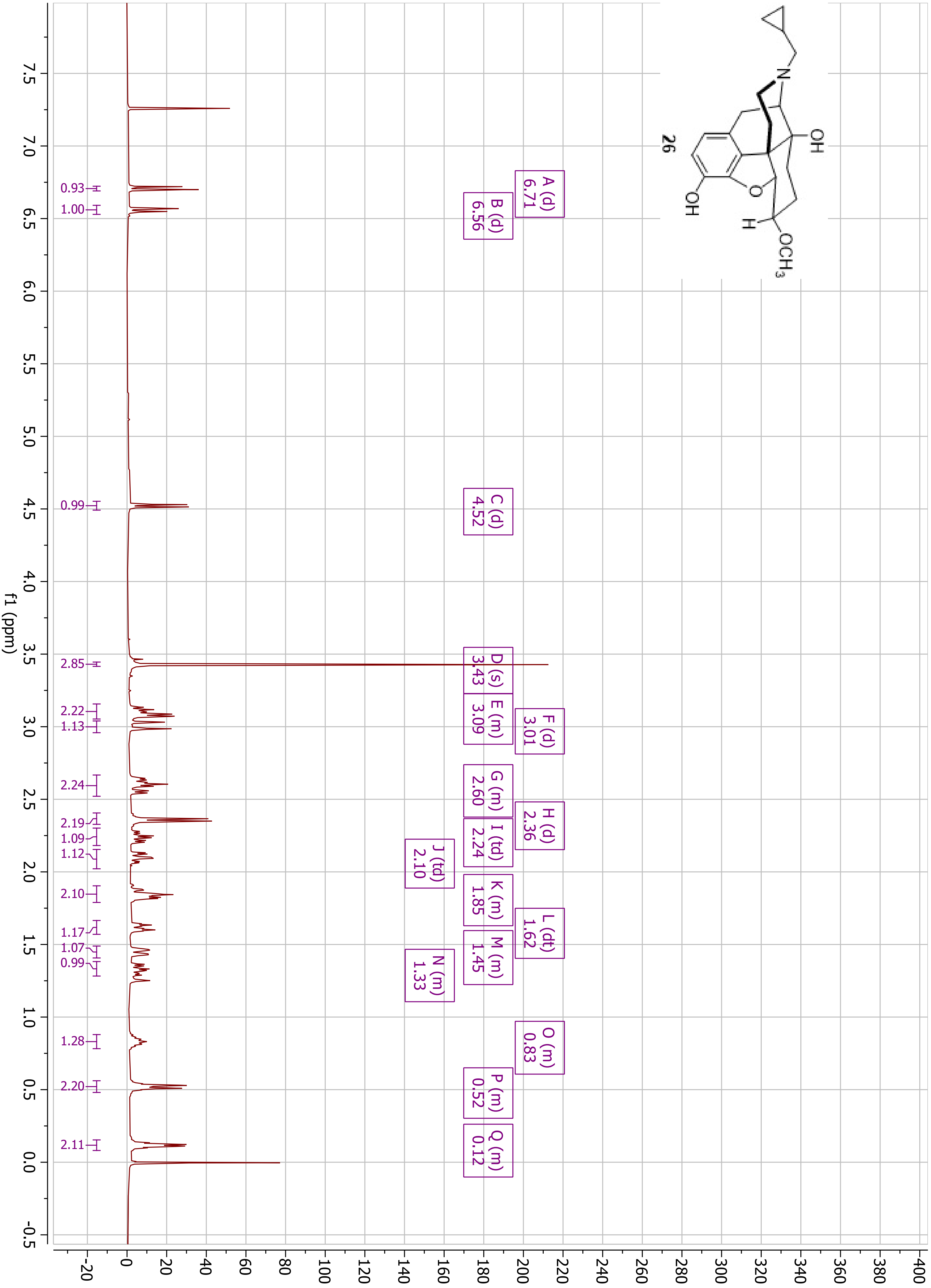

26
OH
OH
O
H
OCH$_3$
N
A (d) 6.71
B (d) 6.56
C (d) 4.52
D (s) 3.43
E (m) 3.09
F (d) 3.01
G (m) 2.60
H (d) 2.36
I (td) 2.24
J (td) 2.10
K (m) 1.85
L (dt) 1.62
M (m) 1.45
N (m) 1.33
O (m) 0.83
P (m) 0.52
Q (m) 0.12
0.93
1.00
0.99
2.85
2.22
1.13
2.24
2.19
1.09
1.12
2.10
1.17
1.07
0.99
1.28
2.20
2.11
f1 (ppm)

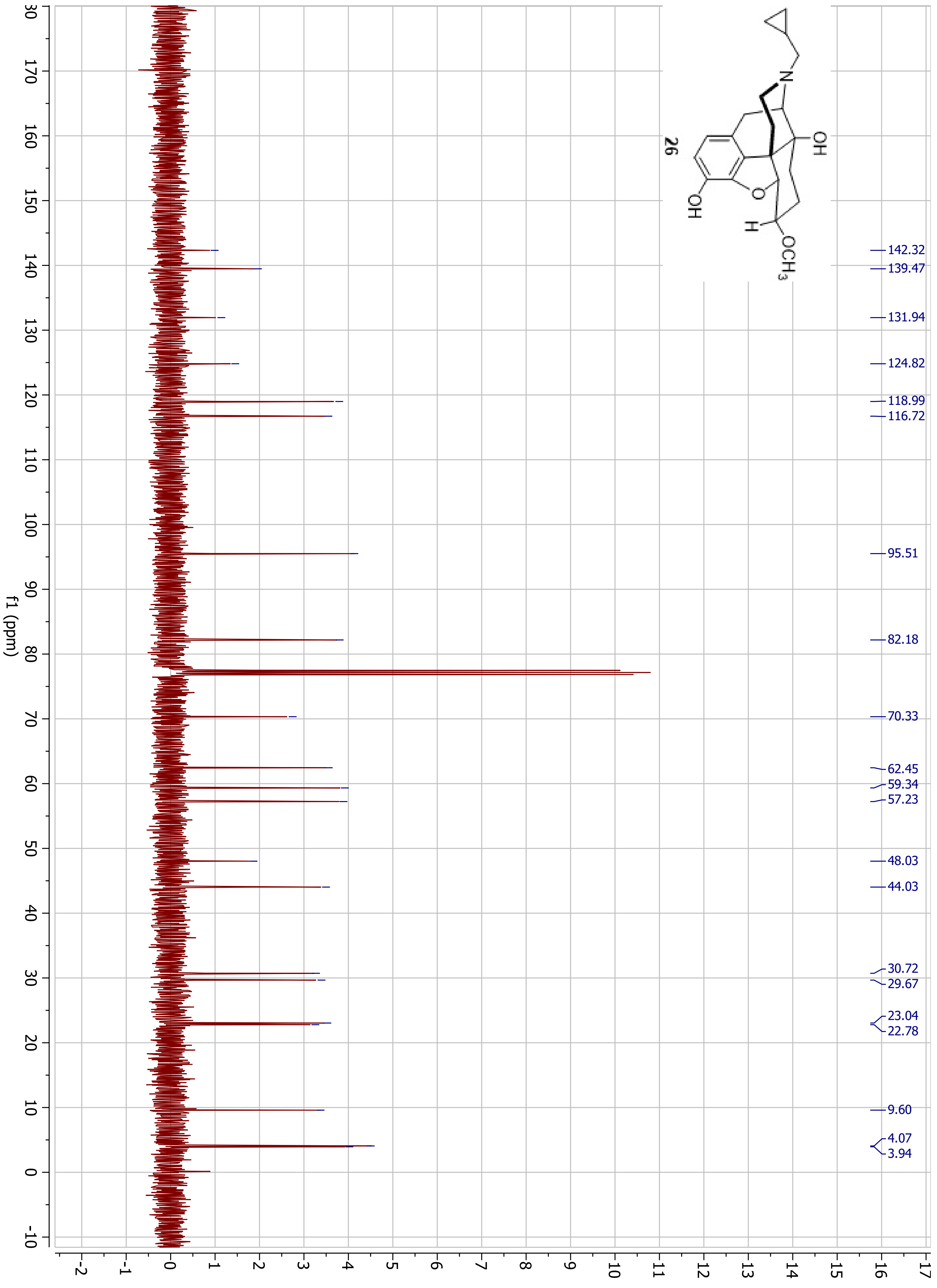
26
f1 (ppm)
142.32
139.47
131.94
124.82
118.99
116.72
95.51
82.18
70.33
62.45
59.34
57.23
48.03
44.03
30.72
29.67
23.04
22.78
9.60
4.07
3.94

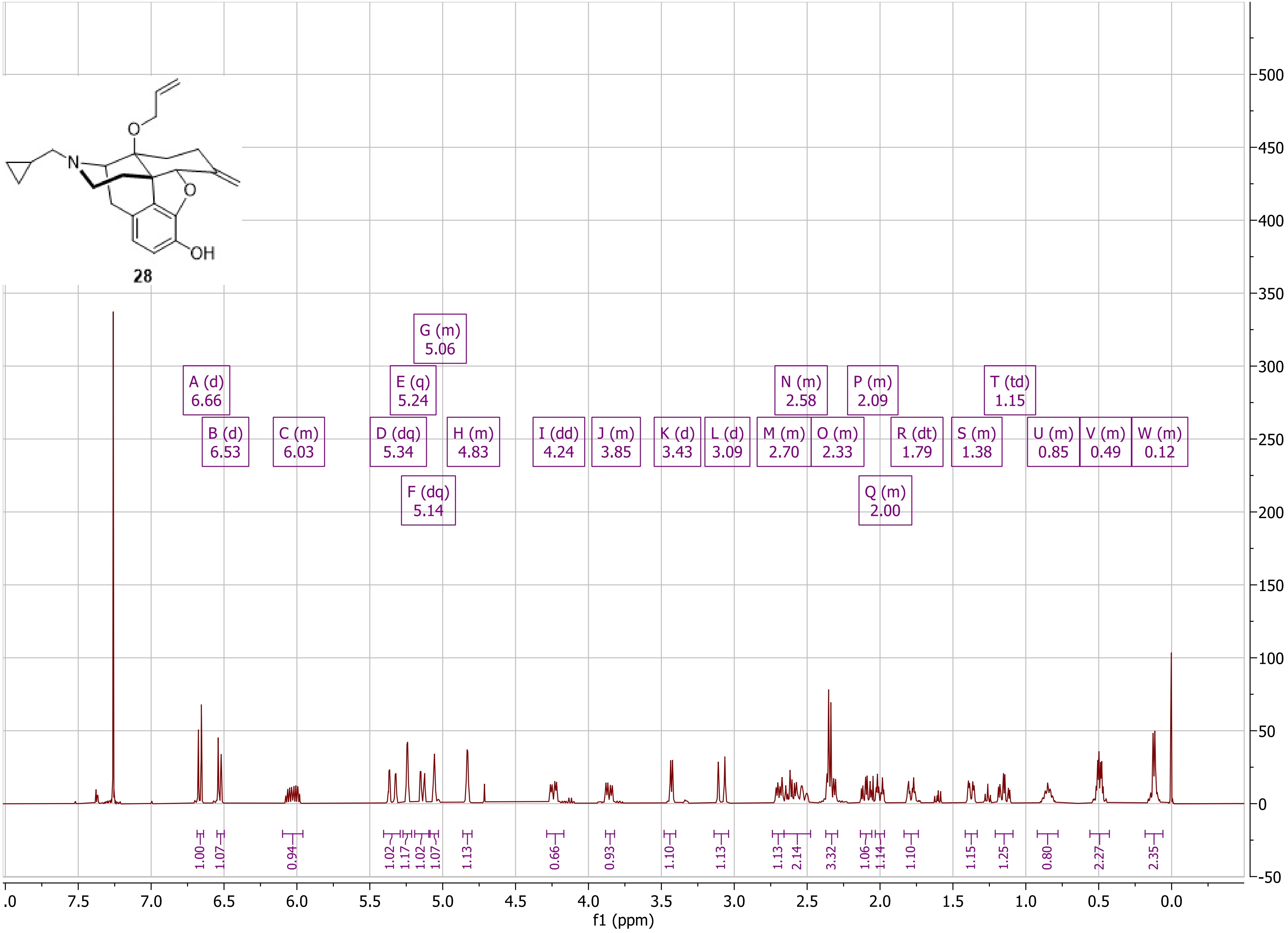
28
OH
A (d) 6.66
B (d) 6.53
C (m) 6.03
D (dq) 5.34
E (q) 5.24
F (dq) 5.14
G (m) 5.06
H (m) 4.83
I (dd) 4.24
J (m) 3.85
K (d) 3.43
L (d) 3.09
M (m) 2.70
N (m) 2.58
O (m) 2.33
P (m) 2.09
Q (m) 2.00
R (dt) 1.79
S (m) 1.38
T (td) 1.15
U (m) 0.85
V (m) 0.49
W (m) 0.12
1.00
1.07
0.94
1.02
1.17
1.02
1.07
1.13
0.66
0.93
1.10
1.13
1.13
2.14
3.32
1.06
1.14
1.10
1.15
1.25
0.80
2.27
2.35
f1 (ppm)

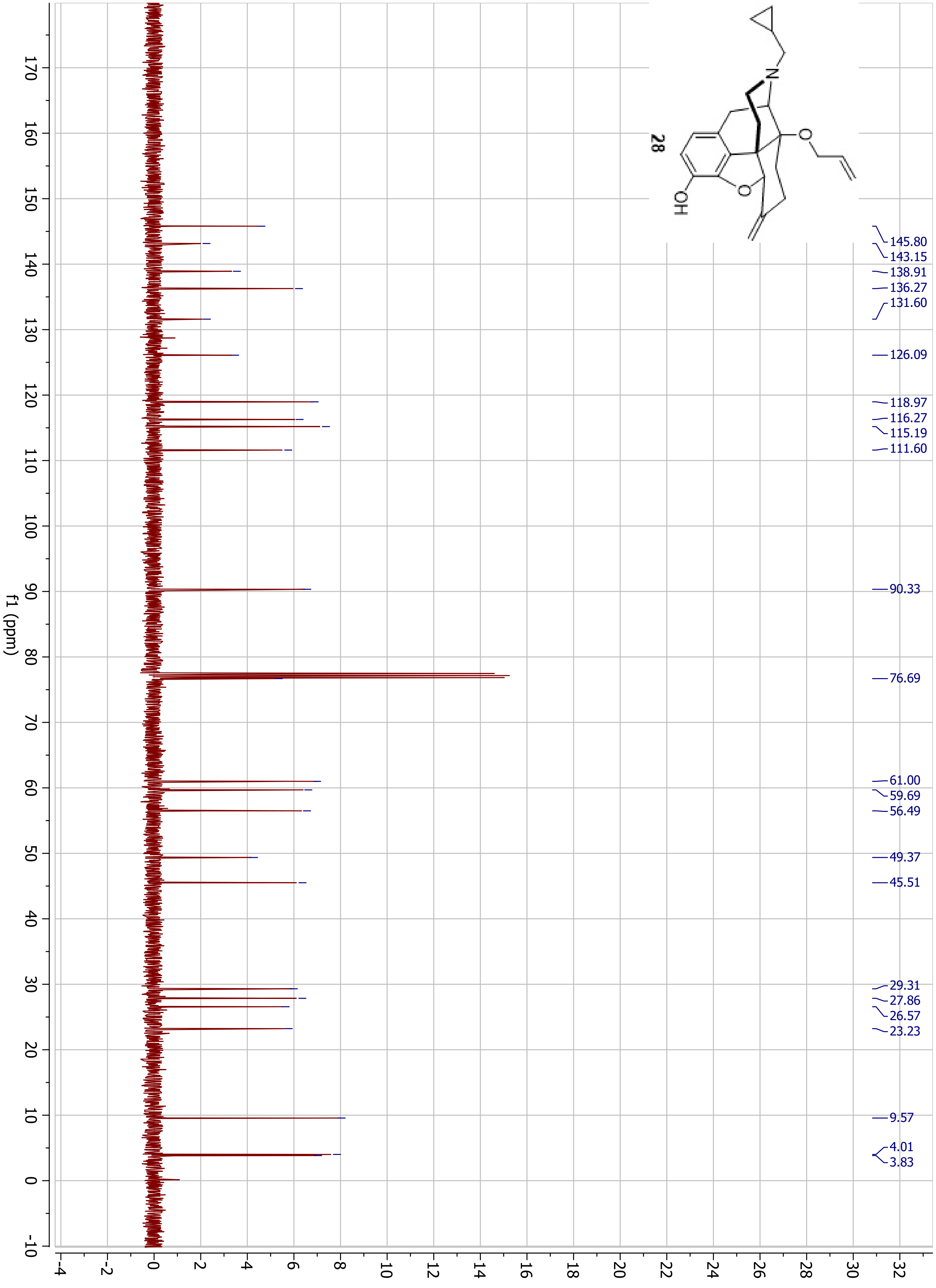
28
OH
O
N
145.80
143.15
138.91
136.27
131.60
126.09
118.97
116.27
115.19
111.60
90.33
76.69
61.00
59.69
56.49
49.37
45.51
29.31
27.86
26.57
23.23
9.57
4.01
3.83
f1 (ppm)

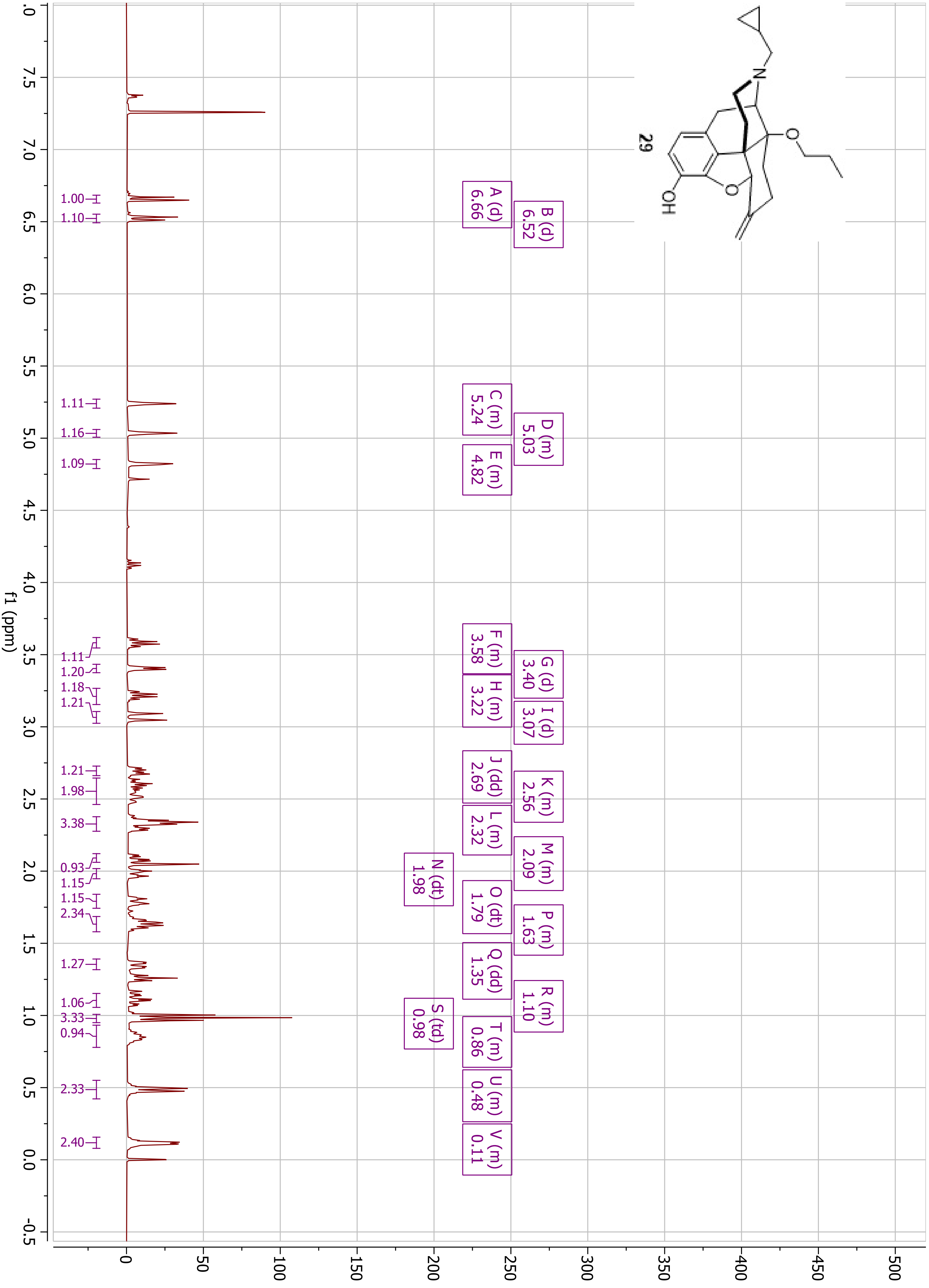
29
OH
A (d) 6.66
B (d) 6.52
C (m) 5.24
D (m) 5.03
E (m) 4.82
F (m) 3.58
G (d) 3.40
H (m) 3.22
I (d) 3.07
J (dd) 2.69
K (m) 2.56
L (m) 2.32
M (m) 2.09
N (dt) 1.98
O (dt) 1.79
P (m) 1.63
Q (dd) 1.35
R (m) 1.10
S (td) 0.98
T (m) 0.86
U (m) 0.48
V (m) 0.11
1.00
1.10
1.11
1.16
1.09
1.11
1.20
1.18
1.21
1.21
1.98
3.38
0.93
1.15
1.15
2.34
1.27
1.06
3.33
0.94
2.33
2.40
f1 (ppm)

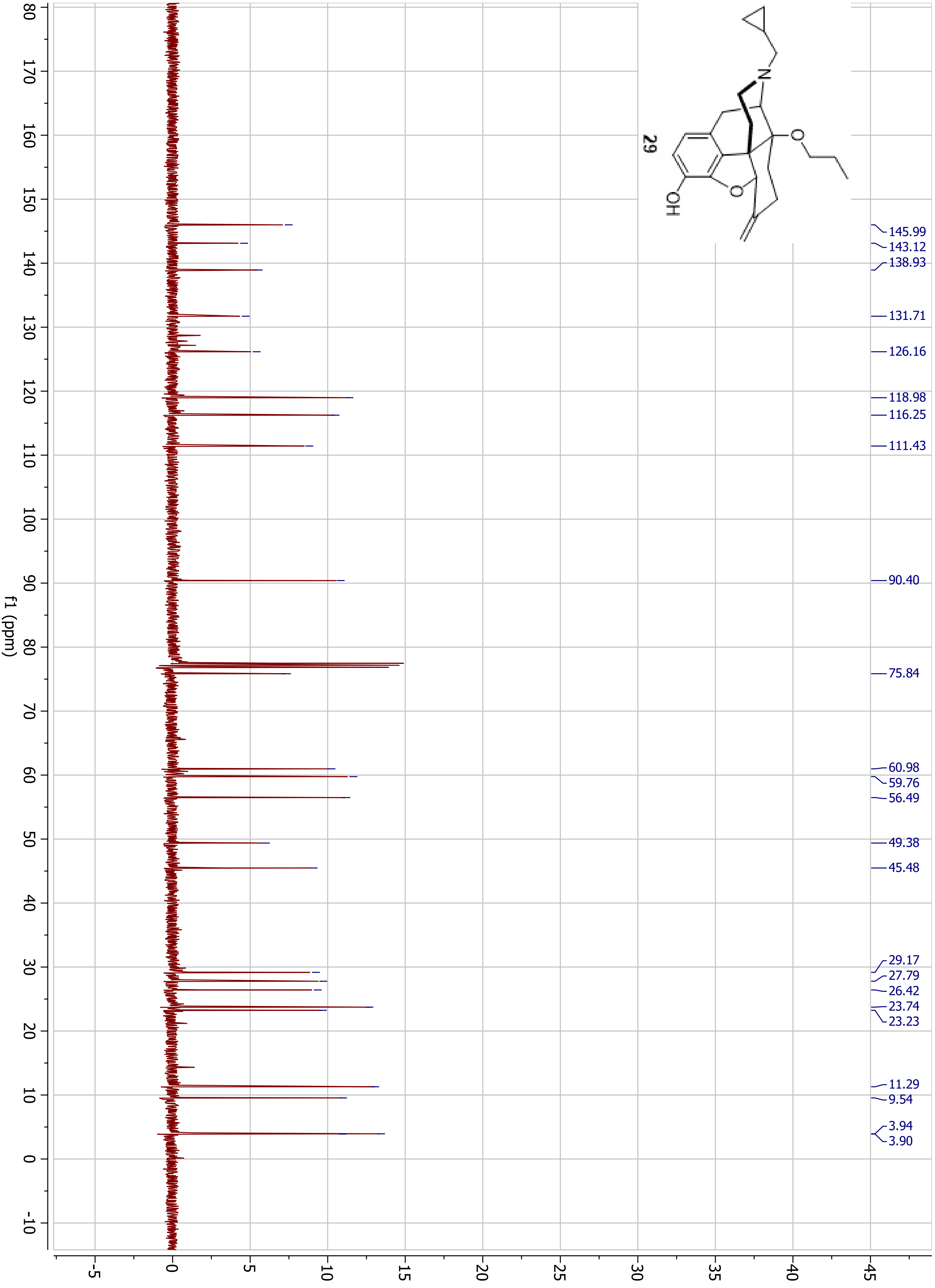
29
OH
O
N
145.99
143.12
138.93
131.71
126.16
118.98
116.25
111.43
90.40
75.84
60.98
59.76
56.49
49.38
45.48
29.17
27.79
26.42
23.74
23.23
11.29
9.54
3.94
3.90
f1 (ppm)

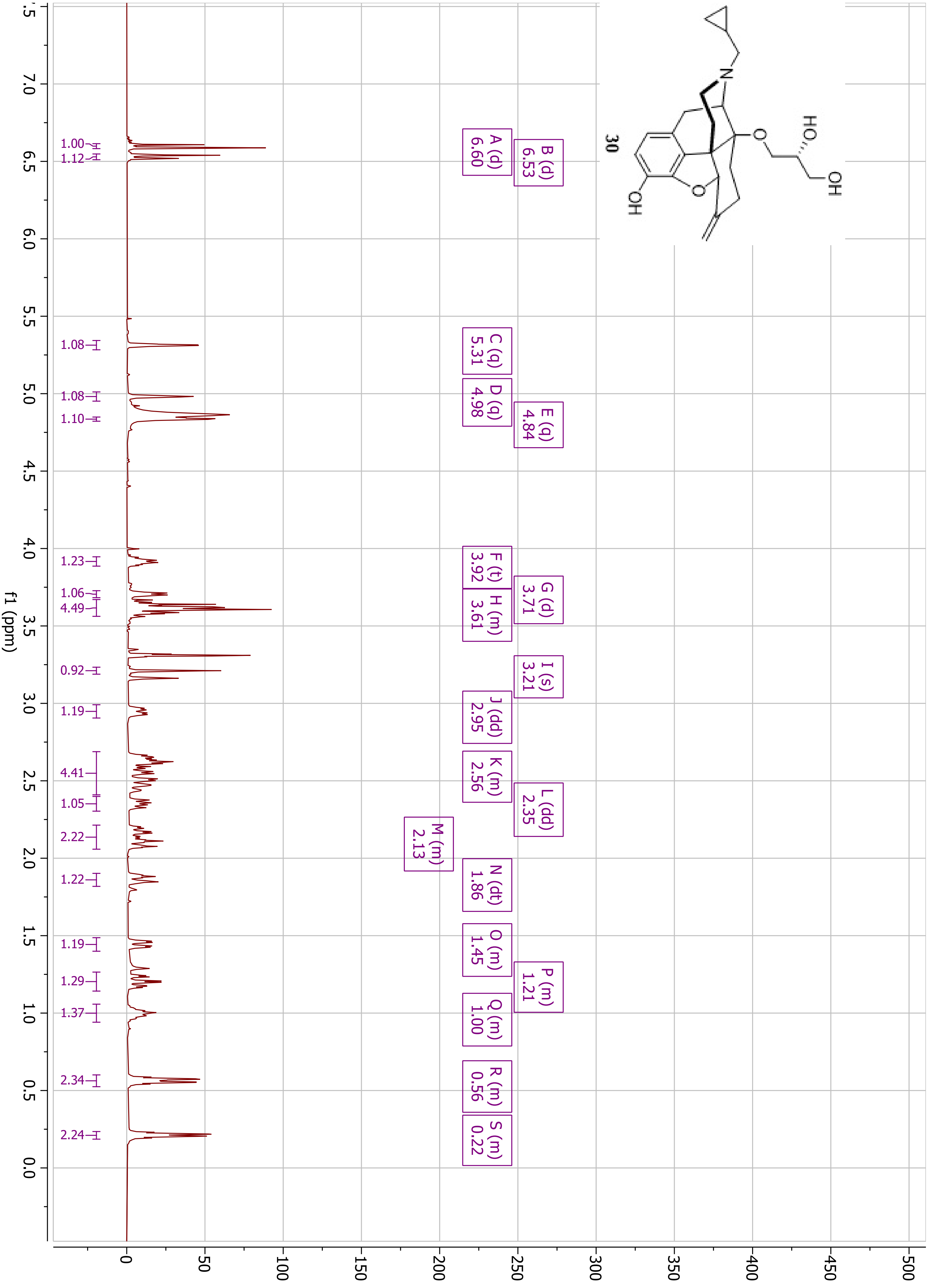
30
HO
OH
OH
O
N
A (d) 6.60
B (d) 6.53
C (q) 5.31
D (q) 4.98
E (q) 4.84
F (t) 3.92
G (d) 3.71
H (m) 3.61
I (s) 3.21
J (dd) 2.95
K (m) 2.56
L (dd) 2.35
M (m) 2.13
N (dt) 1.86
O (m) 1.45
P (m) 1.21
Q (m) 1.00
R (m) 0.56
S (m) 0.22
1.00
1.12
1.08
1.08
1.10
1.23
1.06
4.49
0.92
1.19
4.41
1.05
2.22
1.22
1.19
1.29
1.37
2.34
2.24
f1 (ppm)

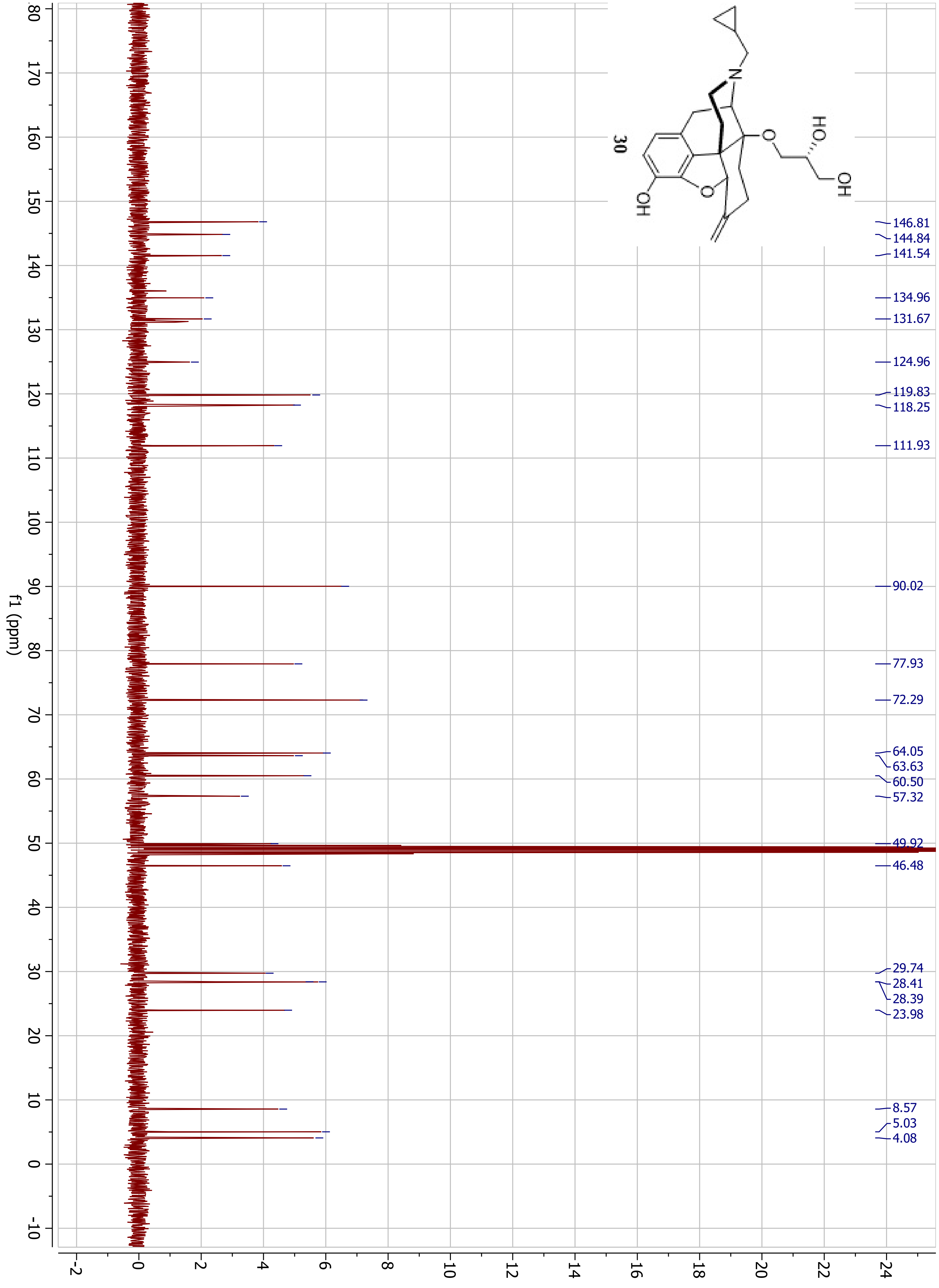
30
HO
OH
OH
N
O
146.81
144.84
141.54
134.96
131.67
124.96
119.83
118.25
111.93
90.02
77.93
72.29
64.05
63.63
60.50
57.32
49.92
46.48
29.74
28.41
28.39
23.98
8.57
5.03
4.08
f1 (ppm)

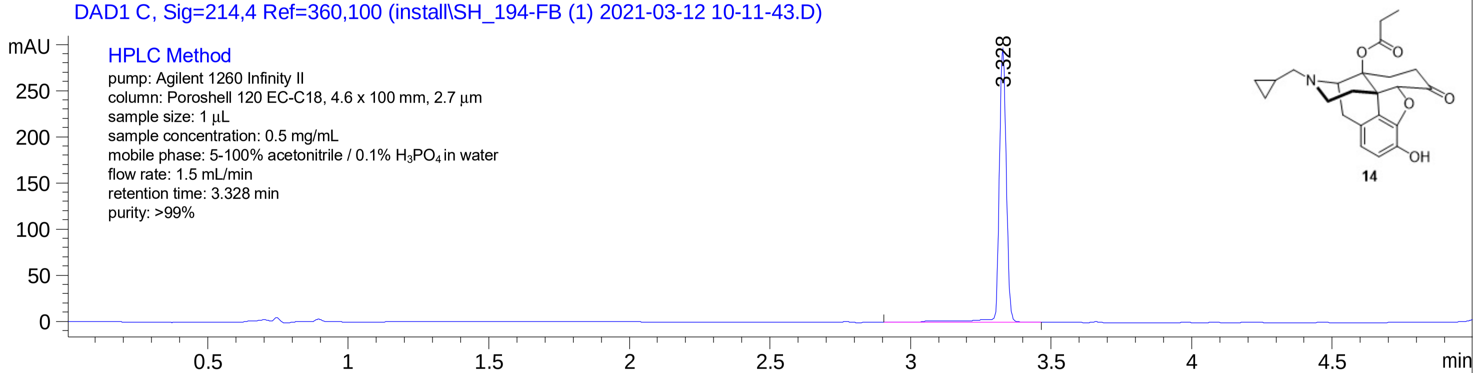
DAD1 C, Sig=214,4 Ref=360,100 (install\SH_194-FB (1) 2021-03-12 10-11-43.D)
mAU
250
200
150
100
50
0
HPLC Method
pump: Agilent 1260 Infinity II
column: Poroshell 120 EC-C18, 4.6 x 100 mm, 2.7 µm
sample size: 1 µL
sample concentration: 0.5 mg/mL
mobile phase: 5-100% acetonitrile / 0.1% H3PO4 in water
flow rate: 1.5 mL/min
retention time: 3.328 min
purity: >99%
3.328
0.5
1
1.5
2
2.5
3
3.5
4
4.5
min
O
O
N
O
O
OH

14

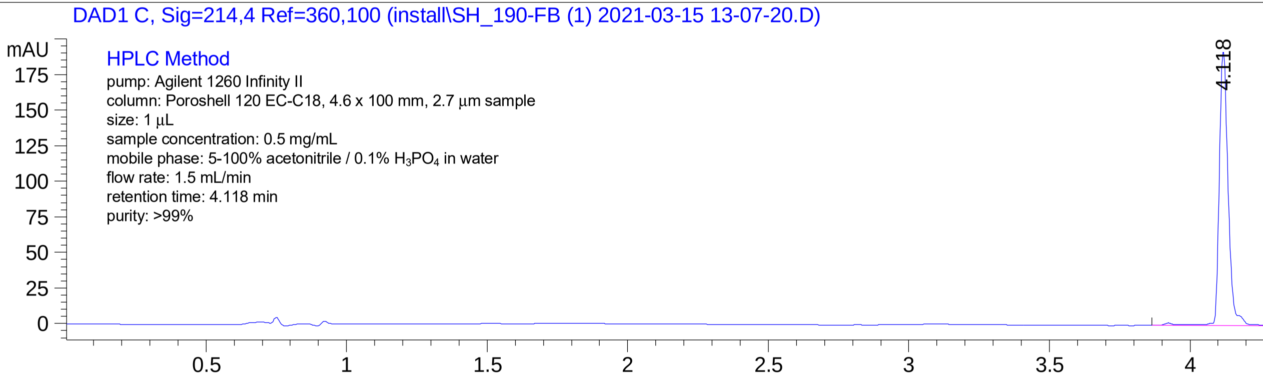
DAD1 C, Sig=214,4 Ref=360,100 (install\SH_190-FB (1) 2021-03-15 13-07-20.D)
mAU
175
150
125
100
75
50
25
0
HPLC Method
pump: Agilent 1260 Infinity II
column: Poroshell 120 EC-C18, 4.6 x 100 mm, 2.7 µm sample
size: 1 µL
sample concentration: 0.5 mg/mL
mobile phase: 5-100% acetonitrile / 0.1% H3PO4 in water
flow rate: 1.5 mL/min
retention time: 4.118 min
purity: >99%
4.118
0.5
1
1.5
2
2.5
3
3.5
4
4.5
min

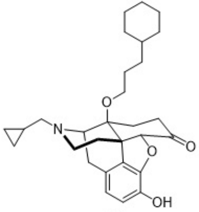
O
N
O
O
OH

16

S32

DAD1 C, Sig=214,4 Ref=360,100 (install\SH_108-FB (1) 2021-03-16 12-19-32.D)

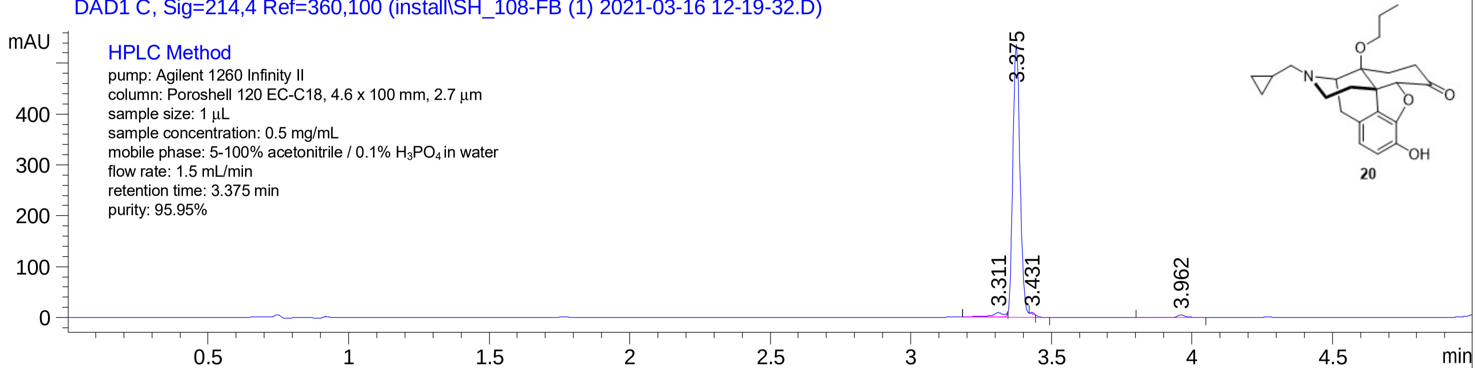

HPLC Method

pump: Agilent 1260 Infinity II
column: Poroshell 120 EC-C18, 4.6 x 100 mm, 2.7 µm
sample size: 1 µL
sample concentration: 0.5 mg/mL
mobile phase: 5-100% acetonitrile / 0.1% H_3PO_4 in water
flow rate: 1.5 mL/min
retention time: 3.375 min
purity: 95.95%

20

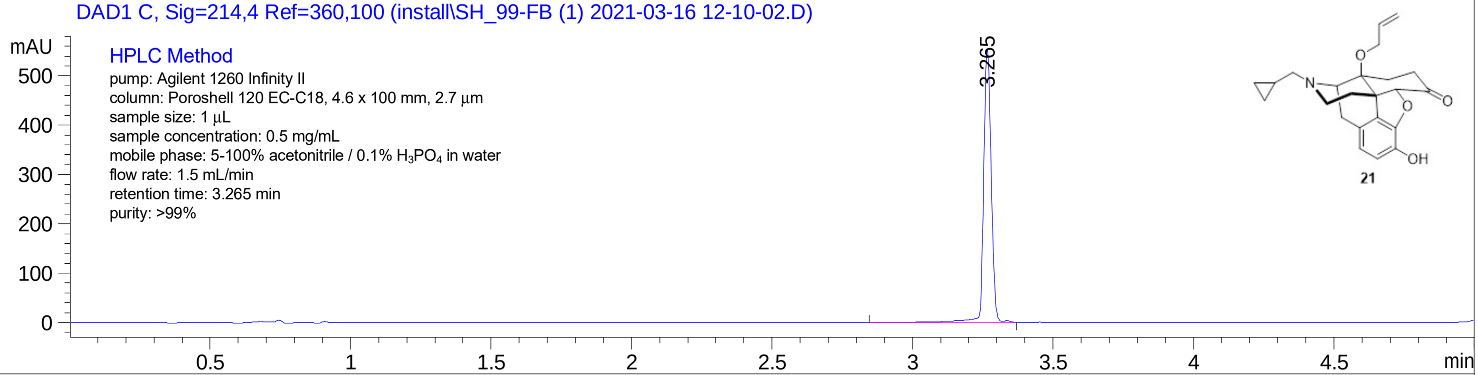
DAD1 C, Sig=214,4 Ref=360,100 (install\SH_99-FB (1) 2021-03-16 12-10-02.D)
HPLC Method
pump: Agilent 1260 Infinity II
column: Poroshell 120 EC-C18, 4.6 x 100 mm, 2.7 µm
sample size: 1 µL
sample concentration: 0.5 mg/mL
mobile phase: 5-100% acetonitrile / 0.1% H_3PO_4 in water
flow rate: 1.5 mL/min
retention time: 3.265 min
purity: >99%
3.265
mAU
500
400
300
200
100
0
0.5
1
1.5
2
2.5
3
3.5
4
4.5
min
N
O
O
O
OH
21

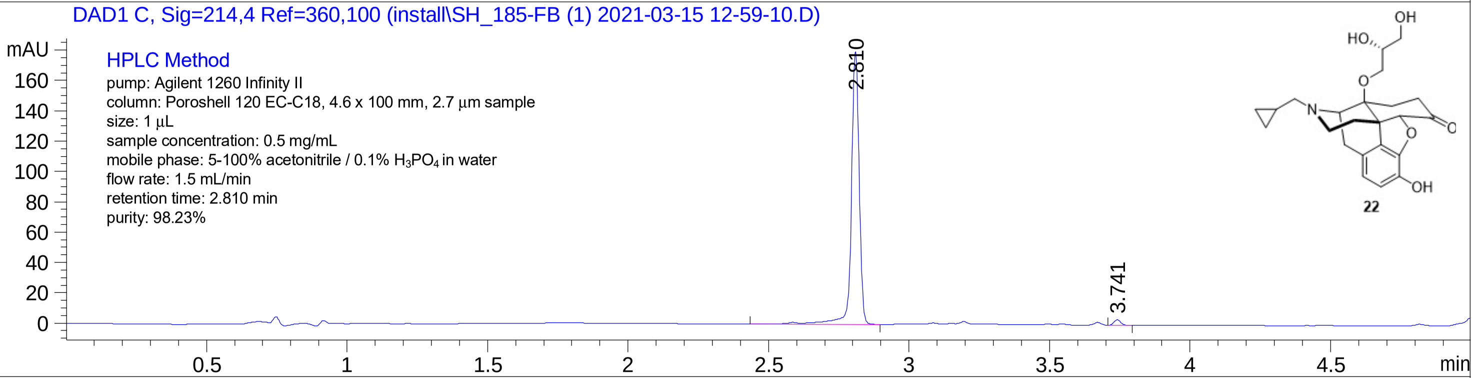
DAD1 C, Sig=214,4 Ref=360,100 (install\SH_185-FB (1) 2021-03-15 12-59-10.D)
mAU
HPLC Method
pump: Agilent 1260 Infinity II
column: Poroshell 120 EC-C18, 4.6 x 100 mm, 2.7 µm sample
size: 1 µL
sample concentration: 0.5 mg/mL
mobile phase: 5-100% acetonitrile / 0.1% H3PO4 in water
flow rate: 1.5 mL/min
retention time: 2.810 min
purity: 98.23%
2.810
3.741
min
22

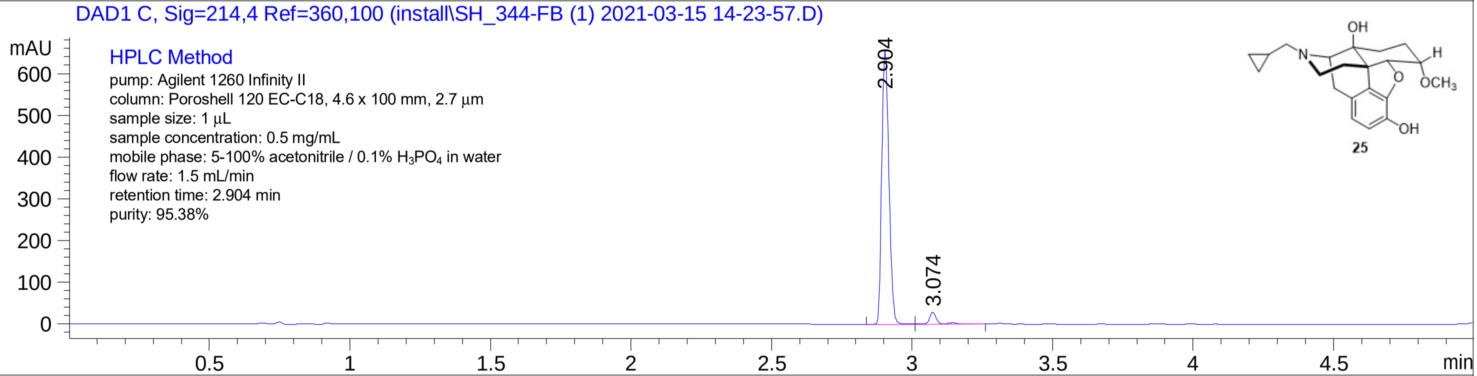
DAD1 C, Sig=214,4 Ref=360,100 (install\SH_344-FB (1) 2021-03-15 14-23-57.D)
mAU
600
500
400
300
200
100
0
HPLC Method
pump: Agilent 1260 Infinity II
column: Poroshell 120 EC-C18, 4.6 x 100 mm, 2.7 µm
sample size: 1 µL
sample concentration: 0.5 mg/mL
mobile phase: 5-100% acetonitrile / 0.1% H_3PO_4 in water
flow rate: 1.5 mL/min
retention time: 2.904 min
purity: 95.38%
2.904
3.074
0.5
1
1.5
2
2.5
3
3.5
4
4.5
min
OH
N
H
O
OCH_3
OH
25

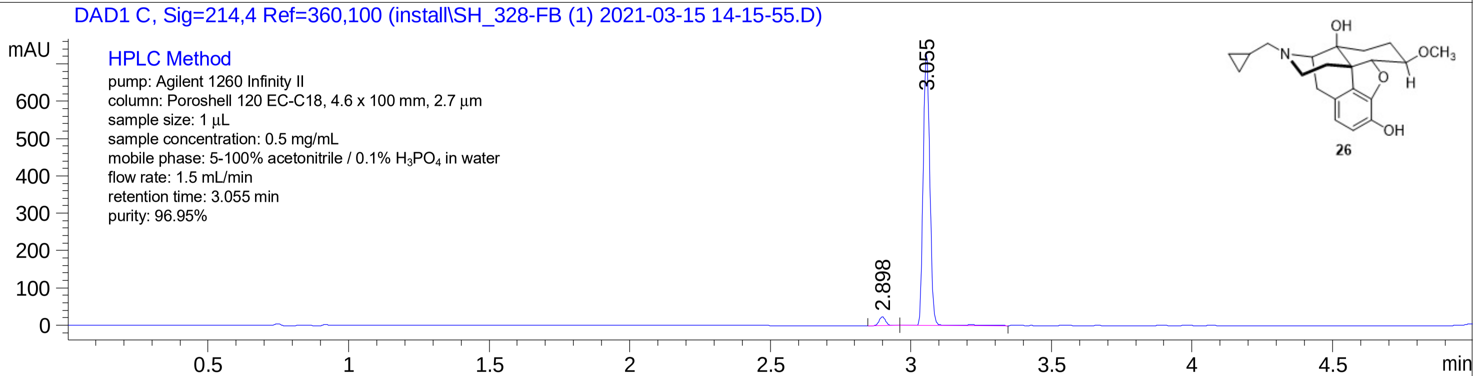
DAD1 C, Sig=214,4 Ref=360,100 (install\SH_328-FB (1) 2021-03-15 14-15-55.D)
mAU
HPLC Method
pump: Agilent 1260 Infinity II
column: Poroshell 120 EC-C18, 4.6 x 100 mm, 2.7 µm
sample size: 1 µL
sample concentration: 0.5 mg/mL
mobile phase: 5-100% acetonitrile / 0.1% H_3PO_4 in water
flow rate: 1.5 mL/min
retention time: 3.055 min
purity: 96.95%
3.055
2.898
600
500
400
300
200
100
0
0.5
1
1.5
2
2.5
3
3.5
4
4.5
min
OH
N
OCH_3
O
H
OH
26

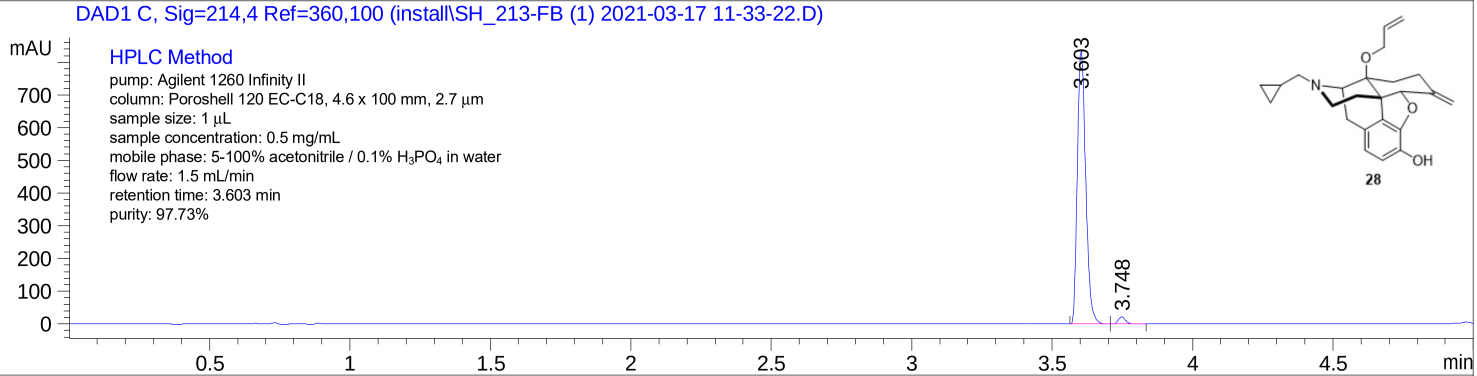
DAD1 C, Sig=214,4 Ref=360,100 (install\SH_213-FB (1) 2021-03-17 11-33-22.D)
mAU
700
600
500
400
300
200
100
0
HPLC Method
pump: Agilent 1260 Infinity II
column: Poroshell 120 EC-C18, 4.6 x 100 mm, 2.7 µm
sample size: 1 µL
sample concentration: 0.5 mg/mL
mobile phase: 5-100% acetonitrile / 0.1% H_3PO_4 in water
flow rate: 1.5 mL/min
retention time: 3.603 min
purity: 97.73%
3.603
3.748
N
O
O
OH
28
0.5
1
1.5
2
2.5
3
3.5
4
4.5
min

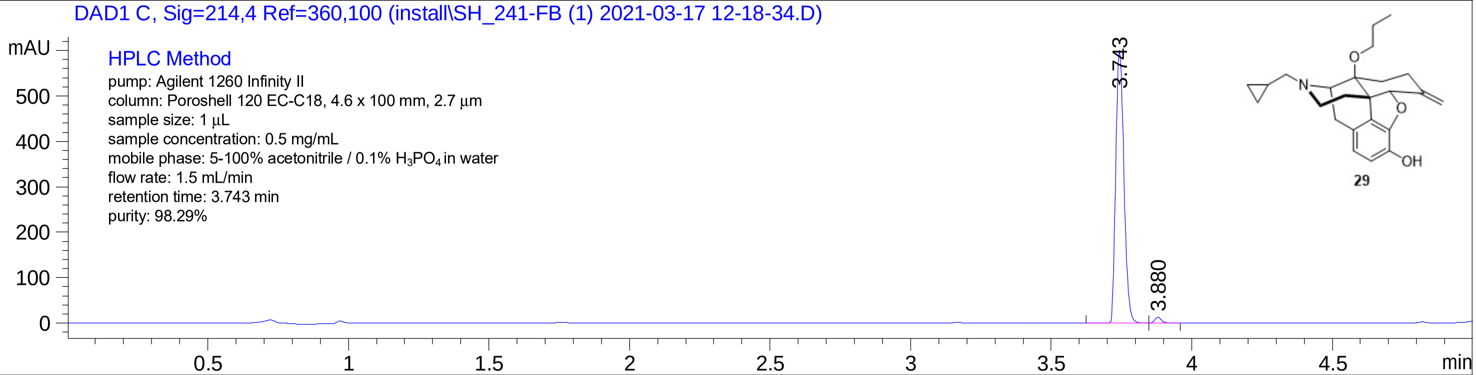
DAD1 C, Sig=214,4 Ref=360,100 (install\SH_241-FB (1) 2021-03-17 12-18-34.D)
mAU
HPLC Method
pump: Agilent 1260 Infinity II
column: Poroshell 120 EC-C18, 4.6 x 100 mm, 2.7 µm
sample size: 1 µL
sample concentration: 0.5 mg/mL
mobile phase: 5-100% acetonitrile / 0.1% H_3PO_4 in water
flow rate: 1.5 mL/min
retention time: 3.743 min
purity: 98.29%
3.743
3.880
OH
29
0
100
200
300
400
500
0.5
1
1.5
2
2.5
3
3.5
4
4.5
min

DAD1 C, Sig=214,4 Ref=360,100 (install\SH_273-FB (1) 2021-03-18 12-09-51.D)

HPLC Method

pump: Agilent 1260 Infinity II
column: Poroshell 120 EC-C18, 4.6 x 100 mm, 2.7 µm
sample size: 1 µL
sample concentration: 0.5 mg/mL
mobile phase: 5-100% acetonitrile / 0.1% H_3PO_4 in water
flow rate: 1.5 mL/min
retention time: 3.219 min
purity: 97.52%

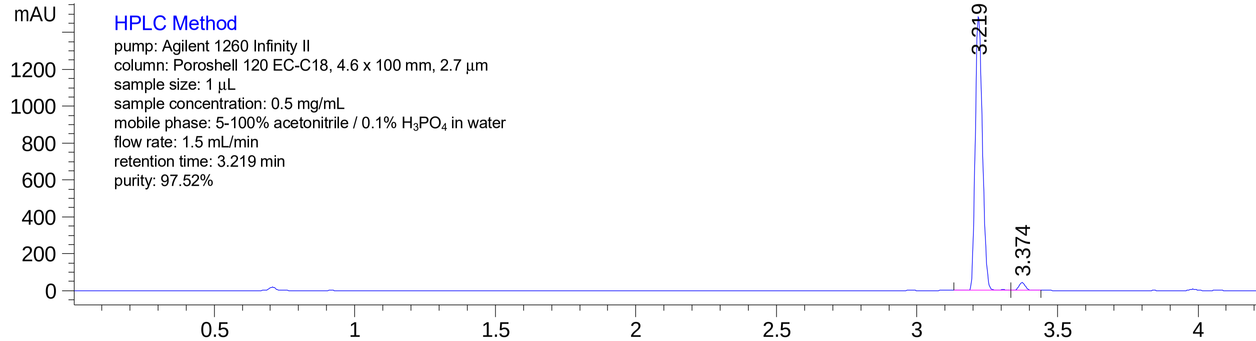

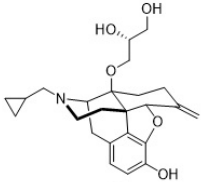

30

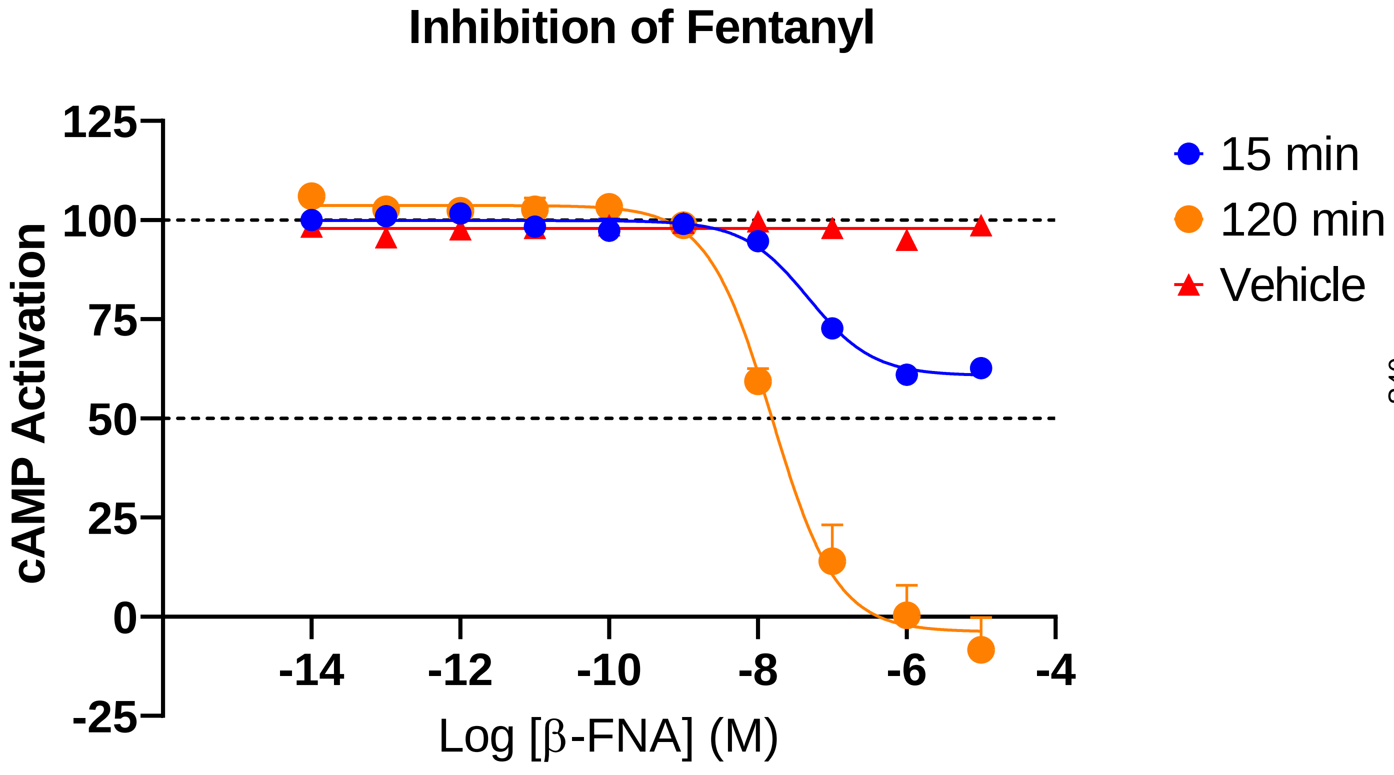
Inhibition of Fentanyl
cAMP Activation
125
100
75
50
25
0
-25
-14
-12
-10
-8
-6
-4
Log [β-FNA] (M)
15 min
120 min
Vehicle
